# Polycationic Ru(II) Luminophores: Syntheses, Photophysics, and Application in Electrostatically Driven Sensitization of Lanthanide Luminescence

**DOI:** 10.1021/acs.inorgchem.3c02352

**Published:** 2023-11-20

**Authors:** Richard C. Knighton, Joseph M. Beames, Simon J. A. Pope

**Affiliations:** † School of Chemistry, Main Building, Cardiff University, Cardiff CF10 3AT, Cymru/Wales, U.K.; ‡ School of Chemistry, University of Birmingham, Birmingham B152TT, England

## Abstract

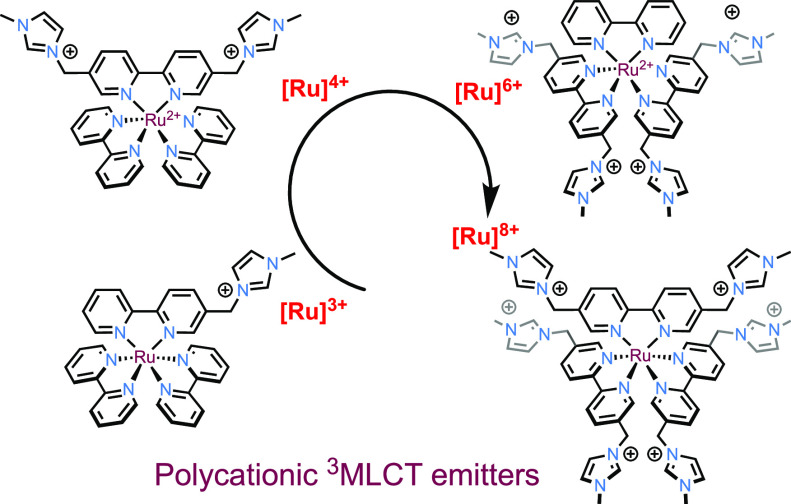

A series of photoluminescent Ru(II) polypyridine complexes have been synthesized in a manner that varies the extent of the cationic charge. Two ligand systems (L1 and L2), based upon 2,2′-bipyridine (bipy) mono- or difunctionalized at the 5- or 5,5′-positions using *N*-methylimidazolium groups, were utilized. The resulting Ru(II) species therefore carried +3, +4, +6, and +8 complex moieties based on a [Ru(bipy)_3_]^2+^ core. Tetra-cationic [Ru(bipy)_2_(**L2**)][PF_6_]_4_ was characterized using XRD, revealing H-bonding interactions between two of the counteranions and the cationic unit. The ground-state features of the complexes were found to closely resemble those of the parent unfunctionalized [Ru(bipy)_3_]^2+^ complex. In contrast, the excited state properties produce a variation in emission maxima, including a bathochromic 44 nm shift of the ^3^MLCT band for the tetra-cationic complex; interestingly, further increases in overall charge to +6 and +8 produced a hypsochromic shift in the ^3^MLCT band. Supporting DFT calculations suggest that the trend in emission behavior may, in part, be due to the precise nature of the LUMO and its localization. The utility of a photoactive polycationic Ru(II) complex was then demonstrated through the sensitization of a polyanionic Yb(III) complex in free solution. The study shows that electrostatically driven ion pairing is sufficient to facilitate energy transfer between the ^3^MLCT donor state of the Ru(II) complex and the accepting ^2^F_5/2_ excited state of Yb(III).

## Abstract 2

Heteroleptic Ru(II) complexes based upon methyl imidazolium functionalized 2,2′-bipyridine ligands can be synthesized with overall charges of +3, +4, +6, and +8. Each of the complexes was photoluminescent through a ^3^MLCT emitting state. The utility of high overall cationic charge and light harvesting ability was demonstrated in solution through electrostatically driven d-f energy transfer to near-IR emitting Yb(III).

## Introduction

The study of photoactive transition-metal complexes is a rich and diverse area of chemistry and has important applications in many areas, such as light harvesting,^1–3^ sensing,^4,5^ energy upconversion,^6–8^ bioimaging,^4^ and theranostics.^10,11,11^ While interest in earth-abundant luminescent complexes^13^ has understandably, from a sustainability perspective, attracted significant attention in the past few years, precious metals still provide an excellent basis for the rational design of efficient photoluminescent compounds.

Our interest has been drawn to the study of (poly)cationic luminescent complexes and their potential applications. In particular, cationic complexes are important in host–guest studies;^14^ as components of ionic liquids;^15^ ion pairing;^16^ in the development of sensors and DNA binding;^17^ as photoactive components of aggregation colloids such as micelles and microemulsions;^18^ and, in membrane transport studies.^19^ However, luminescent metal complexes with high magnitudes of overall positive charge are very rare. Therefore, we were interested in investigating the synthesis and physical properties of a series of cationic Ru(II) complexes that are structurally related to the archetypal triplet metal to ligand charge transfer (^3^MLCT) emitter [Ru(bipy)_3_]^2+^ (where bipy = 2,2′-bipyridine).

Recently, the early history of bipy has been retrospectively reviewed by Constable and Housecroft^20^ providing an important reminder of the ubiquity and ongoing application of this ligand and its close relatives. Ligand modification is the obvious route for the consideration of polycationic luminescent Ru(II) complexes, and our approach, described herein, was facilitated by the use of cationic bipy ligands. Interestingly, anionic bipy ligands are more common in the literature, for example carboxylated (some of the earliest reported variants of bipy^21^) or phosphonated^22^ derivatives of bipy are very well known and have been widely exploited in coordination chemistry.^23^ Although rarer, cationic bipyridines have been reported and complexed with Ru(II). Typically, these ligands incorporate terminal amine groups that can be protonated in certain media, allowing their development as luminescent sensors, or for consideration in photodynamic therapy, which exploits the inherent phototoxicity of the Ru(II) complex.^24^ Meyer and co-workers reported a series of Ru(II) complexes with quaternary alkylated amine substituents at the 4,4′ positions of the bipy framework; the complexes were studied in the context of Ru-based dye-sensitized solar cells where the effect of charge and ion pairing with iodide is especially important.^25^ Similar cationic ligands have been deployed in heteroleptic Ru(II) complexes that show a very high affinity for DNA (via intercalation) and subsequent in vitro activity in antitumor experiments.^26^

An additional aim of the current work on polycationic Ru(II) complexes was to investigate whether such species could be used as sensitizers of trivalent lanthanide excited states. In that context, it is now two decades since transition metal complexes were first demonstrated^27^ to be viable chromophores for sensitized lanthanide luminescence. In particular, MLCT species such as derivatives of the aforementioned [Ru(bipy)_3_]^2+^ can sensitize lanthanide ions (for example, Nd(III), Yb(III), and Er(III), all of which are near-IR emitters) which possess accepting excited states that are compatible with the donor ^3^MLCT energy level. Typically the lanthanide excited state is populated through energy transfer mechanisms.^28^ Generally, these mixed-metal, so-called d-f hybrids,^29^ have been obtained through covalently linked ditopic ligands,^30^ or self-assembly of complex components,^31^ including through supramolecular host–guest interactions.^32^ This current work demonstrates that favorable electrostatic interactions in solution can be manipulated to promote energy transfer between polycationic Ru(II) and anionic Yb(III) complexes.

## Experimental Section

^1^H and ^13^C{^1^H} NMR spectra were recorded on an NMR-FT Bruker 500 MHz spectrometer and recorded in CDCl_3_, acetonitrile-*d*_3_, and DMSO-*d*_6_. ^1^H and ^13^C{^1^H} NMR chemical shifts (δ) were determined relative to residual solvent peaks with digital locking and are given in ppm. Coupling constants are quoted in Hz. High-resolution mass spectra were obtained at Cardiff University. UV–vis studies were performed on a Shimadzu UV-1800 spectrophotometer as MeCN solutions (1 × 10^–5^ M). Photophysical data were obtained on a JobinYvon–Horiba Fluorolog spectrometer fitted with a JY TBX picosecond photodetection module. A Hamamatsu R5509–73 detector (cooled to −80 °C using a C9940 housing) was used for near-IR measurements. Luminescence lifetime profiles were obtained using the JobinYvon–Horiba FluoroHub single photon counting module, and the data fits yielded the lifetime values using the provided DAS6 deconvolution software. The pulsed source was a nano-LED configured for 295 nm output operating at 1 MHz; for the near-IR Yb(III) lifetimes, the pulsed laser source was a Continuum Minilite Nd:YAG configured for 355 nm output. Quantum yield measurements were obtained using comparative actinometry on aerated MeCN solutions of the complexes using [Ru(bipy)_3_](PF_6_)_2_ in aerated MeCN as a standard (Φ = 0.018).^33^ Elemental analyses were conducted by the EA Services Team at the London Metropolitan University.

## Cyclic Voltammetry

Cyclic voltammetry was performed by using a PalmSens4 potentiostat. Experiments were performed using HPLC grade CH_2_Cl_2_ with an analyte concentration of 1 mM at 293 K, using triply recrystallized [^n^Bu_4_N][PF_6_] as the supporting electrolyte at 0.1 M concentration. A three-electrode setup was used, consisting of a platinum disc working electrode, a platinum wire counter-electrode, and a silver wire pseudoreference. Solutions were sparged for 10 min with a CH_2_Cl_2_ saturated stream of nitrogen gas. Voltammograms were referenced to the ferrocene/ferrocenium redox couple measured by using the same conditions.

## Computational Methods

Electronic structure calculations were performed using density functional theory within the Gaussian 09 computational chemistry suite.^34^ All calculations were performed with the Stuttgart–Dresden (SDD) effective core potential and basis set in the treatment of the ruthenium,^35^ in combination with a 6-31G* basis set for all other light atoms.^36^ Geometry optimizations were performed for all cationic species, and minima were confirmed as stationary points through the computation of harmonic vibrational frequencies, each of which showed no imaginary components. These stationary points were used in single-point TD-DFT calculations to compute vertical excitation energies. Calculations were performed on isolated complexes without implicit solvent due to substantial difficulties in converging both SCF and geometry optimization components of the higher charge complexes through the SCRF formalization.

Phosphorescence and spin-forbidden absorption band positions are estimated using triplet-inclusive TD-DFT calculations. Decomposition of the molecular orbital character was performed using the GaussSum software package.^37^

## X-ray Crystallography

### Data Collection and Processing

A suitable crystal was selected and mounted on a nylon loop with Fomblin oil and placed on a Rigaku Oxford Diffraction SuperNova diffractometer with a dual source (Cu at zero) equipped with an AtlasS2 CCD area detector at 293(2) K. The crystal was kept at a steady *T* of 293(2) K during data collection. The structure was solved with the SHELXT^38^ structure solution program using the Intrinsic Phasing solution method and by using Olex2^39^ as the graphical interface. The model was refined with SHELXL^40^ using the least-squares minimization. CCDC 2250505 contains the supplementary X-ray crystallographic data for [Ru(bipy)_2_(**L2**)][PF_6_]_4_. These data can be obtained free of charge from the Cambridge Crystallographic Data Centre via www.ccdc.cam.ac.uk/data_request/cif., or from the Cambridge Crystallographic Data Centre, Union Road, Cambridge, CB2 1EZ; fax(+44) 1223–336–033 or email: deposit@ccdc.cam.ac.uk.

### Synthesis of the Ligands

#### Synthesis of 3-([2,2′-Bipyridin]-5-ylmethyl)-1-methyl-1*H*-imidazol-3-ium Chloride (**L1**)

5-chloromethyl-2,2′-bipyridine (800 mg, 3.91 mmol, 1.0 equiv) was dissolved in 1-methylimidazole (24 mL) and heated under N_2_ at 100 °C for 3 h. The reaction was cooled to ambient temperature, and Et_2_O (72 mL) was added. The mixture was cooled to 4 °C for 18 h, filtered, washed with Et_2_O (2 × 10 mL), and dried in vacuo to obtain the compound as a hygroscopic deliquescent off-white solid (0.870 g, 3.03 mmol, 78%). ^1^H NMR spectrum (500 MHz, 293 K, CD_3_OD): 9.17 (s, 1H, ImH, 97% deuterated in CD_3_OD), 8.78 (s, 1H, bipyH), 8.66 (d, ^3^*J*_HH_ = 4.7, 1H, bipyH), 8.38 (d, ^3^*J*_HH_ = 8.4, 1H, bipyH), 8.36 (d, ^3^*J*_HH_ = 8.0, 1H, bipyH), 8.02 (d, ^3^*J*_HH_ = 8.2, ^4^*J*_HH_ = 2.2, 1H, bipyH), 7.94 (app. t, *J*_HH_ = 7.7, 1H, bipyH), 7.75 (s, 1H, ImH), 7.65 (s, 1H, ImH), 7.47–7.34 (m, 1H, bipyH), 5.60 (s, 2H, ArCH_2_), 3.97 (s, 3H, NCH_3_); ^13^C{^1^H} NMR spectrum (126 MHz, 293 K, CD_3_OD): 157.9 (s, bipy), 156.5 (s, bipy), 156.4 (s, bipy), 150.4 (s, bipy), 138.8 (s, bipy), 138.7 (s, bipy), 138.1 (t, ^1^*J*_CD_ = 34, Im deuterated), 131.6 (s, bipy), 125.7 (s, bipy), 125.5 (s, Im), 123.7 (s, Im), 122.7 (s, bipy), 122.6 (s, bipy), 51.2 (s, ArCH_2_), 36.7 (s, NCH_3_), only the deuterated imidazolium resonance observed. Elemental analysis: found: C 60.75%, H 5.24%, N 18.47%; calcd for C_15_H_15_N_4_Cl_0.5_H_2_O, C 60.91%, H 5.45%, N 18.94%. ESI-MS *m*/*z* calcd for [C_15_H_15_N_4_]^+^, 251.1297; found, 251.1301. IR (ATR): ν_max_ 3382, 3069, 2943, 2859, 1574, 1460, 1177, 1024, 847 cm^–1^.

#### Synthesis of 3,3′-([2,2′-Bipyridine]-5,5′-diylbis(methylene))bis(1-methyl-1*H*-imidazol-3-ium) Dichloride (**L2**)

5,5′-bischloromethyl-2,2′-bipyridine (870 mg, 3.44 mmol, 1.0 equiv) was dissolved in 1-methylimidazole (30 mL) and heated to 100 °C for 18 h. The reaction was cooled to ambient temperature and Et_2_O (90 mL) was added. The reaction was stirred for 10 min, filtered, and the solid was washed with Et_2_O (2 × 60 mL) to obtain the product as a hygroscopic off-white solid (1.43 g, 0.34 mmol, >99%). ^1^H NMR spectrum (500 MHz, 293 K, CD_3_OD): 9.14–9.11 (m, 2H, ImH, 25% deuterated), 8.80–8.75 (m, 2H, bipyH), 8.46 (dd, ^3^*J*_HH_ = 8.2, ^4^*J*_HH_ = 0.8, 2H, bipyH), 8.04–7.99 (m, 2H, bipyH), 7.73 (app.t, *J*_HH_ = 1.9, 2H, ImH), 7.64 (app.t, *J*_HH_ = 1.9, 2H, ImH), 5.58 (s, 4H, ArCH_2_), 3.96 (s, 6H, NCH_3_); ^13^C{^1^H} NMR spectrum (126 MHz, 293 K, CD_3_OD): 156.9 (s, bipy), 150.6 (s, bipy), 139.0 (s, bipy), 138.7–137.9 (m, Im-deuterated), 137.5 (s, Im–nondeuterated), 132.1 (s, bipy), 125.5 (s, Im), 123.6 (s, Im), 122.6 (s, bipy), 51.1(s, ArCH_2_), 36.8 (NCH_3_) ppm. ESI-MS *m*/*z* calcd for [C_20_H_22_N_6_]^+^, 381.1595; found, 381.1590. IR (ATR): ν_max_ 3354, 3055, 2945, 2858, 1576, 1557, 1454, 1174, 1024, 785 cm^–1^.

### Synthesis of the Complexes

#### Synthesis of [Ru(bipy)_2_(**L1**)][PF_6_]_3_

A Schlenk flask was charged with 3,3′-([2,2′-bipyridine]-5,5′-diylbis(methylene))bis(1-methyl-1*H*-imidazol-3-ium) dichloride (103.5 mg, 0.248 mmol, 1.05 equiv) and [Ru(bipy)_2_Cl_2_] (114.4 mg, 0.236 mmol, 1.0 equiv) under N_2_. Ethylene glycol (2 mL) was added, and the solvent was sparged with N_2_ for 15 min. The reaction was heated to reflux for 3 h after which the reaction was cooled and NH_4_PF_6(aq)_ (0.1 M, 10 mL) was added. The solid was filtered, and the crude material was purified by column chromatography (SiO_2_; MeCN/H_2_O/sat. KNO_3(aq)_; 20:2:1 → 14:2:1). The organic solvent was removed, and the product precipitated via the addition of NH_4_PF_6(aq)_ (0.1 M, 10 mL). The solid was filtered and washed with H_2_O (2 × 2 mL) and dried to give the title compound as a red solid (132 mg, 0.098 mmol, 60%). ^1^H NMR spectrum (500 MHz, 293 K, CD_3_CN): 8.55–8.47 (m, 6H, bipyH), 8.43–8.40 (m, 1H, ImH), 8.11–8.02 (m, 6H, bipyH), 7.92 (dd, ^3^*J*_HH_ = 8.4, ^4^*J*_HH_ = 2.1, 1H, bipyH), 7.78 (ddt, ^3^*J*_HH_ = 5.5, ^4^*J*_HH_ = 1.5, 0.7, 1H, bipyH), 7.76–7.67 (m, 4H, bipyH), 7.52 (m, 1H, bipyH), 7.45–7.38 (m, 5H, bipyH), 5.29 (d, ^2^*J*_HH_ = 16.0, 1H, ArCH_2_), 5.24 (d, ^2^*J*_HH_ = 16.0, 1H, ArCH_2_), 3.83 (s, 3H, ImCH_3_); ^13^C{^1^H} NMR spectrum (126 MHz, 293 K, CD_3_CN): 158.3 (s, bipy), 158.0 (s, bipy), 158.0 (s, bipy), 157.4 (s, bipy), 153.0 (s, bipy), 153.0 (s, bipy), 152.9 (s, bipy), 152.8 (s, bipy), 152.7 (s, bipy), 152.6 (s, bipy), 151.4 (s, bipy), 138.9 (s, bipy), 138.9 (s, bipy), 138.9 (s, bipy), 138.8 (s, bipy), 138.0 (s, bipy),137.9 (s, Im), 135.1 (s, bipy), 128.9 (s, bipy), 128.6 (s, bipy), 128.6 (s, bipy), 125.8 (s, Im), 125.4 (s, bipy), 125.4 (s, Im), 125.3 (s, bipy), 125.3 (s, bipy), 125.2 (s, bipy), 123.5 (s, bipy), 118.4 (s, bipy), 50.2 (s, ArCH_2_), 37.1 (s, ImCH_3_) ppm. ESI-MS *m*/*z* calcd for [C_35_H_31_F_12_N_8_P_2_Ru]^+^, 955.1008; found, 955.1010. IR (ATR): ν_max_ 1466, 1445, 758, 729, 648, 623, 556, 419 cm^–1^.

#### Synthesis of [Ru(bipy)_2_(**L2**)][PF_6_]_4_

A Schlenk flask was charged with 3-([2,2′-bipyridin]-5-ylmethyl)-1-methyl-1*H*-imidazol-3-ium chloride (34.7 mg, 0.121 mmol, 1.05 equiv) and [Ru(bipy)_2_Cl_2_] (55.8 mg, 0.115 mmol, 1.0 equiv) under N_2_. Ethylene glycol (1 mL) was added, and the solvent was sparged with N_2_ for 15 min. The reaction was heated to reflux for 3 h after which the reaction was cooled and NH_4_PF_6(aq)_ (0.1 M, 10 mL) was added. The solid was filtered, and the crude material was purified by column chromatography (SiO_2_; MeCN/H_2_O/sat. KNO_3(aq)_; 14:2:1). The organic solvent was removed in vacuo and the product precipitated via the addition of NH_4_PF_6(aq)_ (0.1 M, 10 mL). The solid was filtered and washed with H_2_O (2 × 2 mL) and dried to give the title compound as a red solid (76 mg, 0.069 mmol, 42%). ^1^H NMR spectrum (500 MHz, 293 K, CD_3_CN): 8.55–8.45 (m, 6H, bipyH), 8.38 (s, 2H, ImH), 8.11–8.05 (m, 4H, bipyH), 7.91 (dd, ^3^*J*_HH_ = 8.5, ^4^*J*_HH_ = 2.0, 2H, bipyH), 7.72 (ddd, ^3^*J*_HH_ = 5.6, ^4^*J*_HH_ = 1.5, 0.7, 2H, bipyH), 7.65 (ddd, ^3^*J*_HH_ = 5.6, ^4^*J*_HH_ = 1.5, 0.7, 2H), 7.53–7.48 (m, 2H, bipyH), 7.43 (ddd, ^3^*J*_HH_ = 7.7, 5.6, ^4^*J*_HH_ = 1.3, 2H), 7.39 (ddd, ^3^*J*_HH_ = 7.7, 5.6, ^4^*J*_HH_ = 1.3, 2H), 7.34 (app.t, *J*_app_ = 1.8, 2H, ImH), 7.20 (app.t, *J*_app_ = 1.9, 2H, ImH), 5.27 (d, ^2^*J*_HH_ = 16.3, 2H, ArCHH), 5.23 (d, ^2^*J*_HH_ = 16.3, 2H, ArCHH), 3.82 (s, 6H, ImCH_3_); ^13^C{^1^H} NMR spectrum (126 MHz, 293 K, CD_3_CN), 157.9 (s, bipy), 157.9 (s, bipy), 157.7 (s, bipy), 152.9 (s, bipy), 152.7 (s, bipy), 151.5 (s, bipy), 139.0 (s, bipy), 138.9 (s, bipy), 138.0 (s, bipy), 137.9 (s, Im), 135.4 (s, bipy), 128.7 (s, bipy), 128.6 (s, bipy), 125.6 (s, bipy), 125.4 (s, Im), 125.3 (s, bipy), 123.5 (s, Im), 50.2 (s, ArCH_2_), 37.1 (s, ImCH_3_) ppm. ESI-MS *m*/*z* calcd for [C_40_H_38_N_10_Ru]^+^, 190.0584; found, 190.0583. IR (ATR): ν_max_ 1466, 1446, 1167, 759, 642, 623, 556, 470, 419 cm^–1^.

#### Synthesis of [Ru(**L2**)_2_Cl_2_]Cl_4_

A Schlenk flask was charged with [Ru(DMSO)_4_Cl_2_] (69.7 mg, 0.144 mmol, 1.0 equiv), LiCl (48.8 mg, 1.15 mmol, 8.0 equiv), and 3-([2,2′-bipyridin]-5-ylmethyl)-1-methyl-1*H*-imidazol-3-ium chloride (120 mg, 0.288 mmol, 2.0 equiv) under N_2_. EtOH (10 mL) was added, and the solution was sparged with N_2_ for 15 min. The reaction was heated to reflux for 18 h, after which it was cooled and the solvent was removed in vacuo. The crude material was purified by column chromatography (Al_2_O_3_; acetone/H_2_O; 100:0 → 50:50) and the solvent was removed in vacuo to give the title compound as a purple solid (20 mg, 0.020 mmol, 14%). ^1^H NMR spectrum (300 MHz, 293 K, CD_3_OD) 9.89 (d, ^4^*J*_HH_ = 2.0, 2H, bipyH), 8.66 (d, ^3^*J*_HH_ = 8.6, 2H, bipyH), 8.50 (d, ^3^*J*_HH_ = 8.4, 2H, bipyH), 8.13 (dd, ^3^*J*_HH_ = 8.4, ^4^*J*_HH_ = 2.1, 1H, bipyH), 7.91 (d, ^3^*J*_HH_ = 2.0, 2H, ImH), 7.85 (d, ^3^*J*_HH_ = 1.8, 2H, ImdH), 7.73 (dd, ^3^*J*_HH_ = 8.5, ^4^*J*_HH_ = 2.0, 2H, bipyH), 7.69 (d, ^3^*J*_HH_ = 2.0, 2H, ImH), 7.61 (d, ^3^*J*_HH_ = 2.0, 2H, ImH), 7.56 (d, ^3^*J*_HH_ = 2.0, 2H. ImH), 5.85 (d, ^2^*J*_HH_ = 15.4, 2H, ArCH_2_), 5.76 (d, ^2^*J*_HH_ = 15.4, 2H, ArCH_2_), 5.39 (s, 4H, ArCH_2_), 3.99 (s, 6H, ImCH_3_), 3.92 (s, 6H, ImCH_3_). The remaining imidazolium N–CH–N protons were undetected due to deuteration. ^13^C{^1^H} NMR spectrum (126 MHz, 293 K, CD_3_OD): 161.8 (s, bipy), 159.9 (s, bipy), 154.0 (s, bipy), 153.7 (s, bipy), 139.0–137.9 (m, ImCD, deuterated), 136.7 (s, bipy), 135.6 (s, bipy), 125.5 (s, Im), 125.4 (s, Im), 124.9 (s, bipy), 124.7 (s, bipy), 124.2 (s, Im), 123.8 (s, Im), 51.2 (s, ArCH_2_), 50.2 (s, ArCH_2_), 36.9 (s, ImCH_3_), 36.8 (s, ImCH_3_) ppm. ESI-MS *m*/*z* calcd for [C_40_H_44_Cl_2_N_12_Ru]^4+^, 216.0559; found, 216.0572.

#### Synthesis of [Ru(bipy)(**L2**)_2_][PF_6_]_6_

A Schlenk flask was charged with [Ru(bipy)(*p*-cymene)Cl]Cl (54.5 mg, 0.114 mmol, 1.0 equiv) and 3-([2,2′-bipyridin]-5-ylmethyl)-1-methyl-1*H*-imidazol-3-ium chloride (100 mg, 0.239 mmol, 2.1 equiv) under N_2_. 2-Ethoxyethanol (1 mL) was added, and the reaction was sparged with N_2_ for 15 min. The reaction was heated to reflux for 48 h after which the reaction was cooled and the compound precipitated via the addition of NH_4_PF_6(aq)_ (0.1 M, 10 mL). The solid was filtered and washed with H_2_O (2 × 5 mL). The crude mixture was purified by column chromatography (SiO_2_; MeCN/H_2_O/sat. KNO_3(aq)_; 14:2:1 → 9:10:1). The organic solvent was removed in vacuo and the compound precipitated via the addition of NH_4_PF_6(aq)_ (0.1 M, 10 mL). The solid was filtered and washed with H_2_O (2 × 5 mL) and dried in vacuo to give the title compound as a red solid (75 mg, 0.041 mmol, 36%). ^1^H NMR spectrum (500 MHz, 293 K, CD_3_CN) 8.54–8.49 (m, 4H, bipyH), 8.46 (dt, ^3^*J*_HH_ = 8.1, ^4^*J*_HH_ = 1.1, 2H), 8.44–8.40 (m, 2H, ImH), 8.37 (m, 2H, ImH), 8.10 (app.td, *J*_app_ = 7.9, ^4^*J*_HH_ = 1.5, 2H, bipyH), 7.89 (dd, ^3^*J*_HH_ = 8.6, ^4^*J*_HH_ = 1.9, 2H, bipyH), 7.85 (dd, ^3^*J*_HH_ = 8.5, ^4^*J*_HH_ = 2.0, 2H, bipyH), 7.69–7.66 (m, 2H, bipyH), 7.64–7.61 (m, 2H, bipyH), 7.52–7.50 (m, 2H, bipyH), 7.43–7.40 (m, 2H, bipyH), 7.39 (app.t, *J*_app_ = 1.7, 2H, ImH), 7.36 (app.t, *J*_app_ = 1.7, 2H, ImH), 5.26 (d, ^2^*J*_HH_ = 16.1, 2H, ArCHH), 5.25 (s, 4H, ArCdH_2_), 5.23 (d, ^2^*J*_HH_ = 16.2, 2H, ArCHH), 3.85 (s, 6H, ImCH_2_), 3.83 (s, 6H, ImCH_2_); ^13^C{^1^H} NMR spectrum (126 MHz, 293 K, CD_3_CN) 157.6 (s, bipy), 157.6 (s, bipy), 157.5 (s, bipy), 153.0 (s, bipy), 151.9 (s, bipy), 151.5 (s, bipy), 139.0 (s, bipy), 138.1 (s, bipy), 137.9 (s, bipy), 137.8 (s, Im), 135.3 (s, bipy), 135.2 (s, bipy), 128.7 (s, bipy), 125.7 (s, bipy), 125.5 (s, bipy), 125.3 (s, Im), 125.3 (s, Im), 125.2 (s, bipy), 123.4 (s, Im), 123.3 (s, Im), 50.1 (s, bipy), 50.0 (s, ArCH_2_), 50.0 (s, ArCH_2_), 36.7 (s, ImCH_3_) ppm. ESI-MS *m*/*z* calcd for [C_50_H_52_F_24_N_14_P_4_Ru]^2+^, 765.1061; found, 765.1061. IR (ATR): ν_max_ 3169, 3125, 2349, 2326, 1604, 1577, 1566, 1467, 446, 1425, 1400, 1160, 760, 741, 731, 669, 648, 623, 516 cm^–1^.

#### Synthesis of [Ru(**L2**)_3_][PF_6_]_8_

A Schlenk flask was charged with [Ru(DMSO)_4_Cl_2_] (38.7 mg, 0.080 mmol, 1.0 equiv) and 3-([2,2′-bipyridin]-5-ylmethyl)-1-methyl-1*H*-imidazol-3-ium chloride (100 mg, 0.240 mmol, 3.0 equiv) under N_2_. Ethylene glycol (2 mL) was added, and the reaction was sparged with N_2_ for 15 min. The reaction was refluxed for 3 h after which the reaction was cooled and the product precipitated as an oil via dropwise addition to stirring THF (100 mL). The solution was decanted and the product coevaporated with MeOH (2 × 5 mL). The compound was purified by column chromatography (Al_2_O_3_; acetone/H_2_O; 100:0 → 50:50). The solvent was removed in vacuo and the oil redissolved in H_2_O (3 mL) and precipitated via the addition of NH_4_PF_6(aq)_ (0.1 M, 3 mL). The solid was filtered and dried in vacuo to give the title compound as an orange solid (54 mg, 0.023 mmol, 31%). ^1^H NMR spectrum (500 MHz, 293 K, CD_3_CN) 8.48 (d, ^3^*J*_HH_ = 8.5, 6H, bipyH), 8.43 (br s, 6H, ImH), 7.82 7.82 (dd, ^3^*J*_HH_ = 8.5, ^3^*J*_HH_ = 2.0, 6H, bipyH), 7.63 (d, ^3^*J*_HH_ = 2.0, 6H, bipyH), 7.39 (app. t, *J*_HH_ = 1.8, 7H, ImH), 7.20 (app. t, *J*_HH_ = 1.9, 6H, ImH), 5.25 (s, 12H, ArCH_2_), 3.84 (s, 18H, ImCH_3_); ^13^C{^1^H} NMR spectrum (126 MHz, 293 K, D_2_O; conducted on [Ru(L_2_)_3_]Cl_8_ due to solubility reasons); 156.2 (s, bipy), 151.1 (s, bipy), 137.2 (s, Im), 124.7 (s, bipy), 124.5 (s, Im), 122.6 (s, Im), 49.4 (s, ArCH_2_), 36.4 (s, ImCH_3_) ppm. ESI-MS *m*/*z* calcd for [C_60_H_64_N_18_RuP_6_F_36_]^2 +^, 1005.1314; found, 1005.1326. IR (ATR): ν_max_ 3169, 3126, 2349, 2324, 1664, 1578, 1564, 1477, 1446, 1425, 1400, 1167, 740, 671, 623, 470 cm^–1^.

## Results and Discussion

### Synthesis

**Scheme 1:**
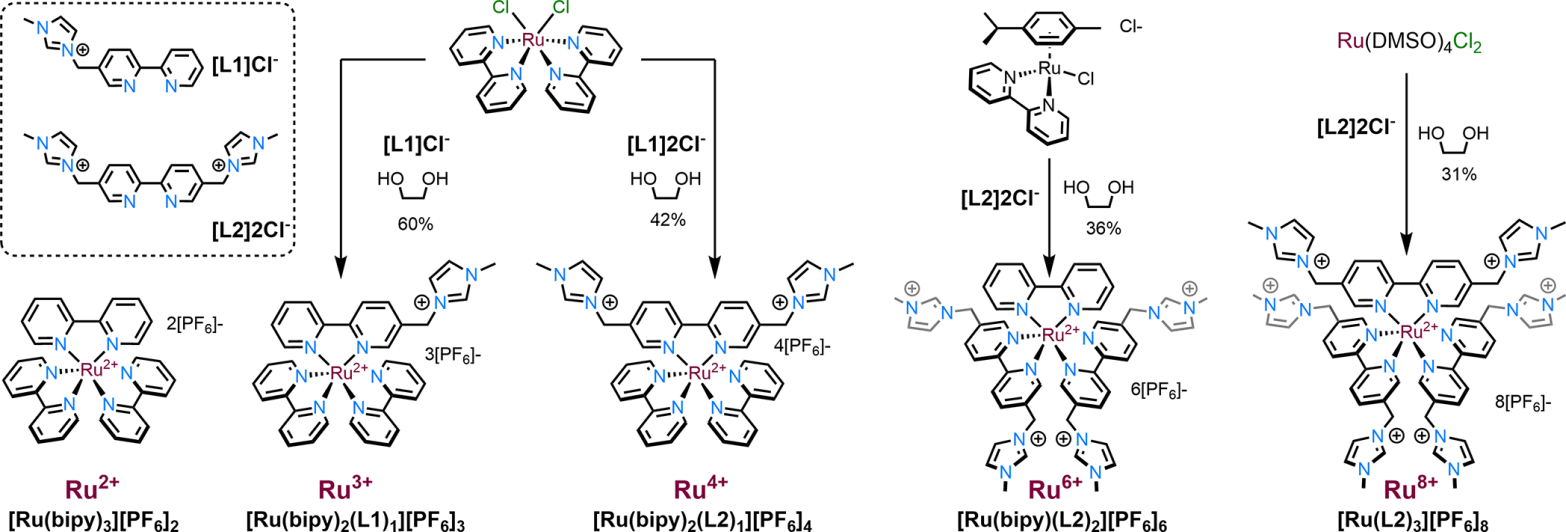
Synthetic Routes to the Family of Homologous Polycationic ruthenium(II) Bipyridine Complexes

The cationic bipy targets were chosen because they contained one or two imidazolium groups at the 5 and 5′ positions of the diimine. Therefore, the precursors 5-chloromethyl-2,2′-bipyridine and 5,5′-bis-chloromethyl-2,2′-bipyridine were synthesized according to previously reported procedures (via the carboxylic acid, ester, and methyl alcohol intermediates; Section S2.1).^41^ The final ligands were obtained from alkylation of the chloromethyl derivatives using *N*-methylimidazole as a reagent and solvent to give the target mono- and dicationic analogues, **L1** and **L2**,^42^ in 78% and >99% yield, respectively (Scheme 1). All experimental and characterization details are presented in the Supporting Information.

Despite the ubiquity of [Ru(bipy)_3_]^2+^ analogues and their syntheses in the literature,^43^ the application of relatively standard complexation procedures in our hands (viz. refluxing EtOH/H_2_O mixtures) did not give satisfactory outcomes with the cationic ligands **L1** and **L2**, resulting in incomplete complexation despite extended reaction times. This is presumably due to unfavorable electrostatic repulsion between the incoming ligands and the electropositive ruthenium center. Consequently, more forcing conditions were employed, for example, using refluxing ethylene glycol as a solvent. Application of this protocol to the formation of the tricationic and tetracationic complexes resulted in complete complexation within 3 h, with no evidence of decomposition.

**Scheme 2:**
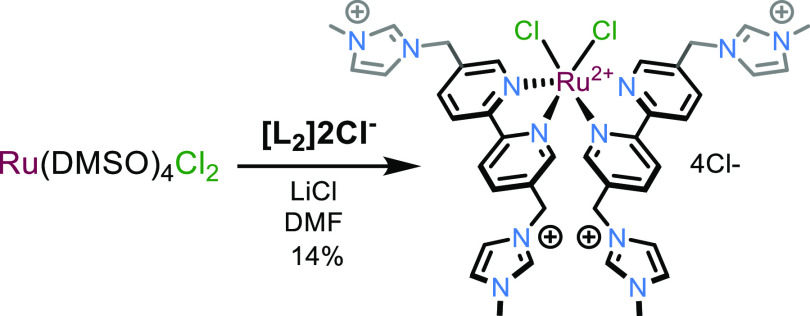
Attempted Synthesis of the Intermediate Species [Ru(**L2**)_2_Cl_2_]Cl_4_

After purification by column chromatography on SiO_2_ and anion exchange, [Ru(bipy)_2_(**L1**)][PF_6_]_3_, which has been previously reported and investigated in cell imaging studies,^44^ and [Ru(bipy)_2_(**L2**)][PF_6_]_4_ were afforded in yields of 60% and 40%, respectively. The synthesis of the hexacationic derivative [Ru(bipy)(**L2**)_2_][PF_6_]_6_ was attempted using a previously utilized two-step route via a [Ru(**L2**)_2_Cl_2_]Cl_4_ intermediate (Scheme 2). In this case, complexation was achieved using refluxing dimethylformamide (DMF) in the presence of excess LiCl to inhibit the formation of the tris-homoleptic [Ru(**L2**)_3_]^8+^ byproduct.^45^ After chromatographic purification, the target compound was isolated, albeit in low yield (14%), and the paucity of isolated compound precluded its use in the next synthetic step.

Therefore, an alternative procedure adapted from Meyer^25^ was employed to promote the formation of the desired compound. The reaction of [Ru(*p*-cymene)(bipy)Cl]Cl with ligand **L2** in refluxing ethylene glycol resulted in the total consumption of starting materials, but with the formation of several products (as indicated by ^1^H NMR spectroscopy), which was ascribed to ligand scrambling and thus related complexes of the general formula [Ru(bipy)_*x*_(**L2**)_3-x_]. Conversely, the reaction at 120 °C in ethylene glycol resulted in an incomplete reaction, even with extended reaction times. Gratifyingly, the reaction of [Ru(*p*-cymene)(bipy)Cl]Cl with ligand **L2** in refluxing 2-ethoxyethanol produced a single species after 48 h, with no evidence of ligand scrambling at ruthenium. After chromatography on silica and anion exchange, hexacationic [Ru(bipy)(**L2**)_2_][PF_6_]_6_ was isolated in 36% yield. The modest yields of the aforementioned complexes progressively decrease as a function of increasing charge which may be attributed to their elevated affinities to the stationary phase during chromatographic purification. Finally, the synthesis of [Ru(**L2**)_3_][PF_6_]_8_ was achieved using [Ru(DMSO)_4_Cl_2_] as a starting material and three equivalents of **L2** in refluxing ethylene glycol. In this case, the product was purified by column chromatography of the crude [Ru(**L2**)_3_]Cl_8_ using Al_2_O_3_—due to the high affinity of the octacationic homologue when utilizing silica—and anion exchanged thereafter to obtain [Ru(**L2**)_3_][PF_6_]_8_ in 31% yield. The isolation of this final complex matches the record for the most highly charged mononuclear Ru(II) complex reported in the literature.^25^

**Figure 1:**
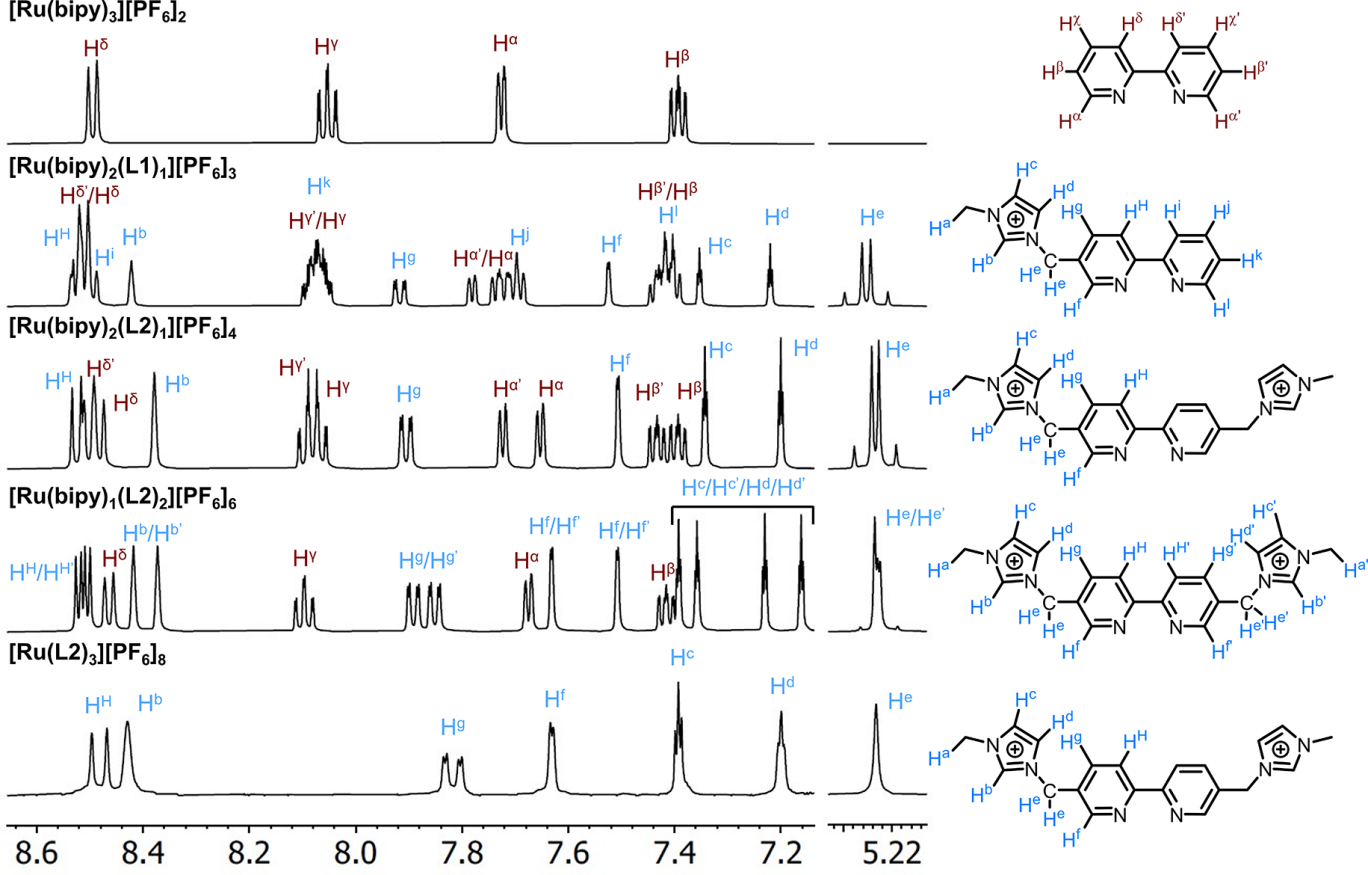
Assignment of ^1^H NMR spectra (500 MHz, 298 K, CD_3_CN) for the family of polycationic Ru(II) complexes.

The complexes were fully characterized using 1D and 2D NMR (298 K, CD_3_CN) spectroscopy (^1^H (Figure 1), ^13^C, COSY, HSQC, HMBC, ROESY) and mass spectrometry (Figures S17–S32). Complex [Ru(bipy)_2_(**L1**)][PF_6_]_3_ exhibits *C*_1_ symmetry, resulting in the inequivalence of all bipyridine ligands, giving a total of six discrete pyridine moieties. The tetra- and hexacationic complexes [Ru(bipy)_2_(**L2**)][PF_6_]_4_ and [Ru(bipy)(**L2**)_2_][PF_6_]_6_ both display time-averaged *C*_2_ symmetry in solution. In the case of the tetra-cationic derivative, the X′ notation denotes the pyridine fragment which is closest to the imidazolium-functionalized ligand as determined using through-space ^1^H–^1^H correlation. Unfortunately, the highly second-order spectrum of [Ru(bipy)(**L**_**2**_)_2_][PF_6_]_6_ prevented the absolute assignment of the unsymmetrical **L2** ligand. In the octacationic congener [Ru(**L2**)_3_][PF_6_]_8_, we observed a time-averaged *C*_3_ species, in agreement with the unfunctionalized parent [Ru(bipy)_3_][PF_6_]_2_. Other salient features of the ^1^H NMR spectra are the methylenic C*H*_2_ imidazolium resonances, which—in contrast to the uncoordinated ligands **L1** and **L2**—exhibited diasterotopicity at 298 K. This probably arises from the complexation and thus steric interference from proximate ligand systems, although subtle effects of counteranion ion-packing cannot be ruled out. Interestingly, in the case of [Ru(bipy)(**L2**)_2_][PF_6_]_6_, which displays inequivalence of the two halves of complexed **L2**, one C*H*_2_ resonance appears as a singlet corresponding to fast bond rotation on the NMR time scale, while the other is diastereotopic resulting from slow bond rotation. Due to the resolution of the 2D spectra (298 K, 600 MHz), it was not possible to identify each C*H*_2_ resonance.

### Single Crystal X-ray Crystallography

**Figure 2:**
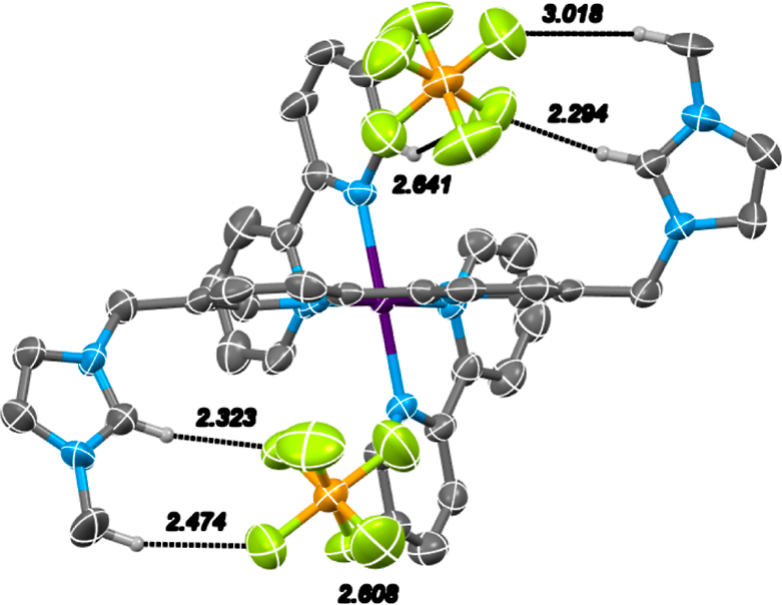
X-ray crystal structure of the [Ru(bipy)_2_(**L2**)][PF_6_]_4_ complex (ellipsoids are plotted at the 50% probability level; non-H-bonding hydrogen atoms and counterions omitted for clarity; CCDC no. 2250505).

Single crystals of [Ru(bipy)_2_(**L2**)][PF_6_]_4_ suitable for single-crystal X-ray diffraction (SCXRD) analysis (Figure 2, Table S1, Figures S33, 34) were grown from cooling of a concentrated KNO_3_/NH_4_PF_6_ aqueous solution. The compound crystallized in the triclinic *P*1̅ space group with two molecules and the eight hexafluorophosphate anions in the asymmetric unit with no additional solvent present. The three-dimensional network is perpetuated by intermolecular C–H··F hydrogen bonding interactions between the fluorine atoms of the counteranions and polarized C–H bonds in the complex. Mirroring the solution-state behavior, complex [Ru(bipy)_2_(**L2**)][PF_6_]_4_ displays pseudo-*C*_2_ symmetry with an *anti*-arrangement of the flanking methyl-imidazolium groups which hydrogen-bonds intramolecularly to two nested PF_6_^–^ anions, with mean C···F distances of 3.191 Å to the most acidic imidazolium N–CH–N hydrogen. This is augmented by supplementary elongated interactions with the imidazolium CH_3_ groups ((C···F)_mean_ = 3.617 Å) and adjacent unfunctionalized bipyridine ligands ((C···F)_mean_ = 3.403 Å). The coordination geometry is largely unchanged from simple heteroleptic ruthenium diimine analogues, with mean Ru–N_bipy_ distances of 2.071 Å and Ru–N_L2_ distances of 2.066 Å (cf. [Ru(bipy)_2_(bipicoline)][PF_6_]_2_; (Ru–N_bipy_)_mean_ = 2.065 Å, (Ru–N_bipicoline_)_mean_ = 2.067 Å),^19^ indicating that the introduction of the charged imidazolium groups does not significantly alter the ligand field strength of the resultant complex.

### Electrochemistry

The electrochemical properties of the family of polycationic ruthenium complexes were explored using cyclic voltammetry in MeCN solution at 1 mM concentration, using [^n^Bu_4_N][PF_6_] as the supporting electrolyte (0.1 M) and the Fc/Fc^+^ redox couple as a reference (Table 1). All complexes exhibit quasi-reversible or reversible redox waves for both the oxidation and reduction potentials of the metal center and ligand sphere, respectively (Figure S35). The principal feature of the Ru^II^/Ru^III^ couple is the small variance of the tri- and tetracationic oxidation potentials (*E*^0^) ([Ru(bipy)_2_(**L1**)][PF_6_]_3_ = +0.88 V, [Ru(bipy)_2_(**L2**)][PF_6_]_4_ = 0.91 V) compared to the unsubstituted parent [Ru(bipy)_3_][PF_6_]_2_ (+0.89 V), indicating only a small effect of the increase of peripheral charge in the second coordination sphere. For the more highly charged analogues, an increased perturbation is observed, manifested by an increasingly facile one-electron reduction of the Ru^II^ center. For the hexacationic [Ru(bipy)(**L2**)_2_][PF_6_]_6_ complex, *E*^0^ shifts to +0.83 V, while for the octacationic derivative [Ru(**L2**)_3_][PF_6_]_8_, *E*^0^ = +0.80 V. Despite the increased facility of this oxidation process, qualitative observation of the voltammograms indicate that this process became less reversible traversing down the series, which can be reconciled by the decreased stability of the generated Ru^III^ species in solution in close proximity to the multiple pendant cationic imidazolium moieties. Three distinct negative potentials were observed in all cases corresponding to bipyridine reduction of the tris-homoleptic and tris-heteroleptic complexes; imidazolium groups are known to be electrochemically inert within the redox potential window studied.^20^ Once again, the reduction waves of [Ru(bipy)_2_(**L1**)][PF_6_]_3_ and [Ru(bipy)_2_(**L2**)][PF_6_]_4_ largely resemble unsubstituted [Ru(bipy)_3_][PF_6_]_2_, while we observed the ligand reductions becoming more difficult in the highly charged [Ru(bipy)(**L2**)_2_][PF_6_]_6_ and [Ru(**L**_**2**_)_3_][PF_6_]_8_ complexes. The ease of reduction (cf. [Ru(bipy)_3_][PF_6_]_2_) correlates with the overall charge state of the [Ru(bipy)(**L2**)_2_][PF_6_]_6_ and [Ru(**L2**)_3_][PF_6_]_8_ complexes (Δ_mean_ = −0.14 and Δ_mean_ = −0.21, respectively). The observation of more difficult ligand reduction potentials is consistent with the more facile Ru^II^/Ru^III^ redox couples, although this is counterintuitive since one might expect the inverse upon introducing multiple positive charges close to the metal center. We tentatively ascribe this behavior to the presence of the multiple ion-paired hexafluorophosphate anions, which may stabilize the formation of Ru^III^ and thus destabilize the reduction of the bipyridine ligands. Indeed, the magnitude of the shift of the redox process is larger for the ligand reduction potentials and can be rationalized in the relative distance from the charged imidazolium groups (vide supra; SCXRD). Ligand reduction, and its proximity to the imidazolium moieties, is more susceptible to inductive charged effects from the hexafluorophosphate anions, which are ion-paired with the imidazolium groups, when compared to the Ru^II^/Ru^III^ oxidation.

**Table 1:** Electrochemical Oxidation and Reduction Potentials of the Family of Complexes

complex	RuII/III	bipy^0/–1^	bipy^–1/–2^	bipy^–2/–3^
[Ru(bipy)_3_][PF_6_]_2_	0.89(200)	–1.76(161)	–1.94(180)	–2.18(200)
[Ru(bipy)_2_(**L1**)][PF_6_]_3_	0.88(120)	–1.75(100)	–1.94(113)	–2.20(115)
[Ru(bipy)_2_(**L2**)][PF_6_]_4_	0.91(120)	–1.71(130)	–1.93(100)	–2.21(130)
[Ru(bipy)(**L2**)_2_][PF_6_]_6_	0.83(160)	–1.83(140)	–2.08(220)	–2.34(203)
[Ru(**L2**)_3_][PF_6_]_8_	0.80(209)	–1.95(130)	–2.15(190)	–2.41(187)

### Photophysical Properties of the Complexes

**Figure 3:**
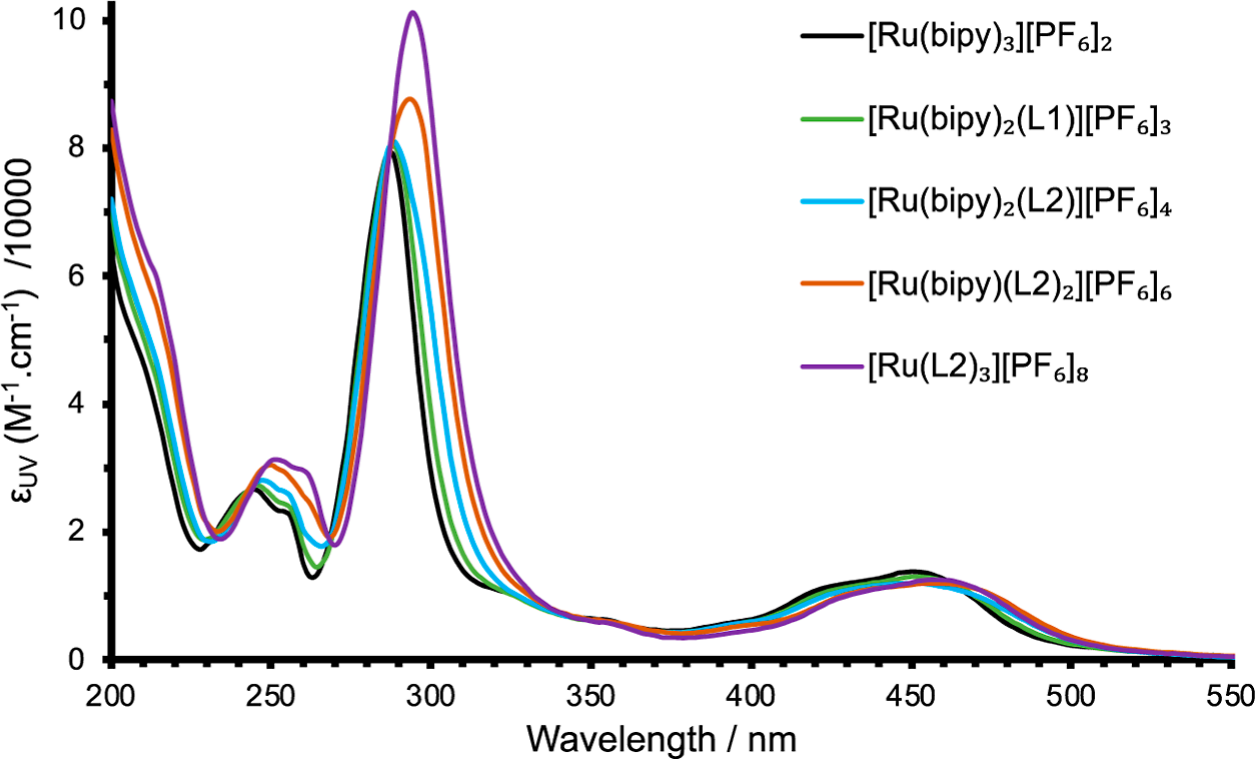
UV–vis absorption spectra for the family of polycationic ruthenium complexes (MeCN).

The UV–vis absorption data for all complexes were obtained in aerated acetonitrile and are presented in Table 2 and Figure 3. The two notable regions are high-intensity UV absorptions corresponding to allowed π → π* transitions and weaker absorptions in the visible region, around 450 nm, due to spin-allowed ^1^MLCT absorptions. The tri- and tetracationic complexes displayed high energy absorptions at 288 nm (ε ≈ 82,000 M^–1^ cm^–1^), which are comparable to that of [Ru(bipy)_3_][PF_6_]_2_. For the hexacationic complex, this feature is slightly shifted to lower energy (294 nm), but with an increased molar absorptivity (88,000 M^–1^ cm^–1^), while the octacationic derivative displays the same maximum but again with a concomitantly higher absorption coefficient (101,300 M^–1^ cm^–1^). The ^1^MLCT transition displayed only a minor variation in the wavelength position (450–456 nm), with the associated molar absorptivity slightly decreasing down the series of complexes. The shape of the MLCT band envelope is consistent with previous descriptions that describe the vibronic progressions associated with the transition.^46^ These results demonstrate that the pendant imidazolium groups induce a small effect on the ground state absorption properties of the Ru(II) complexes.

**Table 2:** Absorption and Photoluminescence Data for the Family of Polycationic Complexes^a^,^b^

complex	λ_MLCT_/nm (ε/M^–^^1^ cm^–^^1^)	λ_em_/nm^c^	τ/ns^d^	Degas τ/ns^d^	Φ_Ru_	Degas Φ_Ru_	***k***_r_/10^4^ s^–^^1^	***k***_nr_/10^5^ s^–^^1^
[Ru(bipy)_3_][PF_6_]_2_	450 (15,300)	610	157	867	0.018^47^	0.095^48^		
[Ru(bipy)_2_(**L1**)][PF_6_]_3_	450 (14,000)	633	218	907	0.017^e^	0.069^f^	0.76	10.3
[Ru(bipy)_2_(**L2**)][PF_6_]_4_	452 (13,100)	654	236	568	0.014^e^	0.049^f^	0.86	16.4
[Ru(bipy)(**L2**)_2_][PF_6_]_6_	456 (12,300)	643	331	744	0.018^e^	0.041^f^	0.55	12.9
[Ru(**L2**)_3_][PF_6_]_8_	456 (12,500)	632	362	841	0.021^e^	0.054^f^	0.64	11.2

a All measurements were obtained in MeCN at 293 K.

b 1 × 10^–5^ M.

c λ_ex_ = 450 nm.

d Observed lifetime, λ_ex_ = 295 nm.

e Using [Ru(bipy)_3_][PF_6_]_2_ in aerated MeCN as a reference (Φ = 0.018).^48^

f Using [Ru(bipy)_3_][PF_6_]_2_ in degassed MeCN as a reference (Φ = 0.095), errors are estimated at 15%.^48^ Estimates of *k*_r_ and *k*_nr_ using *k*_r_ = Φ/τ and *k*_nr_ = (1 – Φ)/τ.

**Figure 4:**
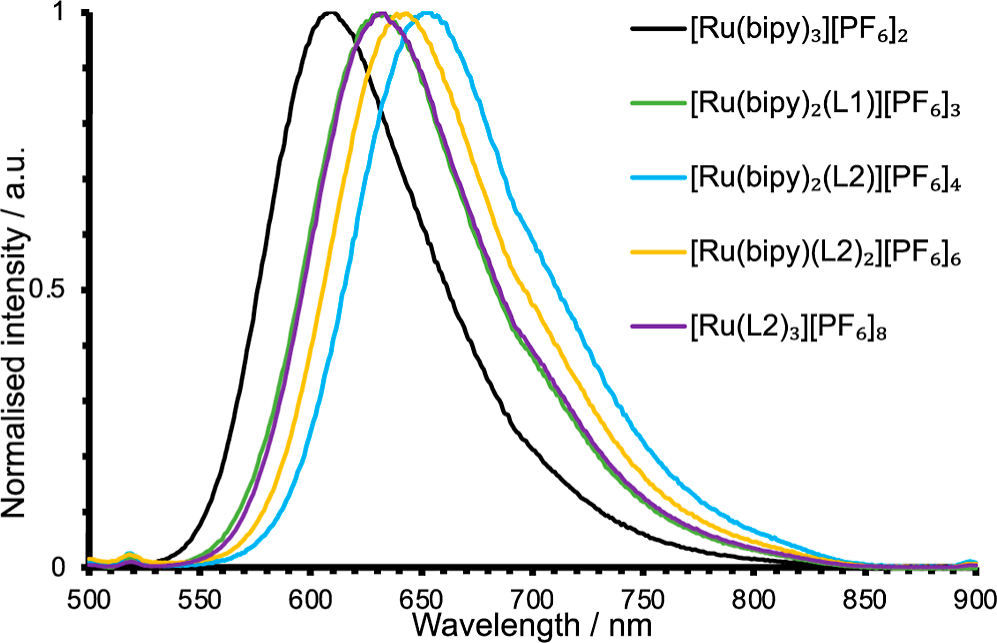
Normalized emission spectra for the family of polycationic ruthenium complexes (293 K, aerated MeCN, 10^–5^ M).

The corresponding photoluminescence spectra of the complexes were initially recorded in aerated MeCN. All compounds were found to be luminescent upon excitation at 450 nm (i.e., directly into the ^1^MLCT absorption band), showing a broad featureless emission maxima at ca. 630 nm which clearly corresponds to ^3^MLCT emission. The effect of the imidazolium groups upon the excited state characteristics was found to be more pronounced than for ground state absorption (Figure 4). Introduction of one imidazolium group (i.e., [Ru(bipy)_2_(**L1**)][PF_6_]_3_) induced a bathochromic shift of 23 nm vs unfunctionalized [Ru(bipy)_3_][PF_6_]_2_. This was also observed for the tetracationic [Ru(bipy)_2_(**L2**)][PF_6_]_3_ complex (λ_em_ = 654 nm, Δλ_MLCT_ = 44 nm), with the effect of the second imidazolium group being roughly additive. Strikingly, the hexacationic complex did not continue this trend (λ_em_ = 643 nm, Δλ_MLCT_ = 33 nm), displaying a relative hypsochromic shift vs the tetracationic complex. Finally, the emission maximum of [Ru(**L2**)_3_][PF_6_]_8_ (λ_em_ = 632 nm, Δλ_MLCT_ = 21 nm) again shifted to higher energy.

In addition to the acquisition of experimental data, supporting density functional theory (DFT) results showed that all of the transitions observed within the range of 250 nm < λ < 700 nm are predicted to be strongly MLCT in character (the calculated Kohn–Sham molecular orbitals are shown in Tables S2–S5). In all cases, the transitions involved in the absorption bands observed within this window arise from occupied orbitals with 70% or greater Ru(4d) character, and with unoccupied character <10% Ru(4d). TD-DFT calculations show a qualitative agreement with the luminescence band positions, but they appear to greatly overpredict the wavelength shifts in the absorption band positions (Tables S6–S10). However, the longest wavelength absorber/emitter is correctly predicted to be the [Ru(bipy)_2_(**L2**)]^4+^ complex, seemingly due to a relative reduction in the energy of the LUMO.

**Figure 5:**
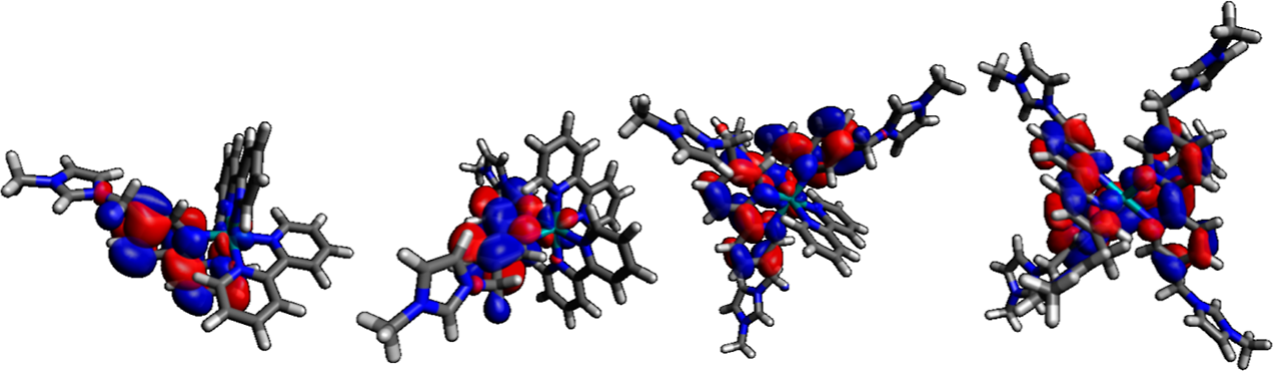
Calculated Kohn–Sham LUMOs for (left to right) [Ru(bipy)_2_(**L1**)]^3+^, [Ru(bipy)_2_(**L2**)]^4+^, [Ru(bipy)(**L2**)_2_]^6+^, and [Ru(**L2**)_3_]^6+^.

In each complex, the strongest transitions are predicted where there are significant HOMO–2/LUMO contributions, and therefore, the influence of the position of the LUMO is probably of great importance. For [Ru(bipy)_2_(**L1**)]^3+^ and [Ru(bipy)_2_(**L2**)]^4+^, the calculations predict that the LUMO is primarily localized on **L1**/**L2**. This is not surprising as this affords a more nonzero transition probability based on orbital overlap arguments, with low-lying **L1**/**L2**-centered unoccupied orbitals, particularly given the high localization of many of the occupied orbitals on the metal center. For complexes [Ru(bipy)(**L2**)_2_]^6+^ and [Ru(**L2**)_3_]^8+^, the LUMOs may become delocalized across multiple **L2** ligands (Figure 5), perhaps explaining the trend noted in the experimental data.

Time-resolved photophysical measurements yielded the emission lifetimes, which increased down the series from 218 to 362 ns (cf. 157 ns for [Ru(bipy)_3_][PF_6_]_2_) in aerated MeCN, possibly indicating that quenching in the oxygenated solvent may be inhibited by increasing the number of imidazolium moieties. Quantum yield (QY) measurements were also conducted in aerated MeCN and also showed a break in behavior demonstrated by the λ_em_ data: in general, the quantum yield reduced up to the tetracationic complex (Φ = 0.014) and thereafter recovered down the series to eventually surpass unfunctionalized [Ru(bipy)_3_][PF_6_]_2_ in the case of [Ru(**L2**)_3_][PF_6_]_8_ (Φ = 0.021). The spectroscopic measurements were repeated in deoxygenated MeCN. First, the lifetimes were increased in all cases emphasizing the ^3^MLCT nature of the phosphorescence. The degassed lifetime and QY data now broadly replicate the same trend: a general reduction in values to [Ru(bipy)_2_(**L2**)][PF_6_]_4_ and then a subsequent increase to [Ru(**L2**)_3_][PF_6_]_8_. To probe this further, the relative rates of radiative and nonradiative decay were calculated and show that the values of the nonradiative decay constant (*k*_nr_) correlate with the Energy Gap Law. Thus, the longest wavelength tetracationic emitter has the highest value of *k*_nr_.

Finally, we were interested in the potential utility of these polycationic Ru(II) complexes in further optoelectronic applications. Our hypothesis was that the increase in positive charge could be exploited in the electrostatic assembly of photoactive ion pairs. In particular, we hypothesized that the use of an anionic lanthanide complex might permit energy transfer from a Ru(II)-based donor. Therefore, polyanionic [Yb(dpa)_3_]^3–^ (where dpa = pyridine-2,6-dicarboxylic acid) was identified as an ideal candidate. Initially, ^1^H NMR studies were undertaken whereby [Yb(dpa)_3_]^3–^ was added to a solution of [Ru(bipy)_2_(**L2**)]^4+^ in 0.1 M KNO_3_ D_2_O solution (Figure S36). Addition of the paramagnetic Yb(III) complex induced small perturbations (Δδ < 0.2 ppm) of the bipyridine resonances of [Ru(bipy)_2_(**L2**)]^4+^, although their broad nature precludes absolute assignment. More strikingly, the peripheral methylenic and methyl resonances exhibit broadening and downfield shifts of 1.09 and 0.68 ppm, respectively, indicating interactions between the two complex units.

**Figure 6:**
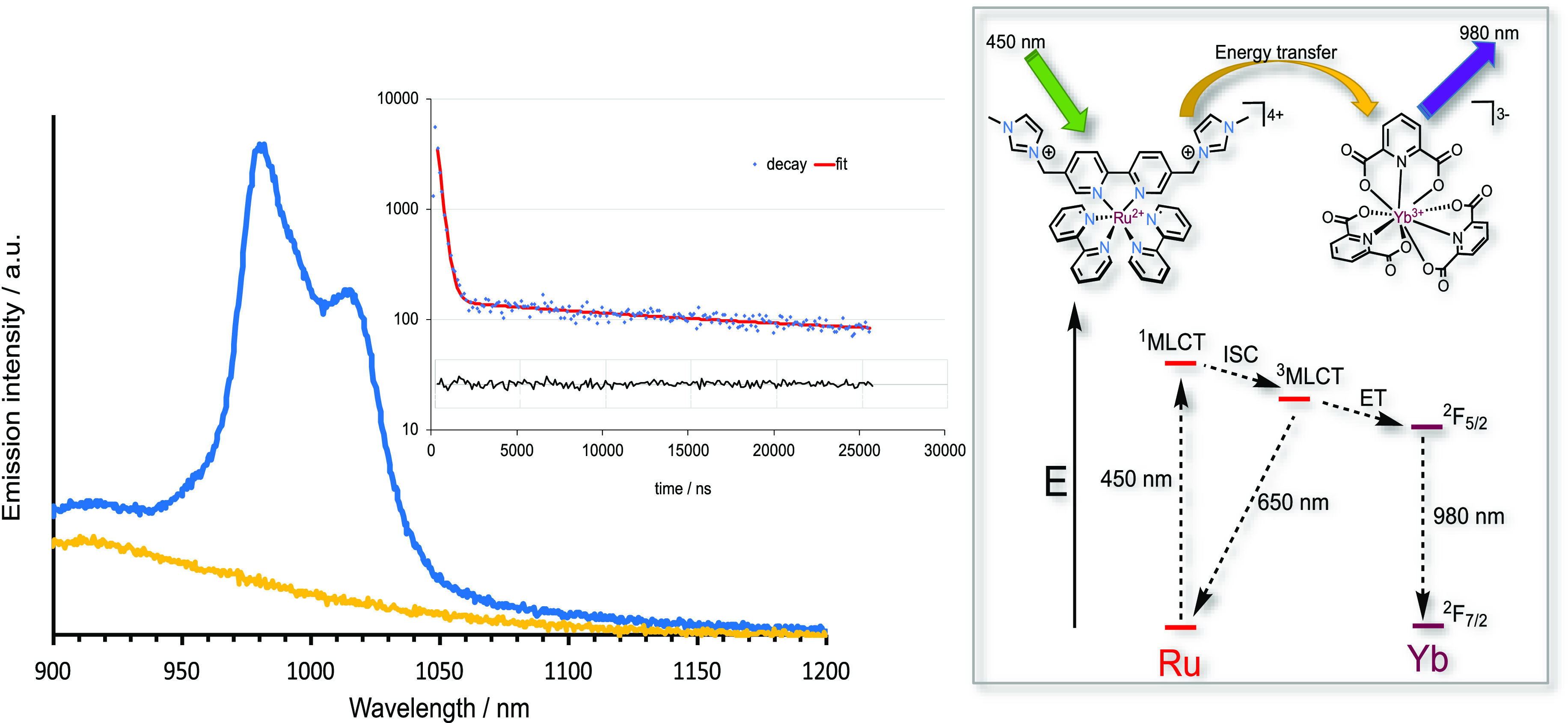
Comparison of the NIR emission spectra of [Ru(bipy)_3_]^2+^ (orange) and [Ru(bipy)_2_(**L2**)]^4+^ (blue) solutions in the presence of 2 eq [Yb(dpa)_3_]^3–^ (293 K, aerated D_2_O, 10^–5^ M Ru(II) complex, λ_ex_ = 450 nm). Fitted decay (residual errors shown) for [Ru(bipy)_2_(**L2**)]^4+^ in the presence of 2 eq [Yb(dpa)_3_]^3–^ (293 K, aerated D_2_O, 10^–5^ M, λ_ex_ = 355 nm, λ_em_ = 980 nm). The obtained lifetimes were 0.3 μs (31% relative weighting) and 21.9 μs (69%). The proposed photophysical pathways are shown inset.

Photoluminescent studies were then undertaken to probe the photophysical attributes of the solution mixture. First, an aerated D_2_O solution of the parent unfunctionalized [Ru(bipy)_3_]^2+^ was measured in the presence of 2 eq [Yb(dpa)_3_]^3–^. An excitation wavelength of 450 nm was used and is selective for the ^1^MLCT band of the Ru(II) complex (note that [Yb(dpa)_3_]^3–^ does not absorb >320 nm). No characteristic signal for Yb(III)-based emission was noted, with only the weak tail of the ^3^MLCT band evident in the NIR window. We repeated the experiment using [Ru(bipy)_2_(**L2**)]^4+^ which was chosen due to its high charge and also its significantly red-shifted ^3^MLCT emission wavelength when compared to [Ru(bipy)_3_]^2+^ (654 nm vs 610 nm) which should maximize spectral overlap with Yb(III). Utilizing the more highly cationic [Ru(bipy)_2_(**L2**)]^4+^ produced a remarkably different result: the characteristic emission profile of Yb(III) was immediately evident with a strong structured emission ca. 980 nm with a shoulder at 1020 nm corresponding to the Stark-split sublevels of the ^2^F_5/2_ → ^2^F_7/2_ transition (Figure 6). From the baseline profile, it is clear that the Yb(III) emission is superimposed upon the low energy tail of the ^3^MLCT band which was corroborated through time-resolved NIR measurements (λ_em_ = 980 nm) that revealed a decay profile (Figure 6) that fitted well to a biexponential function giving two distinct lifetime values of 0.3 (further refined to 392 ns using a shorter 1 μs window for the analysis) and 21.9 μs. Since [Yb(dpa)_3_]^3–^ has a reported lifetime of 39 μs in D_2_O,^48^ the measured values are attributed to the coemission of the residual ^3^MLCT signal and the Yb-centered emission, respectively. Unfortunately, the biexponential character of the decay at 980 nm prevented us from extracting a rise time to the Yb(III) emission. Therefore, these measurements establish that Ru → Yb energy transfer, which we assume proceeds via a Förster mechanism, must be feasible in electrostatically assembled mixtures of ion-paired complexes in solution. Given that this behavior was not observed for [Ru(bipy)_3_]^2+^, the presence of additional charge and/or pendant hydrogen-bonding interactions may be vital in the observation of Ru → Yb energy transfer.

## Conclusions

This study has shown that a variety of synthetic approaches can be employed to isolate a series of cationic Ru(II) polypyridine-type complexes with overall charges systematically varying from +2 to +8; the octa-cationic complex match the highest reported magnitude of charge in the literature for Ru(II) species. Each of the complexes was demonstrated to be luminescent from a ^3^MLCT emitting state (610–654 nm). The interesting trend in λ_em_ shows a maximum value for the +4 species before hypsochromically shifting for +6 and +8, which may indicate the shielding effects of ion pairing in solution; degassed lifetime and quantum yield measurements support this trend. The solution state photophysical utility of polycationic Ru(II) complexes was exploited in experiments using a polyanionic Yb(III) complex. These studies show that energy transfer and subsequent sensitization of the Yb(III) excited state are possible in free solution resulting in NIR emission from Yb(III). This implies that electrostatically driven ion pairing (perhaps supported by H-bonding interactions) is possible in solution and can be used to facilitate photophysical phenomena, such as through space energy transfer mechanisms. Further detailed photophysical studies are required to expand the range of measurements using different combinations of polycationic Ru(II) donors and [Ln(dpa)_3_]^3–^ acceptors. Such studies should allow for a more detailed appreciation of the importance of charge and the efficiencies of energy transfer in sensitized pairings.

More broadly, the development of mixed ligand complexes of Ru(II) and the study of localized versus delocalized excited states may have importance in areas of study focused on ultrafast electron injection at semiconductor interfaces as noted elsewhere. An investigation of interligand electron transfer (electron hopping) within excited state polycationic Ru(II) species could be particularly relevant.

## Supporting Information Available

^1^H and ^13^C NMR, and ESI-HRMS spectra for ligands and corresponding complexes; single-crystal X-ray crystallography; cyclic voltammetry; and density functional theory (PDF)

## Accession Codes

CCDC 2250505 contains the supplementary crystallographic data for this paper. These data can be obtained free of charge via www.ccdc.cam.ac.uk/data_request/cif, or by emailing data_request@ccdc.cam.ac.uk, or by contacting The Cambridge Crystallographic Data Centre, 12 Union Road, Cambridge CB2 1EZ, UK; fax: +44 1223 336033.
