# Supplementary material for: Polycationic Ru(II) Luminophores: Syntheses, Photophysics, and Application in Electrostatically Driven Sensitization of Lanthanide Luminescence
Source: Inorg Chem. 2023 Nov 20;62(48):19446–56. doi: 10.1021/acs.inorgchem.3c02352 (PMC10698718; doi:10.1021/acs.inorgchem.3c02352)
Supplement: Supplementary file 1 — ic3c02352_si_001.pdf [file ic3c02352_si_001.pdf]

Supporting Information

Polycationic Ru(II) Lumophores: Syntheses, Photophysics and Application in Electrostatically Driven Sensitization of Lanthanide Luminescence

Richard C. Knighton,[a*] Joseph M. Beames[b] and Simon J. A. Pope[a*]

[a]School of Chemistry, Main Building, Cardiff University, Cardiff CF10 3AT, Cymru/Wales;

[b]School of Chemistry, University of Birmingham, Birmingham B152TT.

Email: R.C.Knighton@soton.ac.uk; popesj@cardiff.ac.uk

Contents

S1. Experimental procedures and spectra 2
S1.1 Ligand Syntheses and spectra 2
S1.1.1 Synthesis of 1-(2-oxo-2-(pyridin-2-yl)ethyl)pyridin-1-ium iodide 2
S1.1.2 Synthesis of 5-methyl-2,2'-bipyridine 3
Figure S2. *^{1}H NMR spectrum (300 MHz, 293 K, $CDCl_3$)* 4
S1.1.3 Synthesis of [2,2'-bipyridine]-5-carboxylic acid 4
Figure S3. *^{1}H NMR spectrum (300 MHz, 293 K, $(CD_3)_2SO$)* 5
S1.1.4 Synthesis of ethyl [2,2'-bipyridine]-5-carboxylate 5
Figure S4. *^{1}H NMR spectrum (300 MHz, 293 K, $CDCl_3$)* 6
S1.1.5 Synthesis of [2,2'-bipyridin]-5-ylmethanol 6
Figure S5. *^{1}H NMR spectrum (300 MHz, 293 K, $CDCl_3$)* 7
S1.1.6 Synthesis of 5-(chloromethyl)-2,2'-bipyridine 7
Figure S6. *^{1}H NMR spectrum (300 MHz, 293 K, $CDCl_3$)* 8
S1.1.7 Synthesis of [2,2'-bipyridine]-5,5'-dicarboxylic acid 8
Figure S7. *^{1}H NMR spectrum (300 MHz, 293 K, $(CD_3)_2SO$)* 8
S1.1.8 Synthesis of diethyl [2,2'-bipyridine]-5,5'-dicarboxylate 9
Figure S8. *^{1}H NMR spectrum (300 MHz, 293 K, $CDCl_3$)* 9
S1.1.9 Synthesis of [2,2'-bipyridine]-5,5'-diyldimethanol 9
Figure S9. *^{1}H NMR spectrum (500 MHz, 293 K, $CDCl_3$)* 10
S1.1.10 Synthesis of 5,5'-bis(chloromethyl)-2,2'-bipyridine 10
Figure S10. ^{1}H NMR spectrum (300 MHz, 293 K, $CDCl_3$) 10
S1.1.11 Synthesis of 3-([2,2'-bipyridin]-5-ylmethyl)-1-methyl-1H-imidazol-3-ium chloride 11
Figure S11. *^{1}H NMR spectrum (500 MHz, 293 K, CD_3OD)* 11
Figure S12. *^{13}C NMR spectrum (126 MHz, 293 K, CD_3OD)* 11
Figure S13 *.ESI-MS spectrum* 12
S1.1.12 Synthesis of 3,3'-([2,2'-bipyridine]-5,5'-diylbis(methylene))bis(1-methyl-1H-imidazol-3-ium) dichloride 12
Figure S14. *^{1}H NMR spectrum (500 MHz, 293 K, CD_3OD)* 12
Figure S15. *^{13}C NMR spectrum (126 MHz, 293 K, CD_3OD)* 13
Figure S16. *ESI-MS spectrum* 13
S1.2 Complex Spectra 14
S1.2.1 Synthesis of $[Ru(bipy)_2(L1)_1][PF_6]_3$ 14
Figure S17. *^{1}H NMR spectrum (500 MHz, 293 K, CD_3CN)* 14
Figure S18. *^{13}C NMR spectrum (126 MHz, 293 K, CD_3CN)* 14
Figure S19. *ESI-MS spectrum* 15
S1.2.2 Synthesis of $[Ru(bipy)_2(L2)_1][PF_6]_4$ 15
Figure S20. *^{1}H NMR spectrum (500 MHz, 293 K, CD_3CN)* 15
Figure S21. *^{13}C NMR spectrum (126 MHz, 293 K, CD_3CN)* 16
Figure S22. *ESI-MS spectrum* 16
S1.2.3 Synthesis of $[Ru(L_2)Cl_2]Cl_4$ 17
Figure S23. *^{1}H NMR spectrum (300 MHz, 293 K, CD_3OD)* 17
Figure S24. *^{13}C NMR spectrum (126 MHz, 293 K, CD_3OD)* 17
Figure S25. *ESI-MS spectrum* 18
S1.2.4 Synthesis of [Ru)(*p*-cymene)(bipyCl]Cl 18
Figure S26. *^{1}H NMR spectrum (500 MHz, 293 K, CD_3CN)* 19
S1.2.5 Synthesis of $[Ru(bipy)_1(L2)_2][PF_6]_6$ 19
Figure S27. *^{1}H NMR spectrum (500 MHz, 293 K, CD_3CN)* 19
Figure S28. *^{13}C NMR spectrum (126 MHz, 293 K, CD_3CN)* 20

Figure S29. ESI-MS spectrum 20
S1.2.6 Synthesis of [Ru(L2)$_3$][PF$_6$]$_8$ **20**
Figure S30. ^{1}H NMR spectrum (500 MHz, 293 K, CD_3CN) 21
Figure S31. ^{13}C NMR spectrum (126 MHz, 293 K, D_2O) 21
Figure S32. ESI-MS spectrum 22
S2. Single-crystal X-ray crystallography **23**
S2.1 Structure of [Ru(bipy)$_2$(L2)][PF$_6$]$_4$ **23**
Table S1. Crystal data and structure refinement for **[Ru(bipy)$_2$(L2)][PF$_6$]$_4$**. 23
Figure S33. Single crystal X-ray structure of **[Ru(bipy)$_2$(L2)][PF$_6$]$_4$** (ellipsoids plotted at the 50% probability level) 24
Figure S34. Single crystal X-ray structure of **[Ru(bipy)$_2$(L2)][PF$_6$]$_4$** (ellipsoids plotted at the 50% probability level; H-atoms and counteranions omitted for clarity) 24
S3 Cyclic Voltammetry **25**
Figure S35. Cyclic voltammograms for the family of ruthenium complexes 25
S4 Density Functional Theory **26**
Table S2. Calculated Kohn-Sham molecular orbitals for [Ru(bipy)$_2$(**L1**)]$^{3+}$. 26
Table S3. Calculated Kohn-Sham molecular orbitals for [Ru(bipy)$_2$(**L2**)]$^{4+}$. 26
Table S4. Calculated Kohn-Sham molecular orbitals for [Ru(bipy)(**L2**)$_2$]$^{6+}$. 27
Table S5. Calculated Kohn-Sham molecular orbitals for [Ru(**L2**)$_3$]$^{8+}$. 27
Table S6. A description of the calculated MO contributions, excited state descriptions and their associated transitions for [Ru(bipy)$_2$(**L1**)]$^{3+}$ where Bipy1 and Bipy2 are the two distinct bipyridine ligands. 28
Table S7. A description of the calculated MO contributions, excited state descriptions and their associated transitions for [Ru(bipy)$_2$(**L2**)]$^{4+}$ where Bipy1 and Bipy2 are the two distinct bipyridine ligands. 29
Table S8. A description of the calculated MO contributions, excited state descriptions and their associated transitions for [Ru(bipy)(**L2**)$_2$]$^{6+}$ (L2$_1$ and L2$_2$ are the two distinct modified ligands; bipy = bipyridine). 30
Table S9. A description of the calculated MO contributions, excited state descriptions and their associated transitions for [Ru(**L2**)$_3$]$^{8+}$ (L2$_1$, L2$_2$, and L3$_3$ are the three distinct modified ligands). 31
Table S10 Computed values for the $S_0 \rightarrow T_1$ energy gap from vertical TD-DFT calculations. 32
S5 Ion pairing effects **33**
Figure S36. ^{1}H NMR (300 MHz, 293 K, 0.1M KNO_3 D_2O) spectra of [Ru(bipy)$_2$(**L2**)][PF$_6$]$_4$ (top) and a 1:1 mixture of [Ru(bipy)$_2$(**L2**)][PF$_6$]$_4$ and Na$_3$[Yb(dpa)$_3$] 33
References **34**

S1. Experimental procedures and spectra

S1.1 Ligand Syntheses and spectra

S1.1.1 Synthesis of 1-(2-oxo-2-(pyridin-2-yl)ethyl)pyridin-1-ium iodide

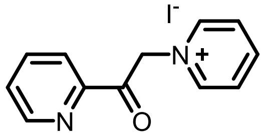

This compound was synthesised according to an adapted literature procedure.[1]

2-acetylpyridine (5.6 mL, 50.0 mmol, 1.0 eq.) and I_2 (14.2 g, 56.0 mmol, 1.12 eq.) were dissolved in pyridine (60 mL) and refluxed for six hours. The reaction was cooled to ambient temperature, filtered, and washed with Et_2O (2 × 50 mL). The compound was suspended in $CHCl_3$ (400 mL) and filtered. The crude material was recrystallised from hot EtOH (*ca*. 200 mL) to obtain the title compound as a black solid (6.58 g, 20.2 mmol, 40%). ^{1}H NMR (300 MHz, 293 K, $(CD_3)_2SO$) 9.07 – 8.95 (m, 2H, py$^+$H), 8.88 (dt, $^3J_{HH}$ = 4.8, $^4J_{HH}$ = 1.3, 1H, pyH), 8.73 (tt, $^3J_{HH}$ = 7.8, $^3J_{HH}$ = 1.4, 1H, py$^+$H), 8.32 – 8.23 (m, 2H, py$^+$H), 8.19 – 8.04 (m, 2H, pyH), 7.84 (ddd, $^3J_{HH}$ = 7.4, 4.7, $^4J_{HH}$ = 1.5, 1H), 6.50 (s, 2H, Py$^+$CH$_2$).

Figure S1. ^{1}H NMR spectrum (300 MHz, 293 K, $(CD_3)_2SO$)

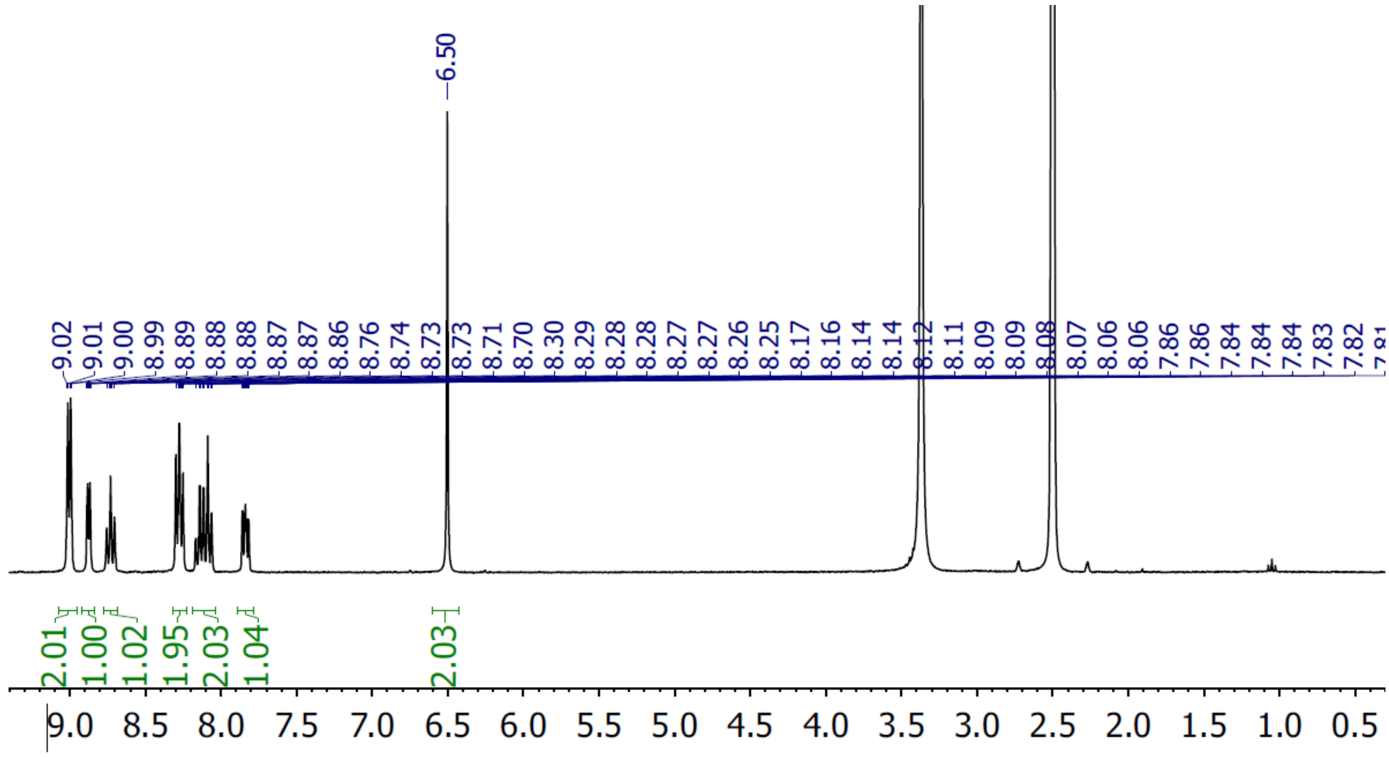

S1.1.2 Synthesis of 5-methyl-2,2'-bipyridine

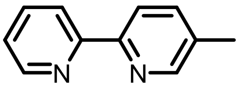

This compound was synthesised as previously described.[2]

1-(2-oxo-2-(pyridin-2-yl)ethyl)pyridin-1-ium iodide (14.4 g, 43.5 mmol, 1.0 eq.), methacrolein (3.59 mL, 43.5, 1.0 eq.) and NH_4OAc (16.8 g, 217 mmol, 5.0 eq.) were dissolved in formamide (130 mL) under N_2. The reaction was heated at 80 °C for six hours and cooled to ambient temperature. H_2O (200 mL) was added and extracted with Et_2O (4 × 300 mL). The combined organic fractions were dried over $MgSO_4$ and the solvent removed *in vacuo*. The crude material was purified by column chromatography (SiO_2; CH_2Cl_2/MeOH; 95:5) to obtain the title compound as a yellow oil (3.75 g, 22.0 mmol, 55%). ^{1}H NMR (300 MHz, 293 K, $CDCl_3$) 8.66 (ddd, $^3J_{HH}$ = 4.8, $^4J_{HH}$ = 1.8, $^5J_{HH}$ = 0.9, 1H, bipy<u>H</u>), 8.51 (dt, $^4J_{HH}$ = 2.3, $^5J_{HH}$ = 0.8, 1H, bipy<u>H</u>), 8.39 – 8.21 (m, 2H, bipy<u>H</u>), 7.80 (td, $^3J_{HH}$ = 7.7, $^4J_{HH}$ =1.8, 1H, bipy<u>H</u>), 7.63 (ddd, $^3J_{HH}$ = 8.1, $^4J_{HH}$ = 2.3, $^5J_{HH}$ = 0.8, 1H, bipy<u>H</u>), 7.34 – 7.26 (m, 1H, bipy<u>H</u>), 2.39 (s, 3H, ArC<u>H</u>$_2$).

Figure S2. ^{1}H NMR spectrum (300 MHz, 293 K, $CDCl_3$)

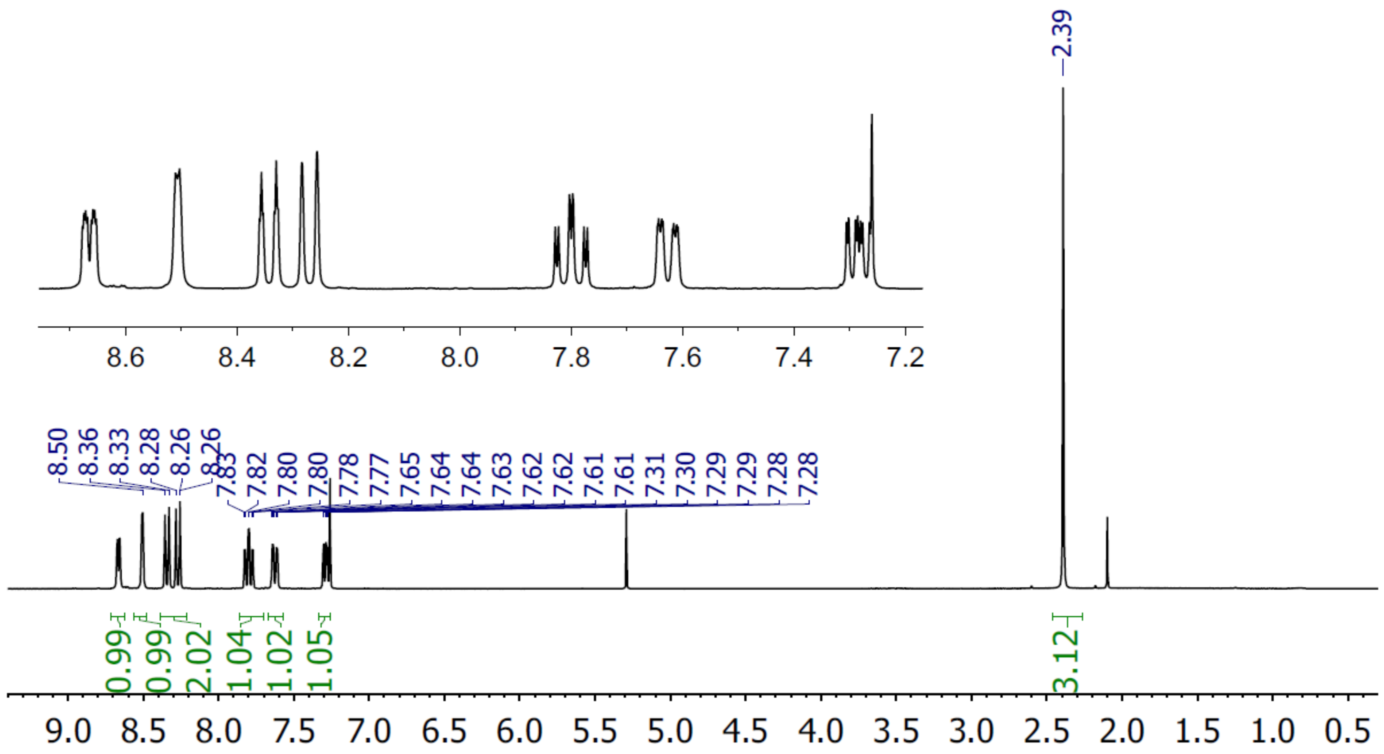

S1.1.3 Synthesis of [2,2'-bipyridine]-5-carboxylic acid

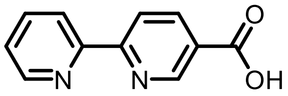

This compound was synthesised according to a modified literature procedure.[2]

5-methyl-2,2'-bipyridine (5.0 g, 29.4 mmol, 1.0 eq.) was suspended in H_2O/^{t}BuOH (1:1; 120 mL). $KMnO_4$ (18.6 g, 117.4 mmol, 4.0 eq.) was added portionwise over four hours. The reaction was heated at 80 °C for 18 hours. The reaction was filtered hot through Celite and washed with hot 2M $KOH_{(aq)}$ (2 × 20 mL). The combined aqueous fractions were concentrated *in vacuo* (*ca.* 40 mL) and acidified to pH = 4 with conc. $HCl_{(aq)}$ to give a precipitate. The solid was filtered and dried *in vacuo*, and coevaporated with EtOH (200 mL) to give the title compound as a white solid (4.87 g, 24.3 mmol, 83%). ^{1}H NMR (300 MHz, 293 K, $(CD_3)_2SO$) 9.15 (d, $^{4}J_{HH}$ = 2.0, 1H, bipyH), 8.77 – 8.69 (m, 1H, bipyH), 8.53 – 8.37 (m, 3H, bipyH), 8.05 – 7.94 (m, 1H, bipyH), 7.52 (ddd, $^{3}J_{HH}$ = 7.5, $^{4}J_{HH}$ = 4.8, $^{5}J_{HH}$ = 1.2 Hz, 1H, bipyH).

Figure S3. ^{1}H NMR spectrum (300 MHz, 293 K, $(CD_3)_2SO$)

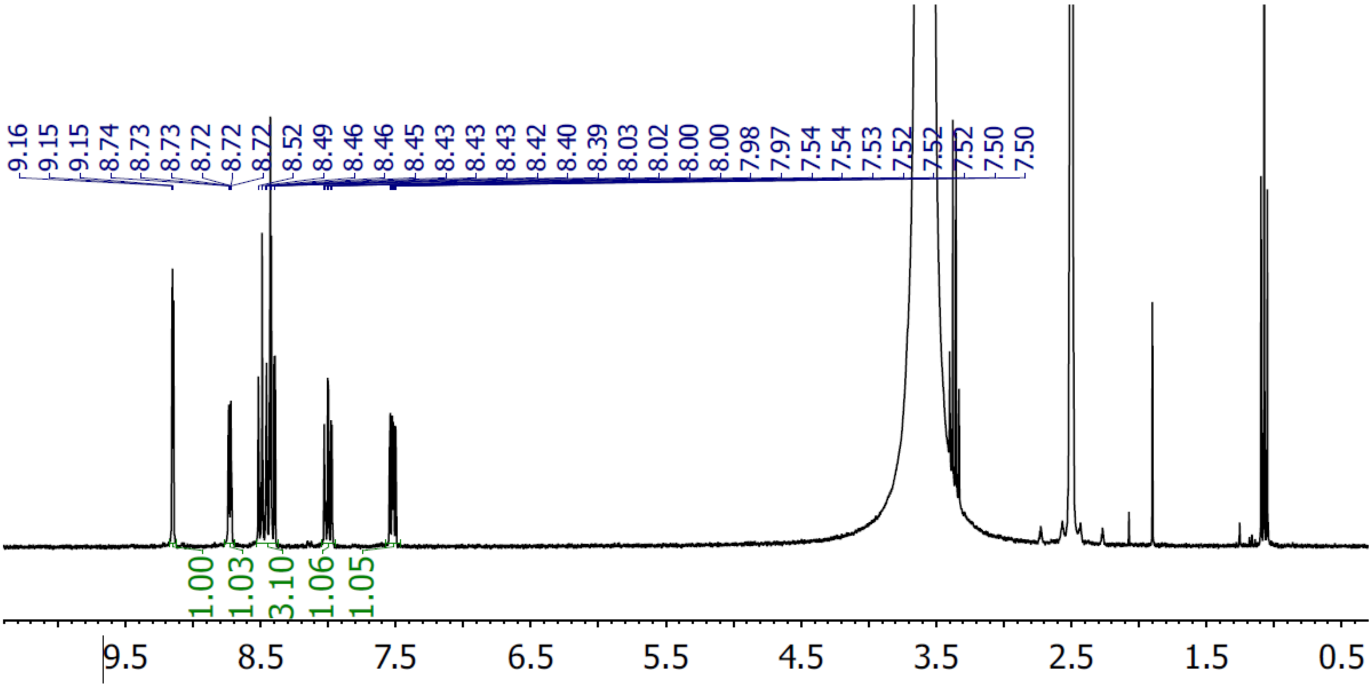

S1.1.4 Synthesis of ethyl [2,2'-bipyridine]-5-carboxylate

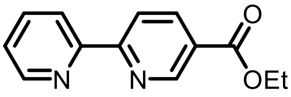

This compound was synthesised according to a modified literature procedure.[3]

[2,2'-bipyridine]-5-carboxylic acid (1.04 g, 5.2 mmol) was dissolved in EtOH (50 mL). H_2SO_4 (10 mL) was added slowly and the reaction heated to reflux for 18 hours. The reaction was neutralised with sat. $NaHCO_{3(aq)}$ and extracted with CH_2Cl_2 (3 × 50 mL). The combined organic layers were dried over $MgSO_4$ and the solvent removed *in vacuo*. The crude material was purified by column chromatography (SiO_2; EtOAc/petroleum ether; 0:100→50:50) to give the title compound as a white solid (423 mg, 1.85 mmol, 36%). ^{1}H NMR (300 MHz, 293 K, $CDCl_3$) 9.27 (dd, $^{4}J_{HH}$ = 2.1, $^{5}J_{HH}$ = 0.9, 1H, bipyH), 8.71 (ddd, $^{3}J_{HH}$ = 4.8, $^{4}J_{HH}$ = 1.8, $^{5}J_{HH}$ = 0.9, 1H, bipyH), 8.52 – 8.38 (m, 3H, bipyH), 7.85 (ddd, $^{3}J_{HH}$ = 8.0, 7.5, $^{4}J_{HH}$ = 1.8, 1H, bipyH), 7.36 (ddd, $^{3}J_{HH}$ = 7.5, 4.8, $^{4}J_{HH}$ 1.2, 1H, bipyH), 4.43 (q, $^{3}J_{HH}$ = 7.1, 2H, $C\underline{H}_2CH_3$), 1.43 (t, $^{3}J_{HH}$ = 7.1, 3H, $CH_2C\underline{H}_3$).

Figure S4. ^{1}H NMR spectrum (300 MHz, 293 K, $CDCl_3$)

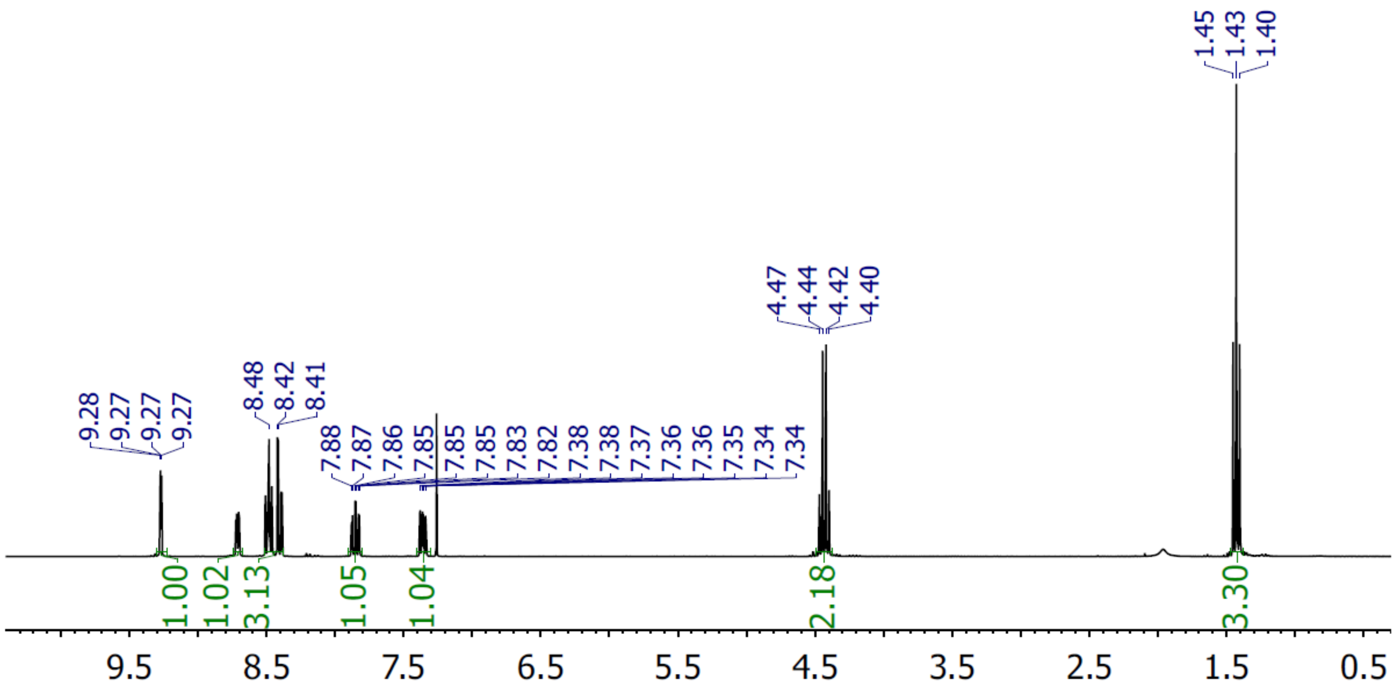

S1.1.5 Synthesis of [2,2'-bipyridin]-5-ylmethanol

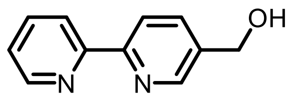

This compound was synthesised according to a modified literature procedure.[4]

ethyl [2,2'-bipyridine]-5-carboxylate (400 mg, 1.75 mmol, 1.0 eq.) was dissolved in EtOH (25 mL). $NaBH_4$ (199 mg, 5.25 mmol, 3.0 eq.) was added portionwise over 10 minutes and the reaction heated to reflux for four hours. The reaction was cooled to ambient temperature and sat. $NH_4Cl_{(aq)}$ (50 mL) was added. The EtOH was removed *in vacuo* and the aqueous mixture extracted with CH_2Cl_2 (3 × 50 mL). The combined organic fractions were dried over $MgSO_4$ and the solvent removed *in vacuo*. The crude material was purified by column chromatography (SiO_2; CH_2Cl_2/MeOH/$NH_4OH_{(aq)}$; 94:5:1) to give the title compound as a white solid (231 mg, 1.24 mmol, 71%). ^{1}H NMR (300 MHz, 293 K, $CDCl_3$) 8.56 (ddd, $^{3}J_{HH}$ = 4.9, $^{4}J_{HH}$ = 1.8, $^{5}J_{HH}$ = 0.9, 1H, bipyH), 8.45 (dt, $^{4}J_{HH}$ = 2.2, $^{5}J_{HH}$ 0.7, 1H, bipyH), 8.23 – 8.09 (m, 2H, bipyH), 7.77 – 7.62 (m, 2H, bipyH), 7.23 (ddd, $^{3}J_{HH}$ = 7.5, 4.8, $^{4}J_{HH}$ = 1.2 Hz, 1H, bipyH), 4.94 (s, 1H, CH_2OH), 4.61 (s, 2H, CH_2OH).

Figure S5. ^1H NMR spectrum (300 MHz, 293 K, $CDCl_3$)

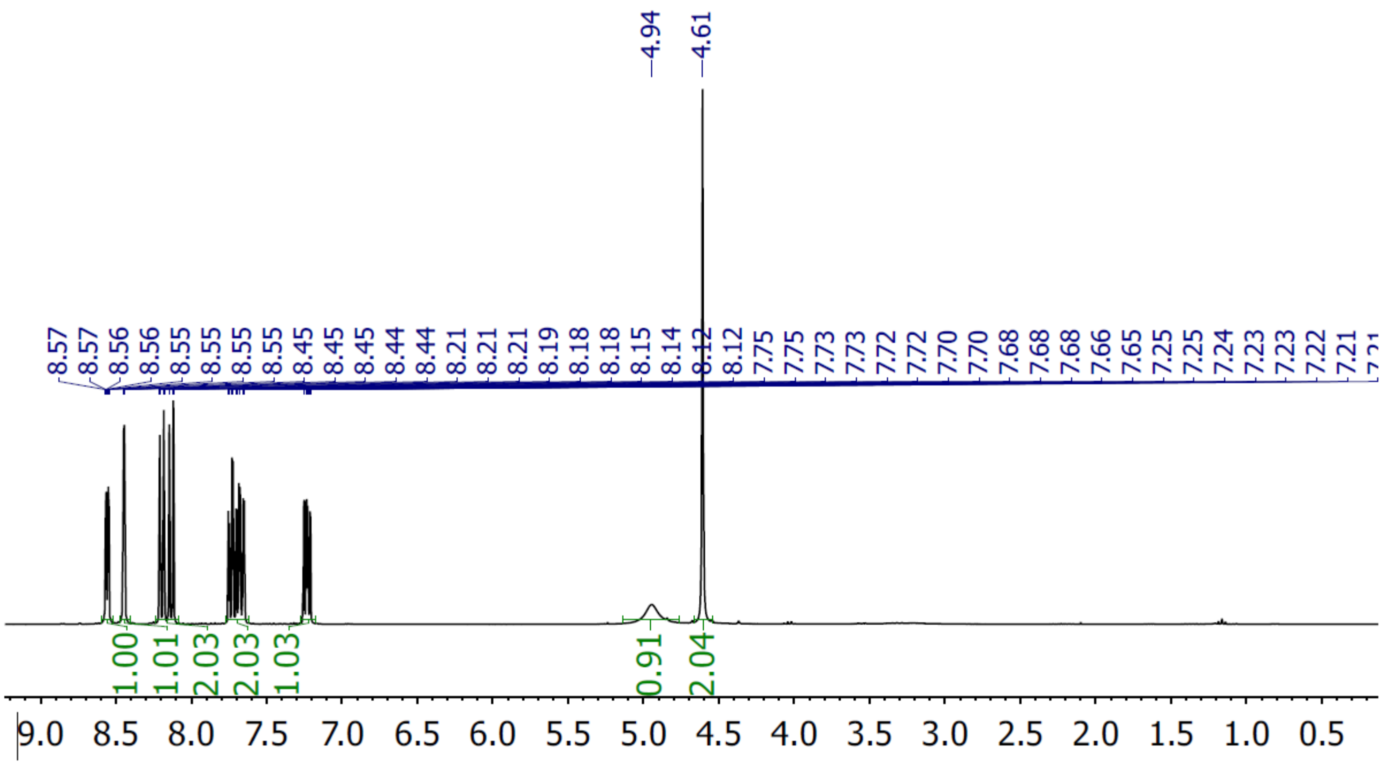

S1.1.6 Synthesis of 5-(chloromethyl)-2,2'-bipyridine

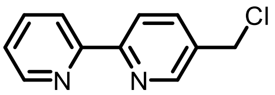

This compound was synthesised according to a modified literature procedure.[5]

[2,2'-bipyridin]-5-ylmethanol (1.2 g, 6.44 mmol, 1.0 eq.) was dissolved in CH_2Cl_2 (160 mL). $SOCl_2$ (0.94 mL, 12.88 mmol, 2.0 eq.) was added dropwise and the reaction stirred at ambient temperature for 18 hours. The solvent was removed *in vacuo* and resuspended in CH_2Cl_2 (100 mL). The organic solution was washed with sat. $NaHCO_3$ (3 × 100 mL) and dried over $MgSO_4$. The solvent was removed *in vacuo* to give the title compound as an off-white solid (1.24 g, 6.1 mmol, 94%). ^1H NMR (300 MHz, 293 K, $CDCl_3$) 8.68 (ddd, $^3J_{HH}$ = 4.8, $^4J_{HH}$ = 1.9, $^5J_{HH}$ = 0.9, 2H, bipyH), 8.44 – 8.36 (m, 2H, bipyH), 7.89 – 7.78 (m, 2H, bipyH), 7.32 (ddd, $^3J_{HH}$ = 7.5, 4.8, $^4J_{HH}$ = 1.2, 1H, bipyH), 4.66 – 4.65 (m, 2H, CH$_2$Cl).

Figure S6. ^{1}H NMR spectrum (300 MHz, 293 K, $CDCl_3$)

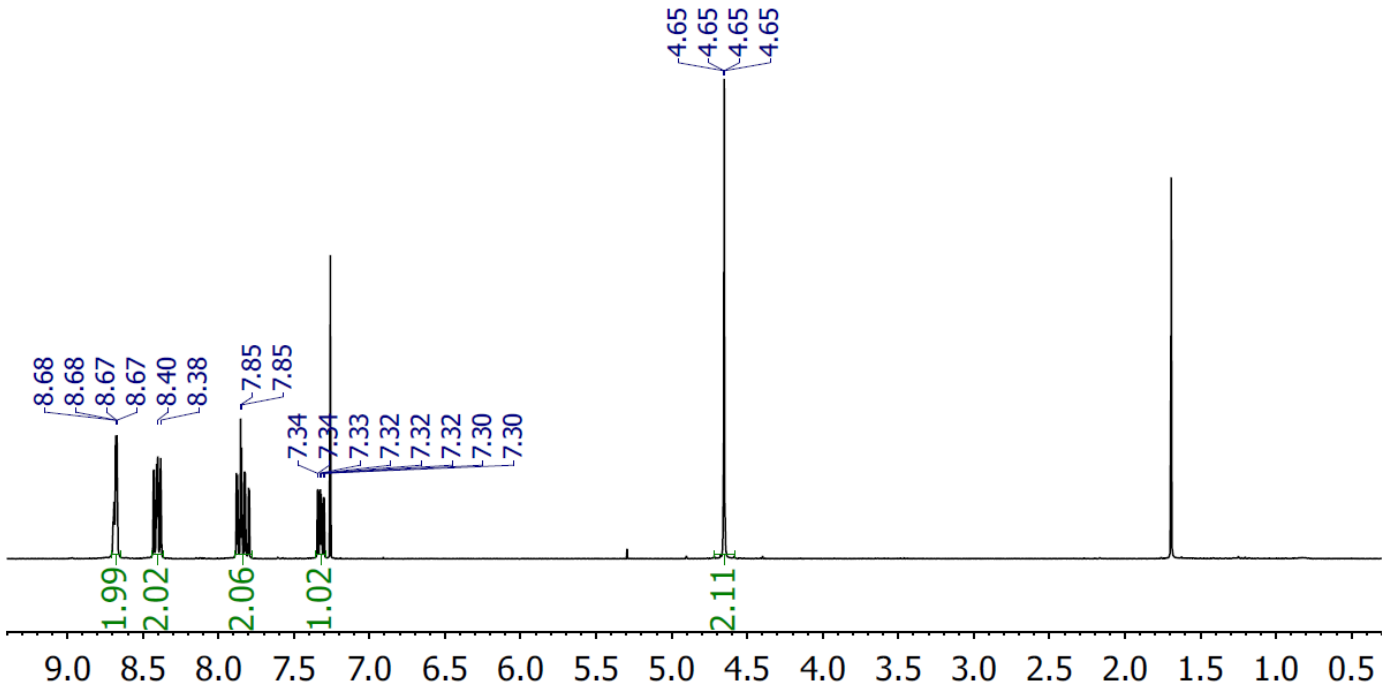

S1.1.7 Synthesis of [2,2'-bipyridine]-5,5'-dicarboxylic acid

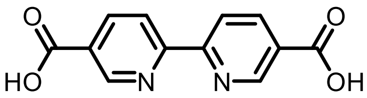

This compound was synthesised according to a modified literature procedure.[6]

5,5'-dimethyl-2,2'-bipyridine (2.0 g, 10.85 mmol, 1.0 eq.) and $KMnO_4$ (11.1 g, 70.5 mmol, 6.5 eq.) was dissolved in H_2O (70 mL) and heated to reflux for two hours. The reaction was cooled, filtered through Celite and washed with hot H_2O (2 × 10 mL). The aqueous solution was cooled to 4 °C and acidified with conc. $HCl_{(aq)}$ to obtain a white precipitate which was filtered and dried *in vacuo* to obtain the target compound as a white solid (1.90 g, 7.8 mmol, 72%). ^{1}H NMR (300 MHz, 293 K, $(CD_3)_2SO$) 9.19 (d, $^4J_{HH}$ = 2.2, $^5J_{HH}$ = 0.9, 2H, bipyH), 8.56 (dd, $^3J_{HH}$ = 8.3, $^5J_{HH}$ = 0.9 Hz, 2H, bipyH), 8.44 (dd, $^3J_{HH}$ = 8.3, $^4J_{HH}$ =2.2 Hz, 2H).

Figure S7. ^{1}H NMR spectrum (300 MHz, 293 K, $(CD_3)_2SO$)

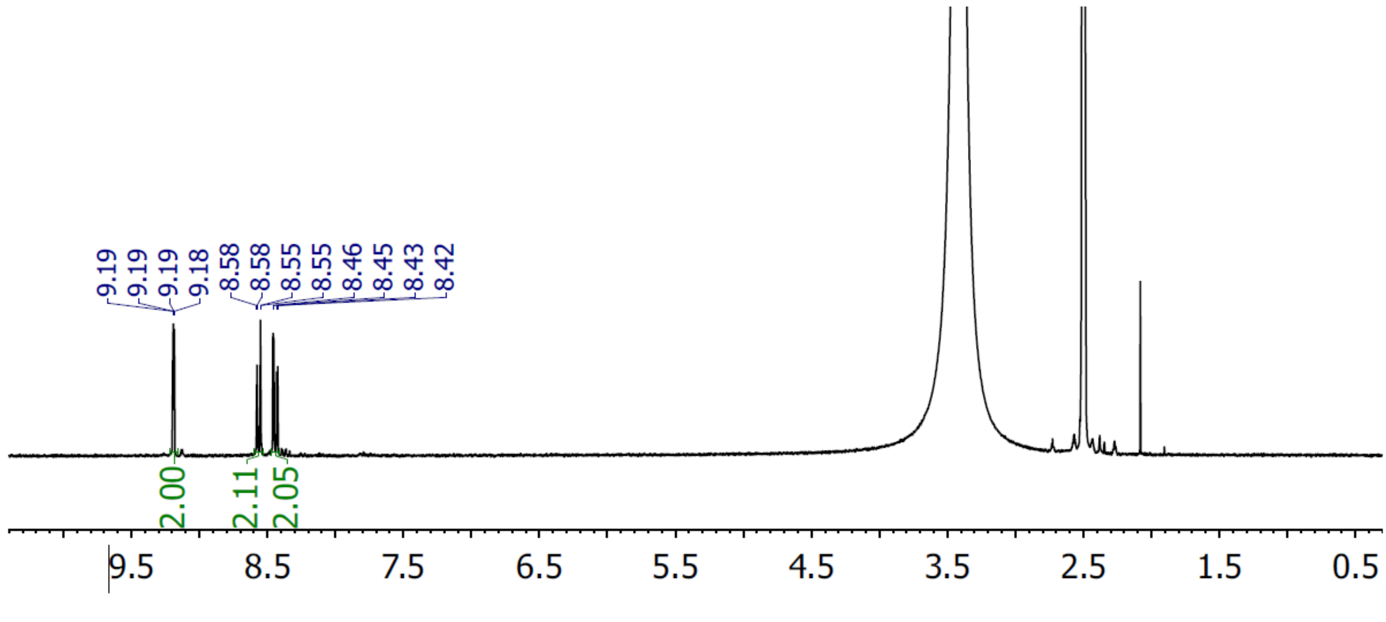

S1.1.8 Synthesis of diethyl [2,2'-bipyridine]-5,5'-dicarboxylate

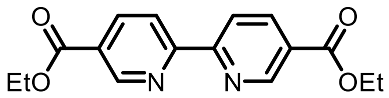

This compound was synthesised according to a modified literature procedure[3].

[2,2'-bipyridine]-5,5'-dicarboxylic acid (1.90 g, 7.8 mmol) was dissolved in EtOH (50 mL). H_2SO_4 (10 mL) was slowly aadded and the reaction heated to reflux for 18 hours. The reaction was cooled and basified with sat. $NaHCO_{3(aq)}$. The aqueous mixtures was extracted with CH_2Cl_2 (3 × 50 mL) and dried over $MgSO_4$. Removal of the solvent *in vacuo* gave the title compound as a white solid (1.38 g, 4.6 mmol, 59%). ^{1}H NMR (300 MHz, 293 K, $CDCl_3$) 9.29 (dd, $^4J_{HH}$ = 2.1, $^5J_{HH}$ = 0.9, 2H, bipy$\underline{H}$), 8.57 (dd, $^3J_{HH}$ = 8.3, $^5J_{HH}$ = 0.9, 2H, bipy$\underline{H}$), 8.43 (dd, $^3J_{HH}$ = 8.3, $^4J_{HH}$ = 2.1, 2H, bipy$\underline{H}$), 4.45 (q, $^3J_{HH}$ = 7.1, 4H, C$\underline{H}_2$CH$_3$), 1.44 (t, $^3J_{HH}$ = 7.1, 6H, CH$_2$C$\underline{H}_3$).

Figure S8. ^{1}H NMR spectrum (300 MHz, 293 K, $CDCl_3$)

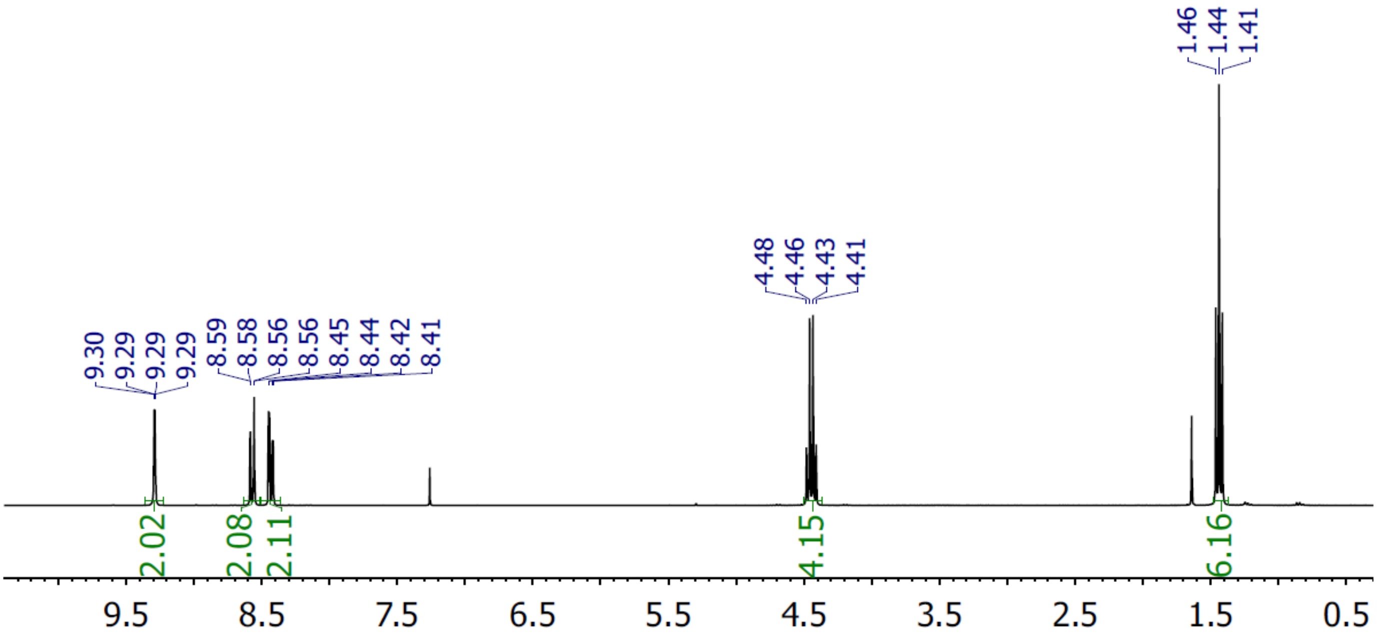

S1.1.9 Synthesis of [2,2'-bipyridine]-5,5'-diyldimethanol

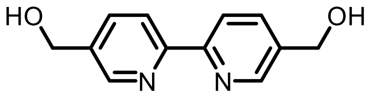

This compound was synthesised according to a modified literature procedure.[4]

diethyl [2,2'-bipyridine]-5,5'-dicarboxylate (1.38 g, 4.6 mmol, 1.0 eq.) was dissolved in EtOH (200 mL). $NaBH_4$ (1.74 g, 46.0 mmol, 10.0 eq.) was added and the reaction was heated to reflux for four hours. The reaction was cooled to ambient temperature and sat. $NH_4Cl_{(aq)}$ (200 mL) was added. The EtOH was removed *in vacuo* and the aqueous mixtures extracted with EtOAc (3 × 100 mL), dried over $MgSO_4$ and the solvent removed *in vacuo* to give the title compound as a white solid (791 mg, 3.66 mmol, 79%). ^{1}H NMR (500 MHz, 293 K, $CDCl_3$) 8.64 – 8.63 (m, 2H, bipy$\underline{H}$), 8.33 – 8.23 (m, 2H, bipy$\underline{H}$), 7.94 – 7.91 (m, 2H, bipy$\underline{H}$), 4.72 (s, 4H, C$\underline{H}_2$OH).

Figure S9. ^{1}H NMR spectrum (500 MHz, 293 K, $CDCl_3$)

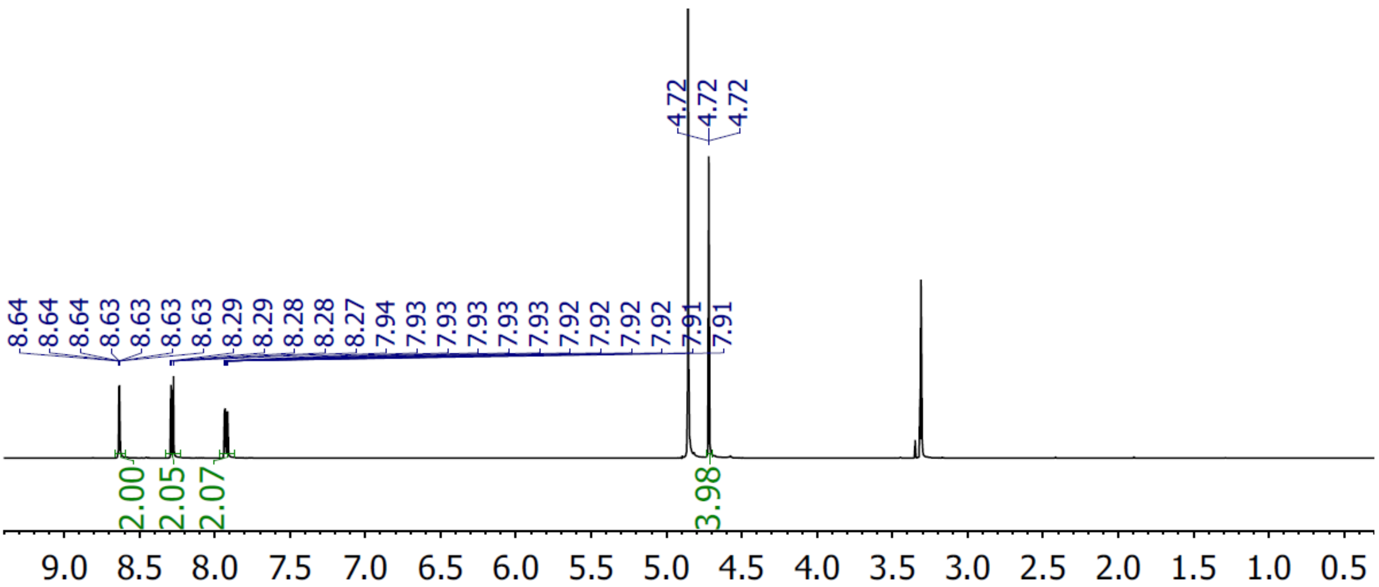

S1.1.10 Synthesis of 5,5'-bis(chloromethyl)-2,2'-bipyridine

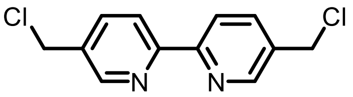

This compound was synthesised according to a modified literature procedure.[5]

[2,2'-bipyridine]-5,5'-diyldimethanol (791 mg, 3.65 mmol, 1.0 eq.) was suspended in CH_2Cl_2 (50 mL) and $SOCl_2$ (1.07 mL, 14.6 mmol, 4.0 eq.) was added dropwise. The reaction was stirred at ambient temperature for 36 hours until the reaction was homogeneous. The solvent was removed *in vacuo*, resuspended in H_2O (50 mL) and extracted with CH_2Cl_2 (3 × 30 mL). The organic layer was dried over $MgSO_4$ and the solvent removed *in vacuo* to give the title compound as an off-white solid (580 mg, 2.3 mmol, 63%). ^{1}H NMR (300 MHz, 293 K, $CDCl_3$) 8.68 (dd, $^{4}J_{HH}$ = 2.3, $^{5}J_{HH}$ = 0.8, 2H, bipyH), 8.42 (dd, $^{4}J_{HH}$ = 8.2, $^{5}J_{HH}$ = 0.8, 2H. bipyH), 7.91 – 7.83 (m, 2H, bipyH), 4.66 (s, 4H, CH_2Cl).

Figure S10. ^{1}H NMR spectrum (300 MHz, 293 K, $CDCl_3$)

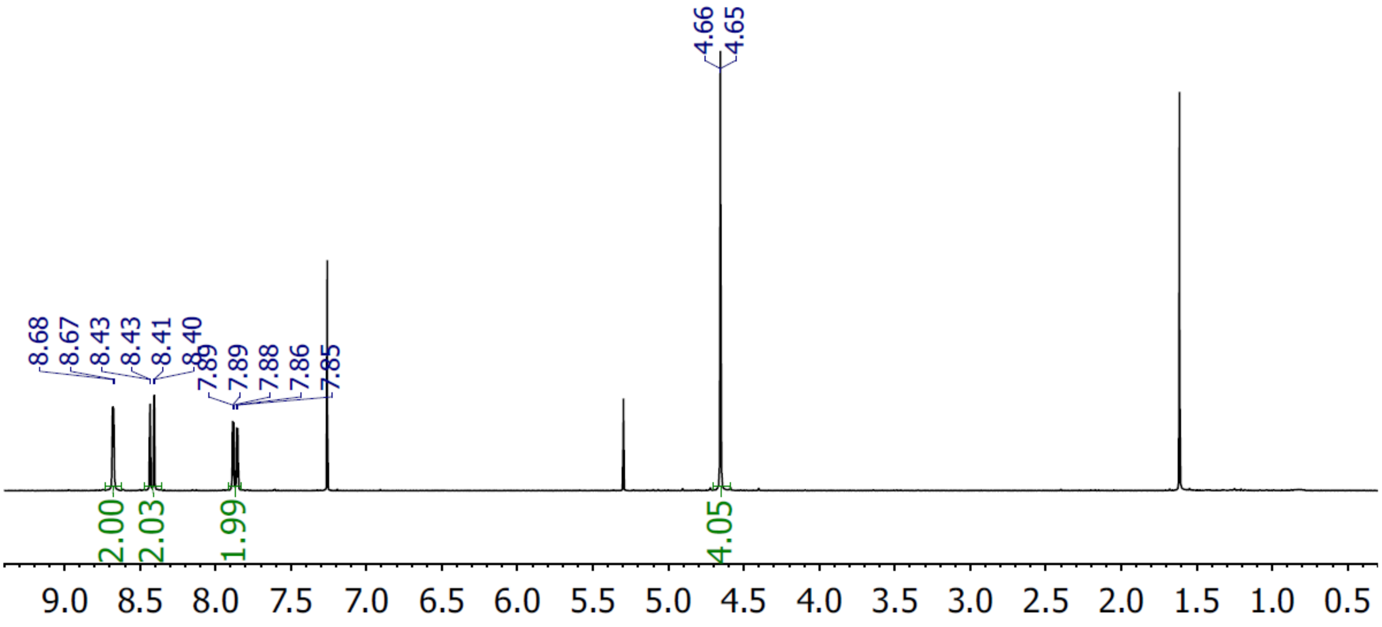

S1.1.11 Synthesis of 3-([2,2'-bipyridin]-5-ylmethyl)-1-methyl-1H-imidazol-3-ium chloride

Figure S11. ^{1}H NMR spectrum (500 MHz, 293 K, CD_3OD)

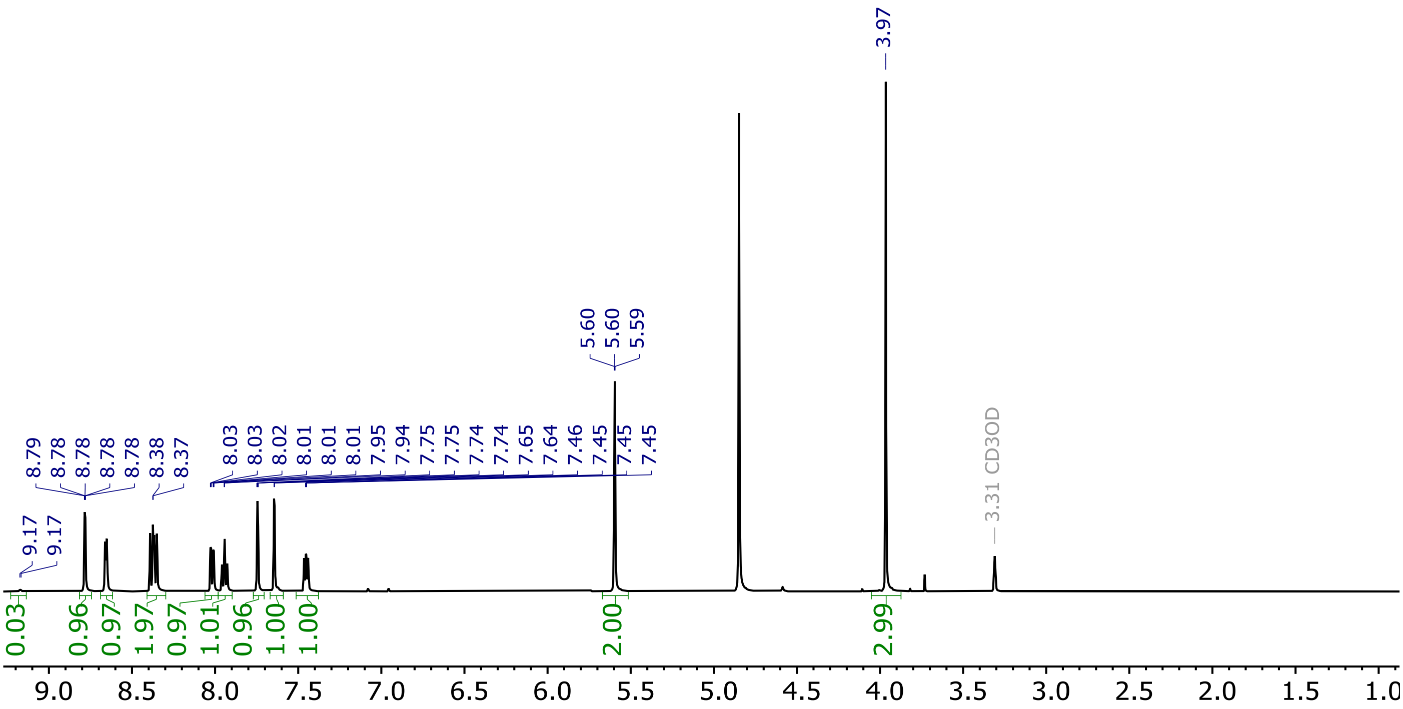

Figure S12. ^{13}C NMR spectrum (126 MHz, 293 K, CD_3OD)

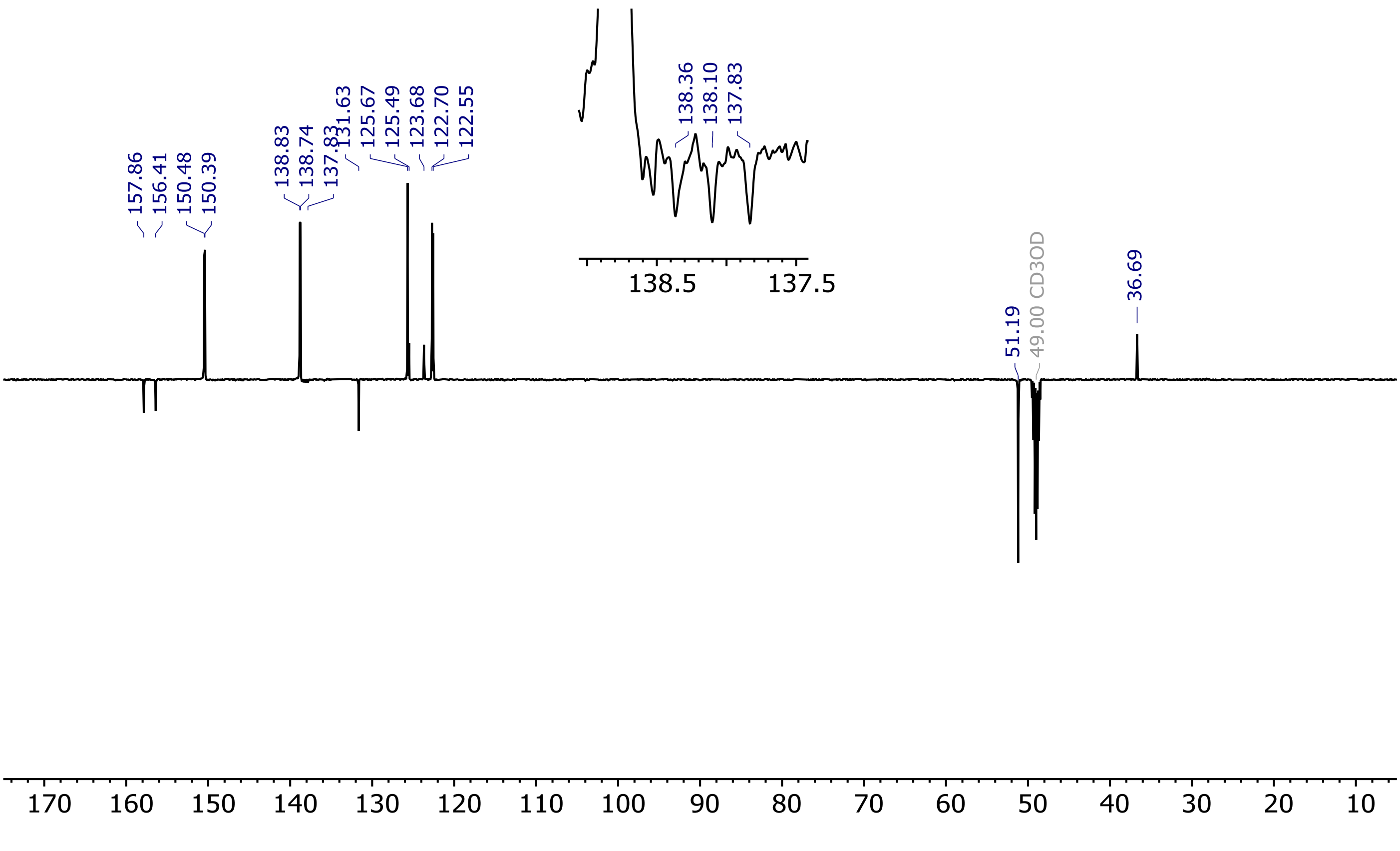

Figure S13 .ESI-MS spectrum

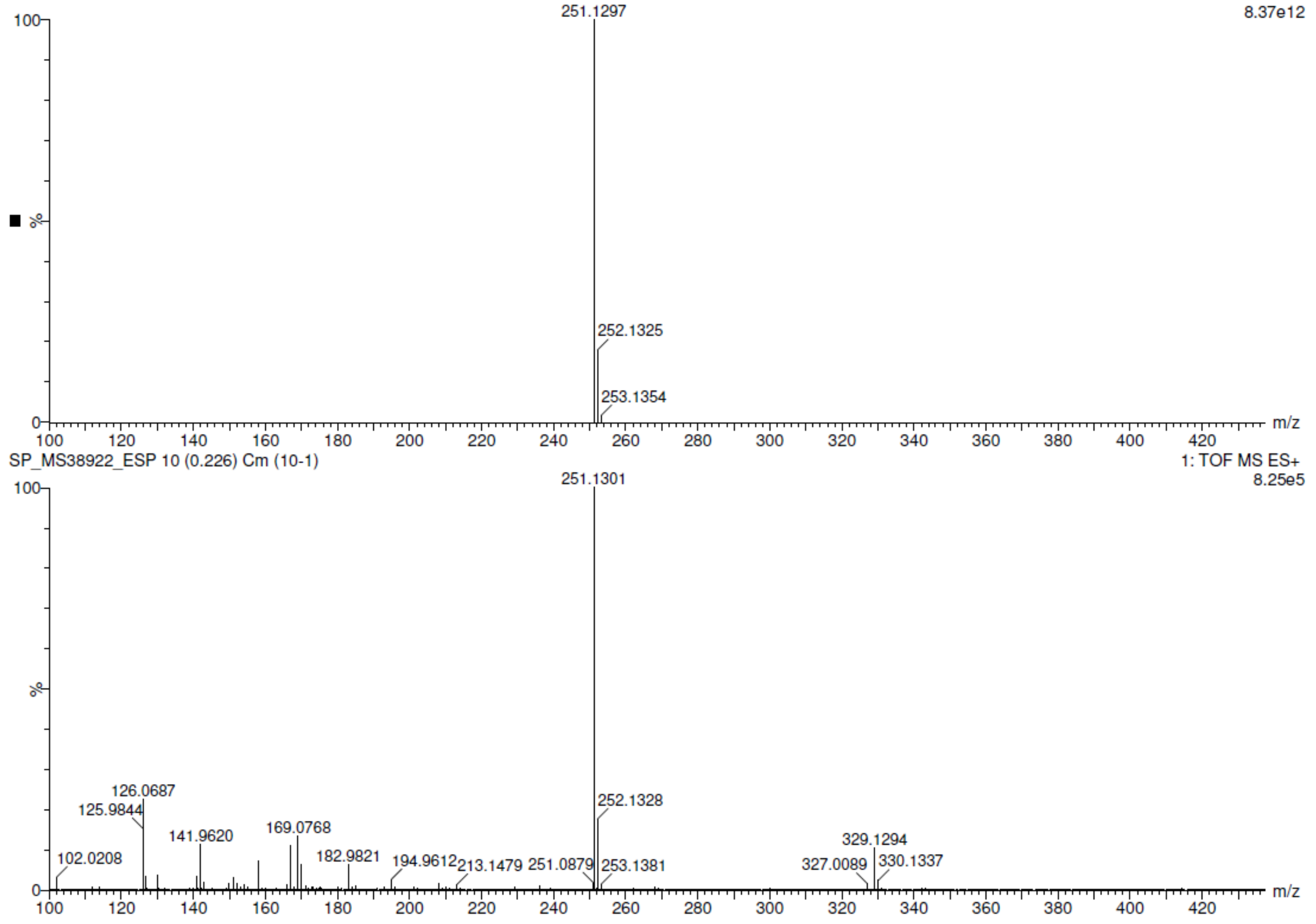

S1.1.12 Synthesis of 3,3'-([2,2'-bipyridine]-5,5'-diylbis(methylene))bis(1-methyl-1H-imidazol-3-ium) dichloride

Figure S14.^{1}H NMR spectrum (500 MHz, 293 K, CD_3OD)

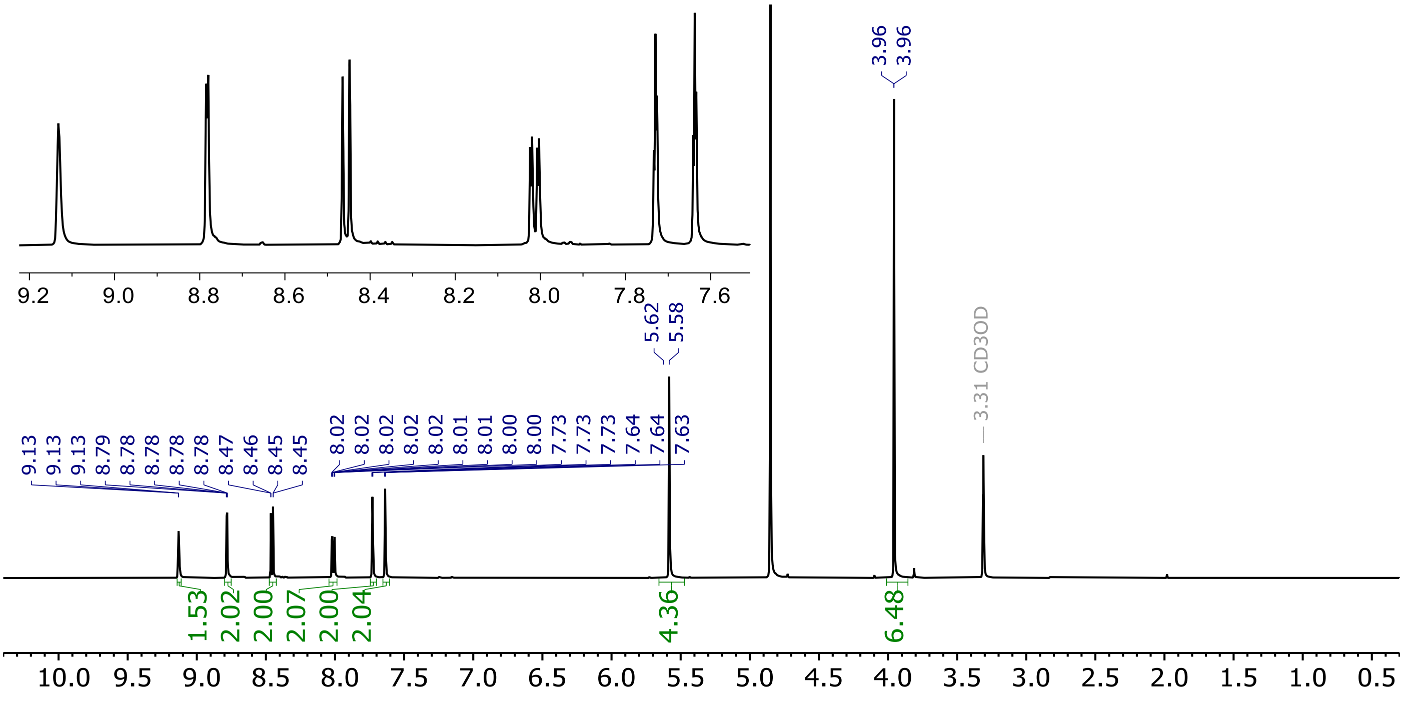

Figure S15.^{13}C NMR spectrum (126 MHz, 293 K, CD_3OD)

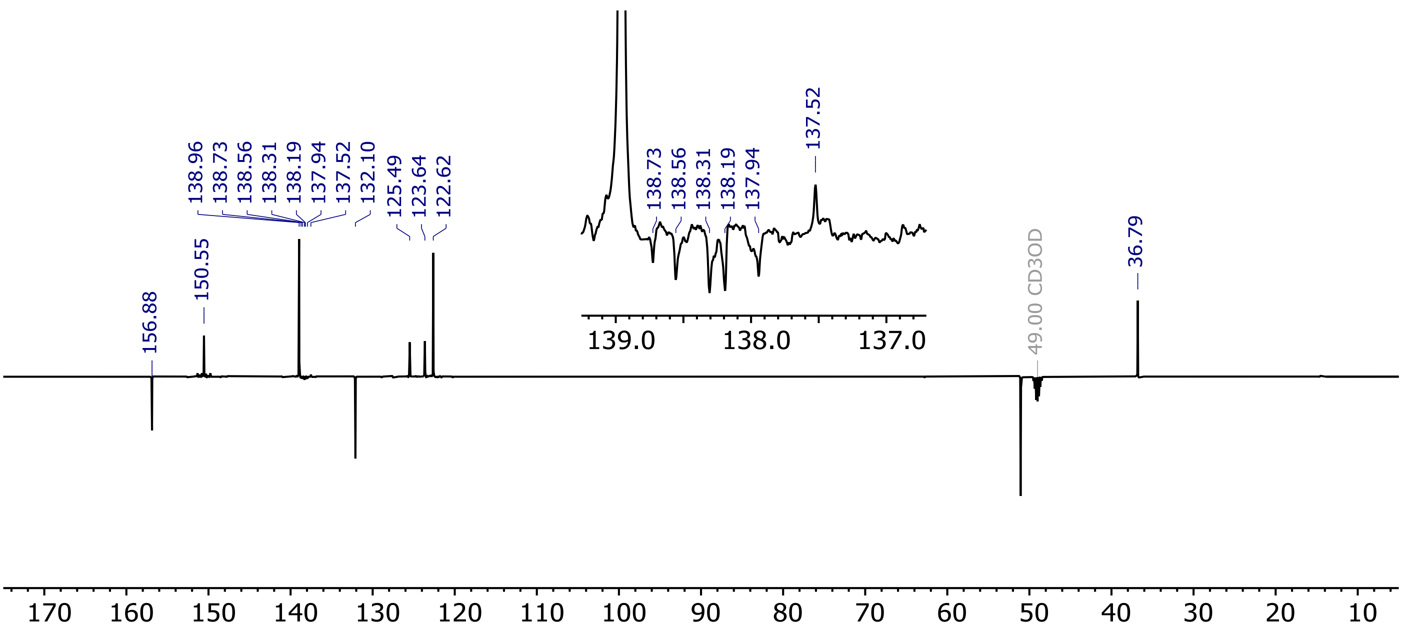

Figure S16. ESI-MS spectrum

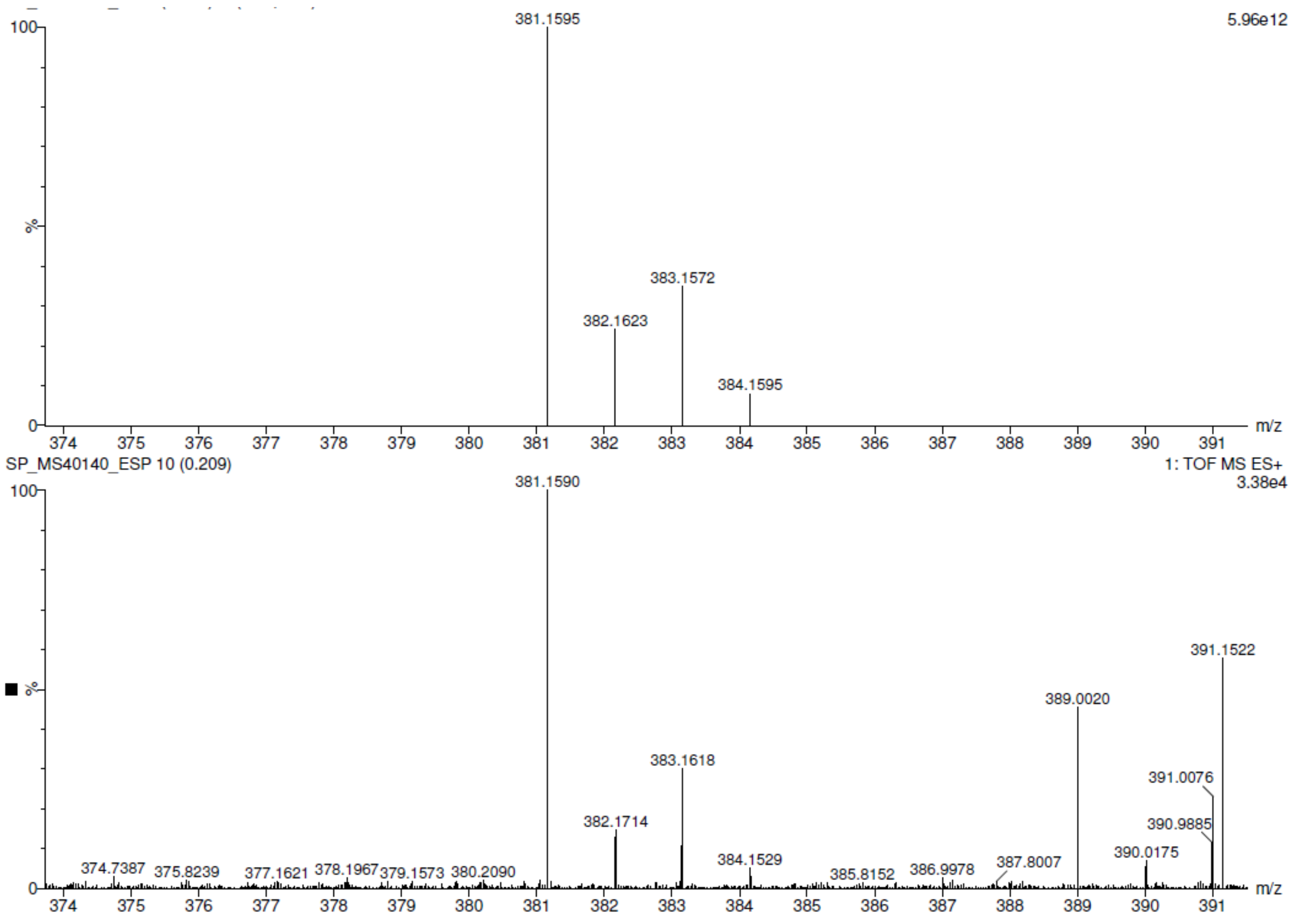

S1.2 Complex Spectra

S1.2.1 Synthesis of [Ru(bipy)$_2$(L1)$_1$][PF$_6$]$_3$

Figure S17. ^{1}H NMR spectrum (500 MHz, 293 K, CD_3CN)

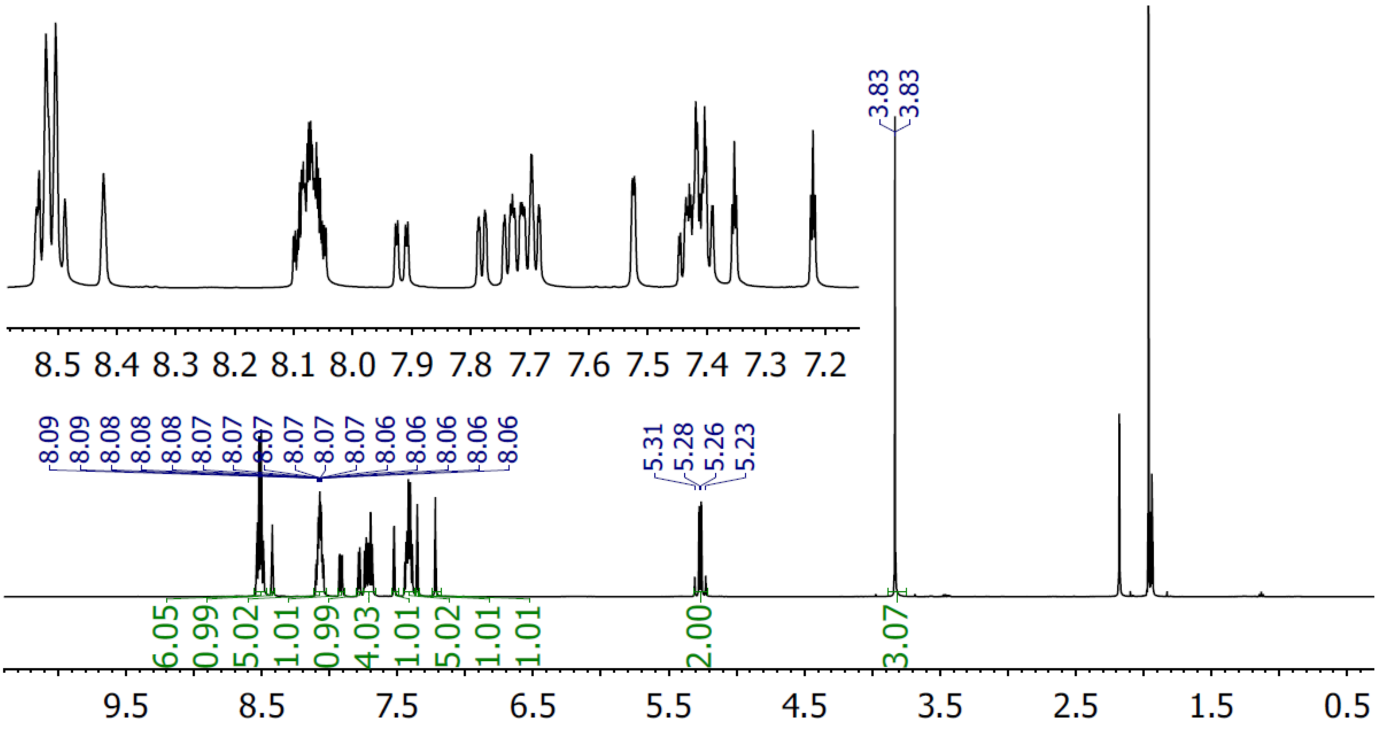

Figure S18. ^{13}C NMR spectrum (126 MHz, 293 K, CD_3CN)

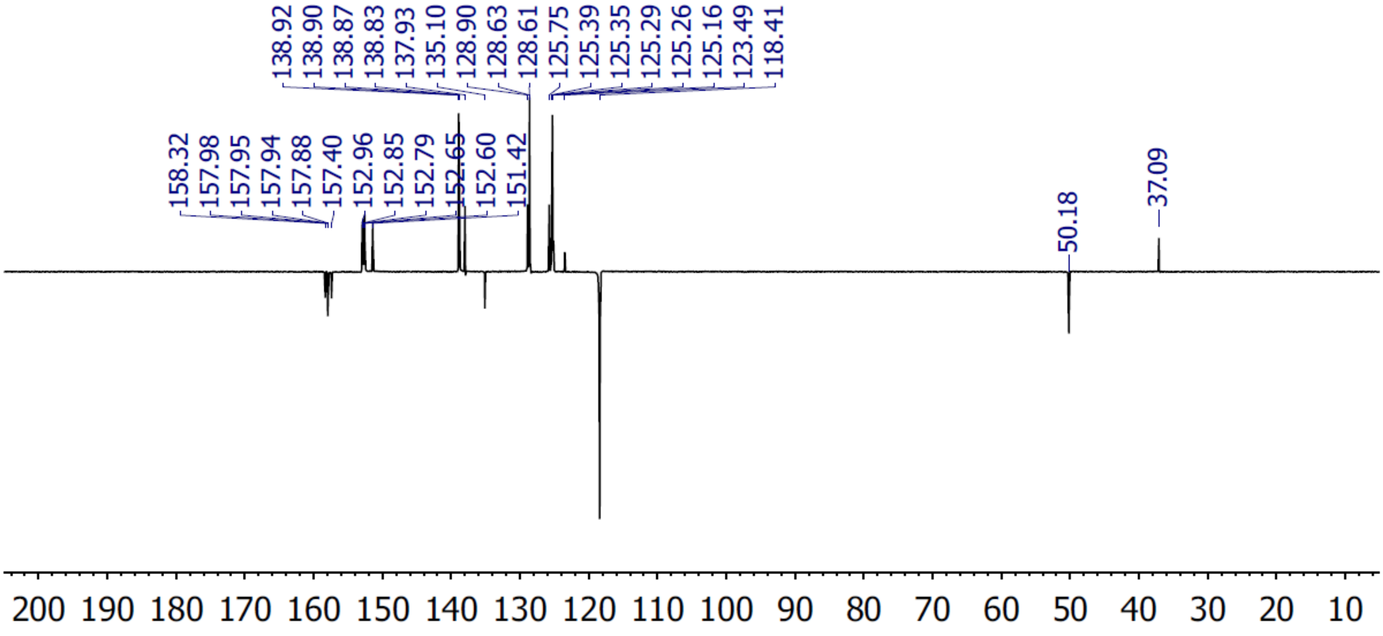

Figure S19. ESI-MS spectrum

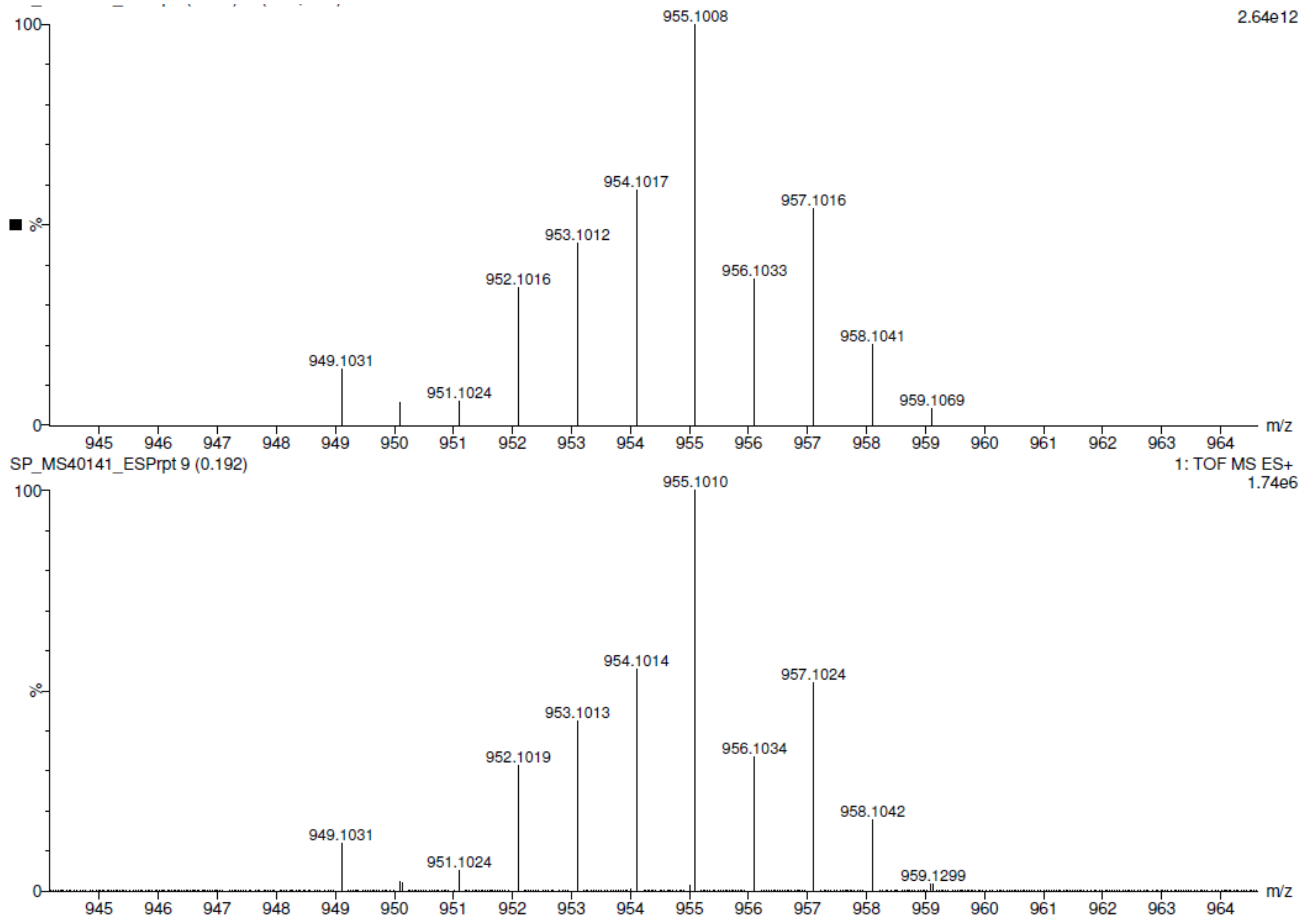

S1.2.2 Synthesis of $[Ru(bipy)_2(L2)_1][PF_6]_4$

Figure S20. ^{1}H NMR spectrum (500 MHz, 293 K, CD_3CN)

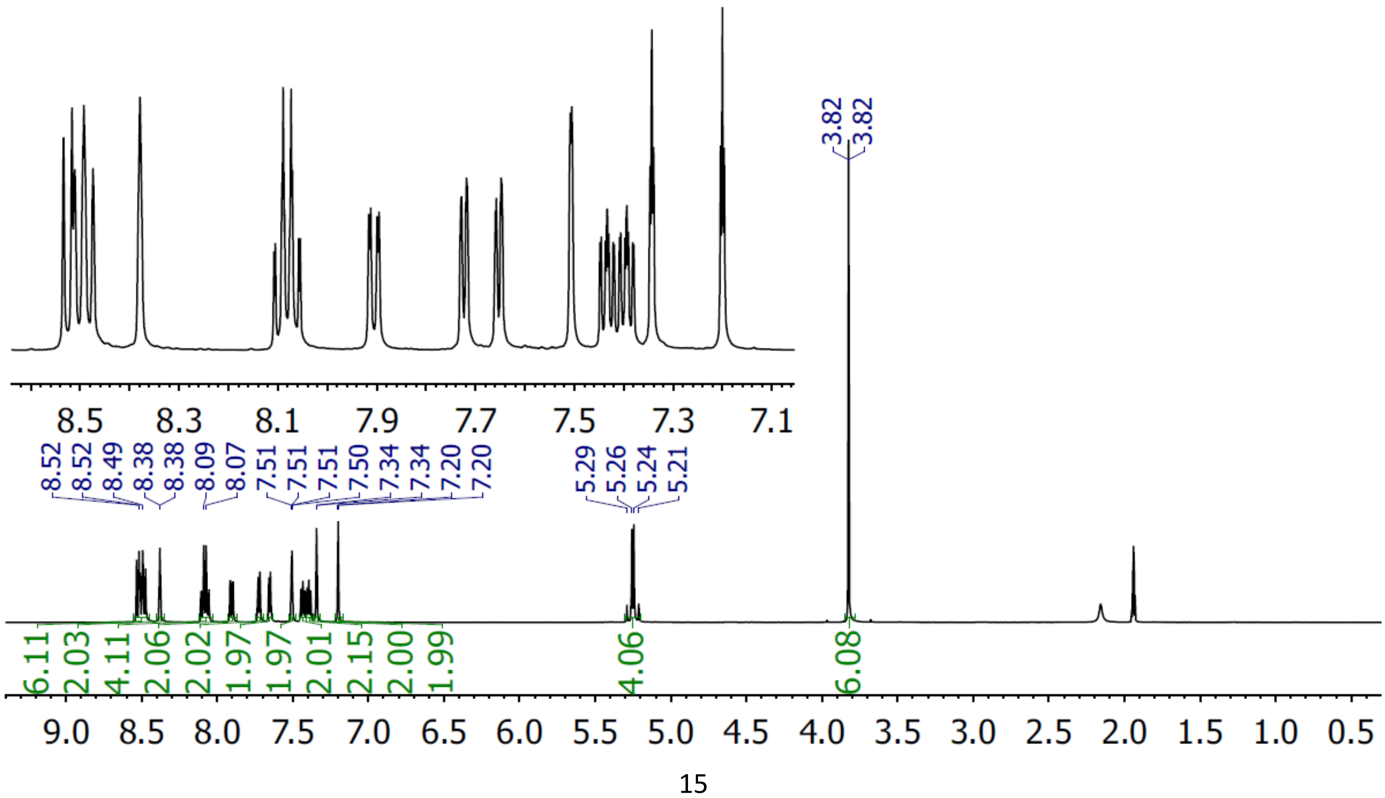

Figure S21. ^{13}C NMR spectrum (126 MHz, 293 K, CD_3CN)

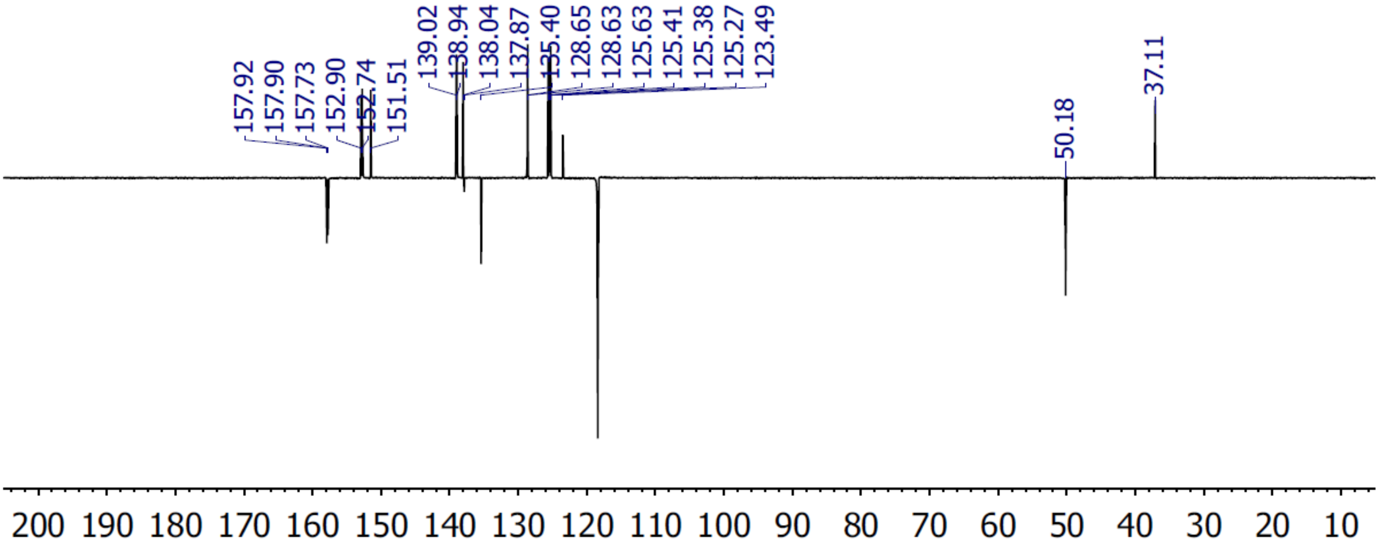

Figure S22. ESI-MS spectrum

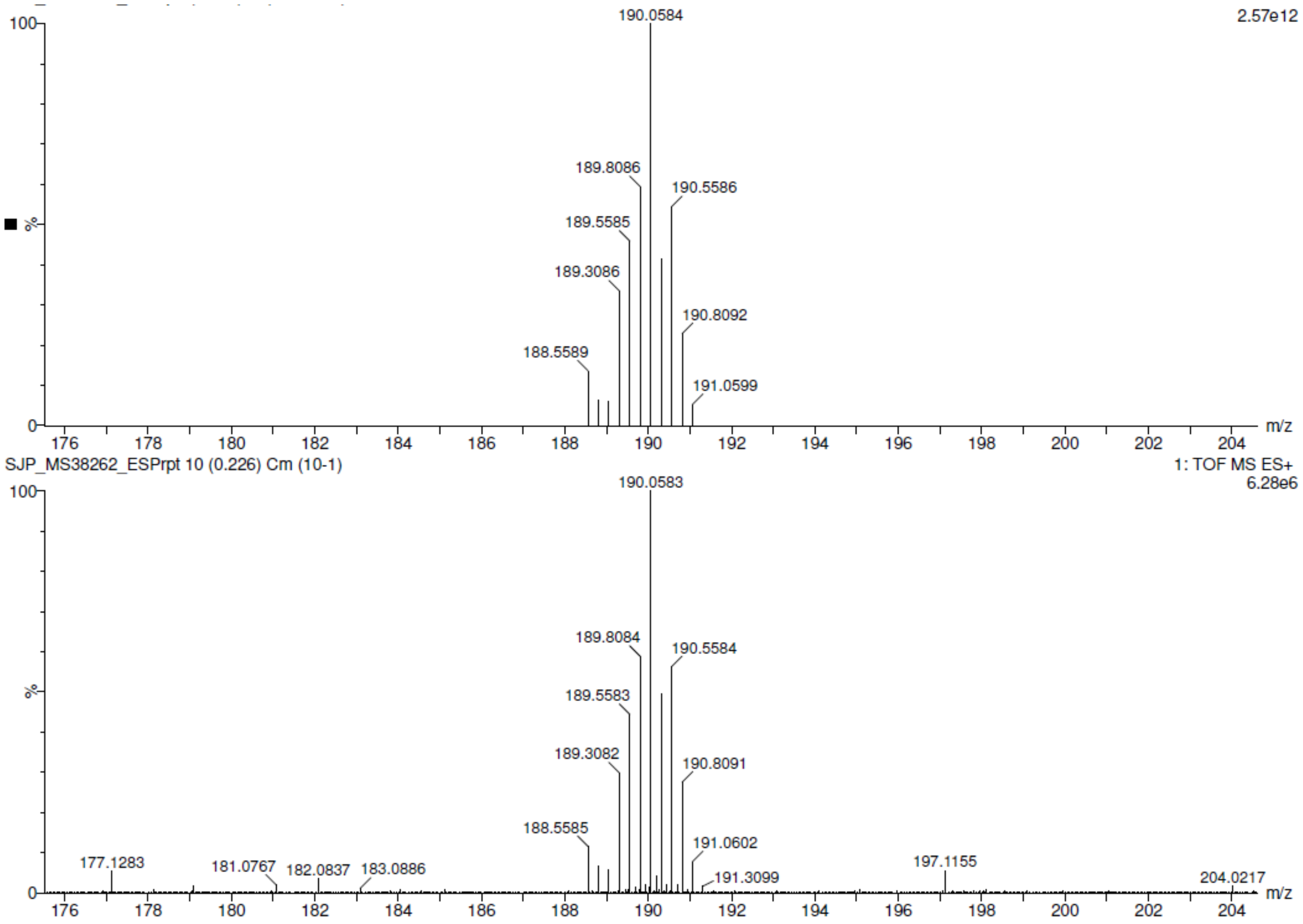

S1.2.3 Synthesis of [Ru(L_2)Cl_2]Cl_4

Figure S23. ^{1}H NMR spectrum (300 MHz, 293 K, CD_3OD)

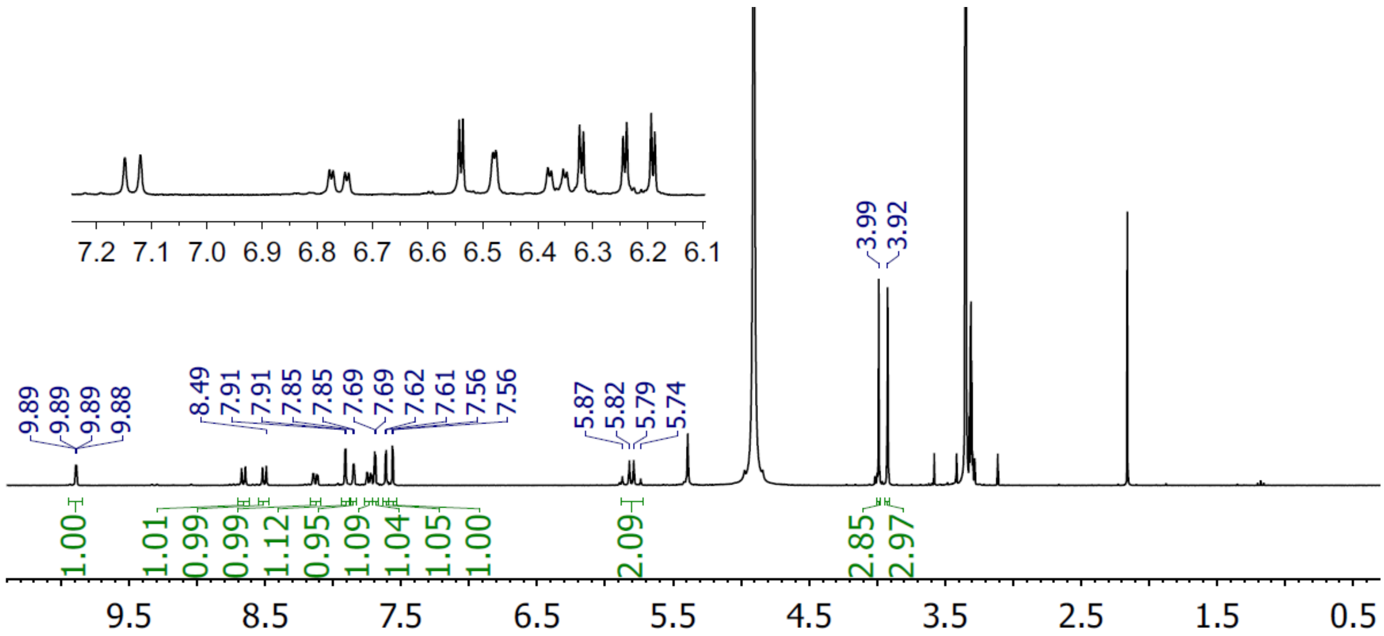

Figure S24. ^{13}C NMR spectrum (126 MHz, 293 K, CD_3OD)

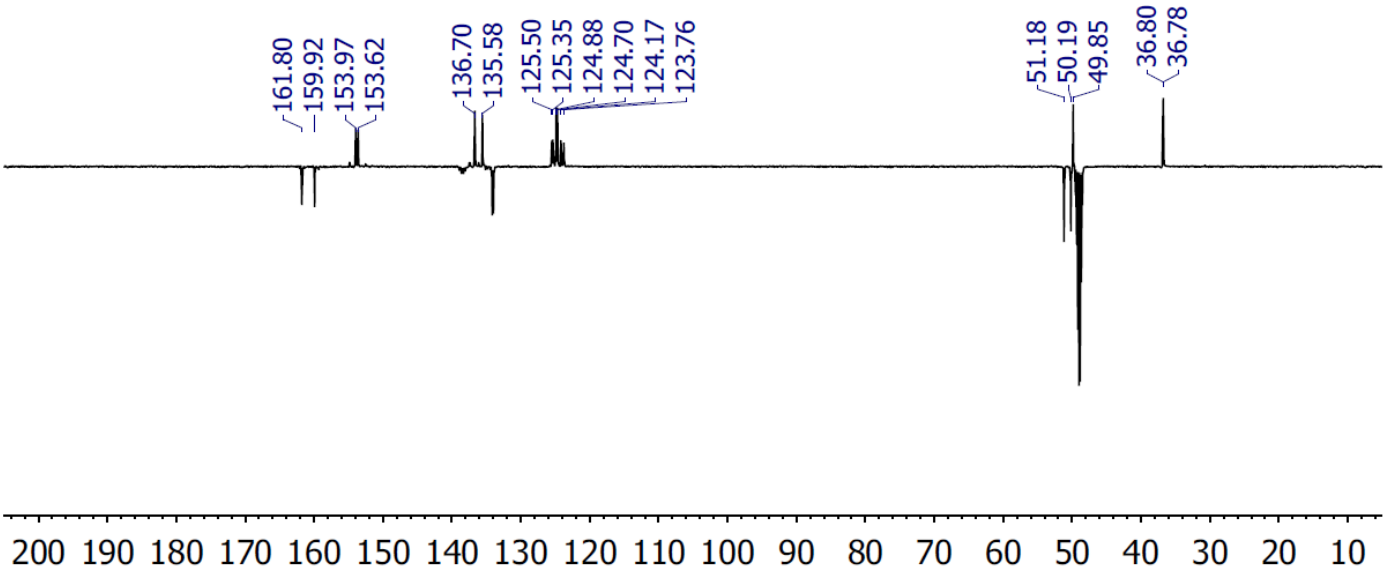

Figure S25. ESI-MS spectrum

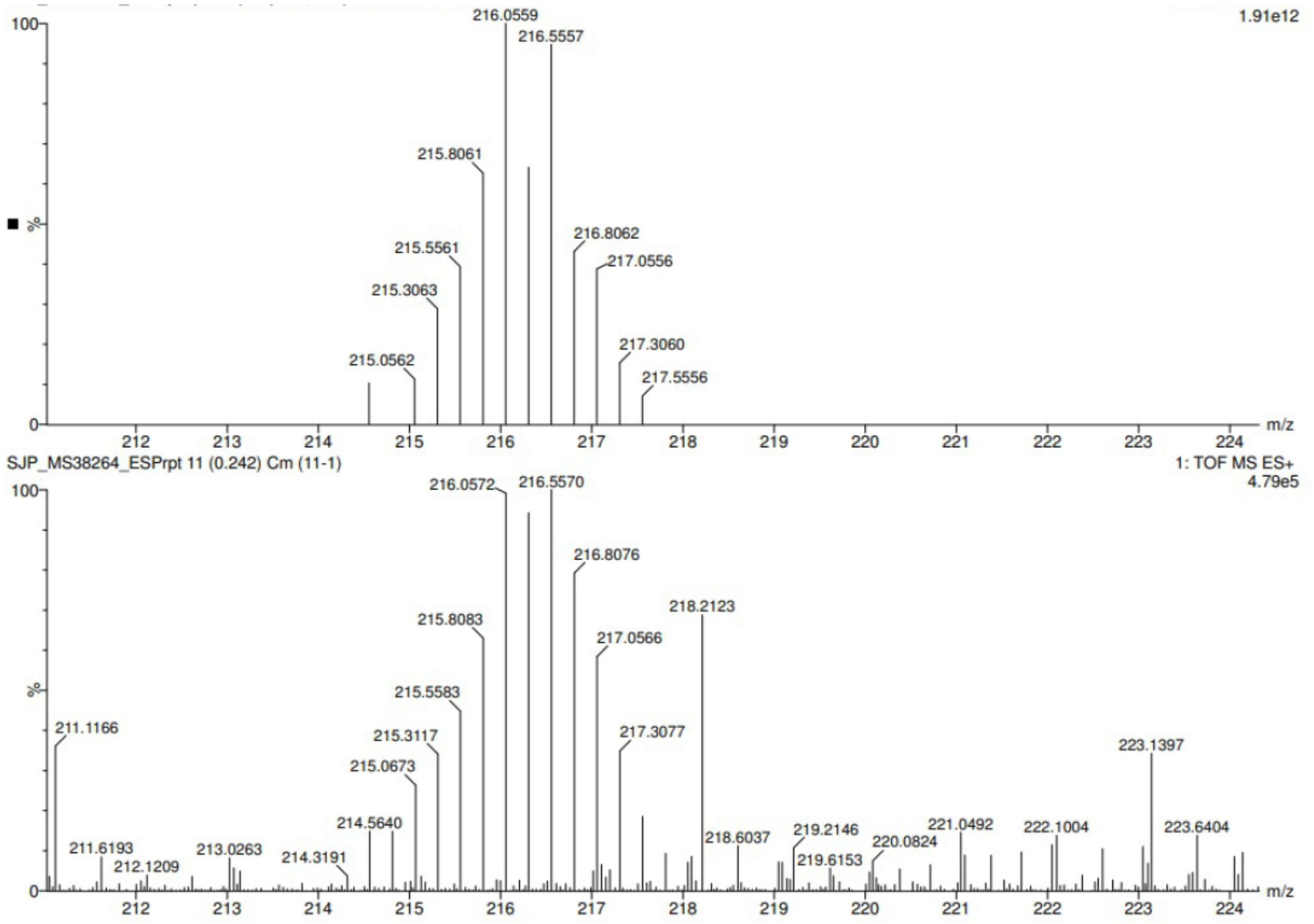

S1.2.4 Synthesis of [Ru)(*p*-cymene)(bipyCl]Cl

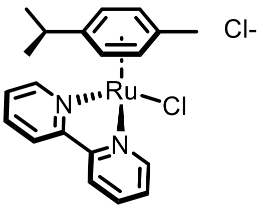

This ligand was prepared as previously described.[7]

[Ru(*p*-cymene)Cl]$_2$ (200 mg, 0.326 mmol, 1.0 eq.) and 2,2'-bipyridine (102.6 mg, 0.657 mmol, 2.0 eq.) was dissolved in CH_2Cl_2/acetone (1:1, 20 mL) and sparged with N_2 for 20 minutes. The reaction was heated to reflux for three hours. The reaction was cooled, the solvent was removed *in vacuo* and the residue was redissolved in H_2O. The mixture was filtered and the solvent removed *in vacuo* to give the title compound as a brown solid (310 mg, 0.65 mmol, >99%). ^{1}H NMR spectrum (300 MHz, 293 K, CD_3CN) 9.41 (dd, $^3J_{HH}$ = 5.7, $^4J_{HH}$ =1.5, 2H, Ar<u>H</u>), 8.37 (dd, $^3J_{HH}$ = 8.2, $^4J_{HH}$ =1.5, 2H, Ar<u>H</u>), 8.17 (dd, $^3J_{HH}$ = 8.1, 7.6, 2H, Ar<u>H</u>), 7.69 (dd, $^3J_{HH}$ = 7.6, 5.7, 2H, Ar<u>H</u>), 5.97 (d, $^3J_{HH}$ = 6.4, 2H, Ar<u>H</u>), 5.77 (d, $^3J_{HH}$ = 6.4, 2H, ArH), 2.64 (sept., $^3J_{HH}$ = 6.9, 1H, ArC<u>H</u>(CH_2)$_2$), 2.20 (s, 3H), 1.01 (d, *J* = 6.9, 6H, ArCH(C<u>H</u>$_2$)$_2$).

Figure S26. ^{1}H NMR spectrum (500 MHz, 293 K, CD_3CN)

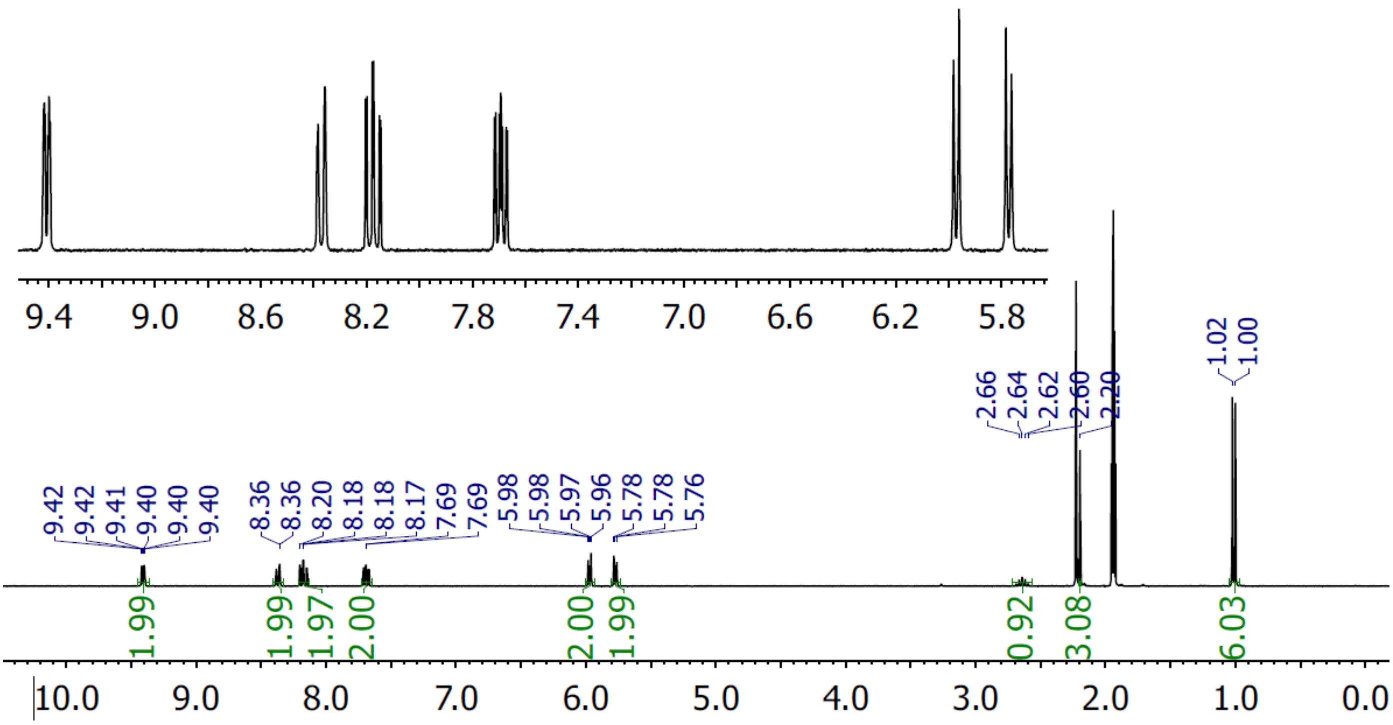

S1.2.5 Synthesis of $[Ru(bipy)_1(L2)_2][PF_6]_6$

Figure S27. ^{1}H NMR spectrum (500 MHz, 293 K, CD_3CN)

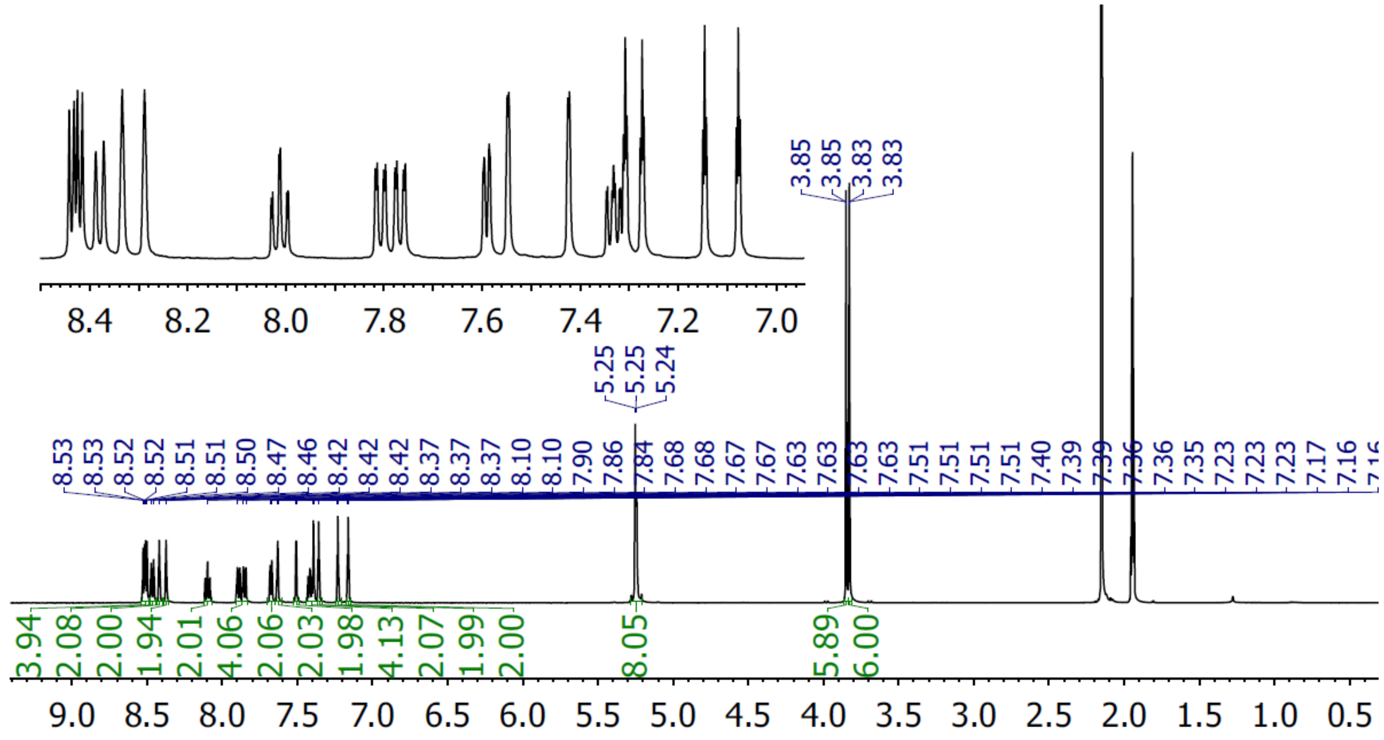

Figure S28.^{13}C NMR spectrum (126 MHz, 293 K, CD_3CN)

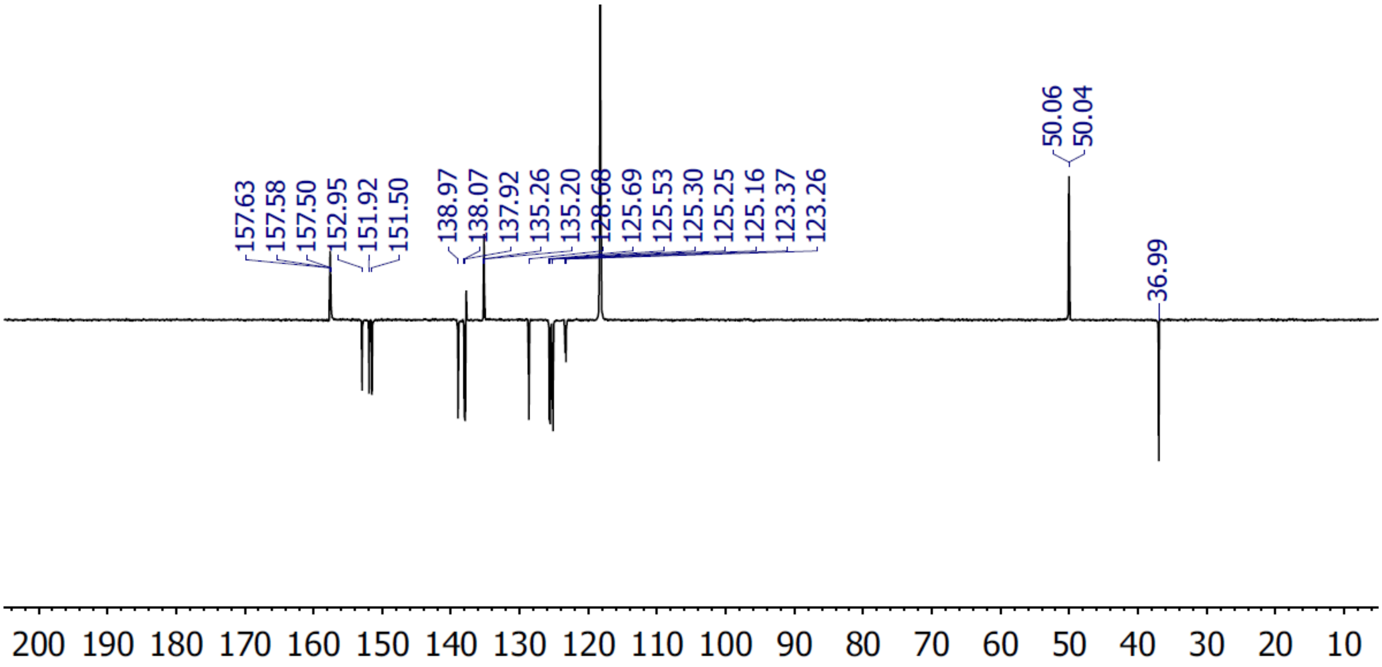

Figure S29. ESI-MS spectrum

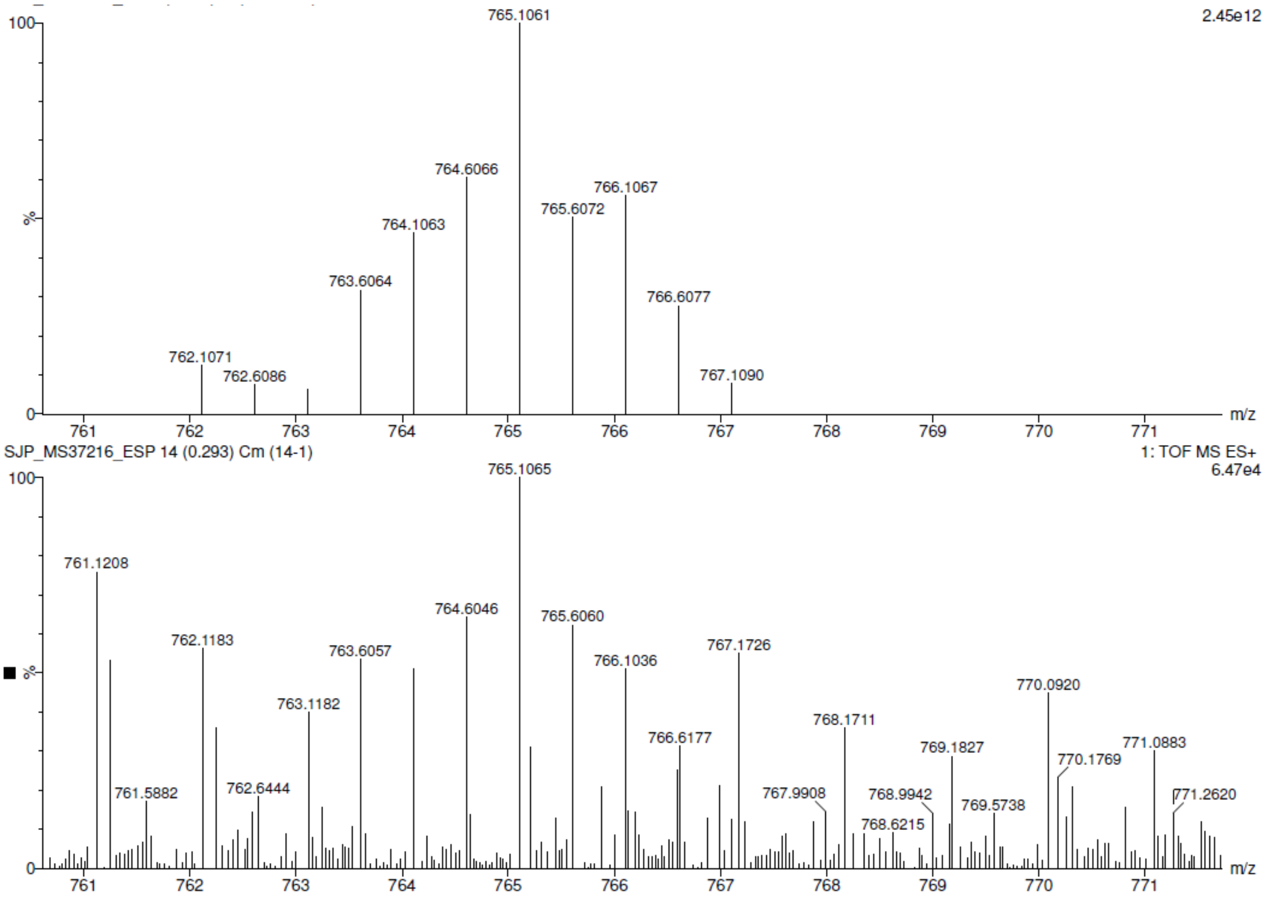

S1.2.6 Synthesis of $[Ru(L2)_3][PF_6]_8$

Figure S30. ^{1}H NMR spectrum (500 MHz, 293 K, CD_3CN)

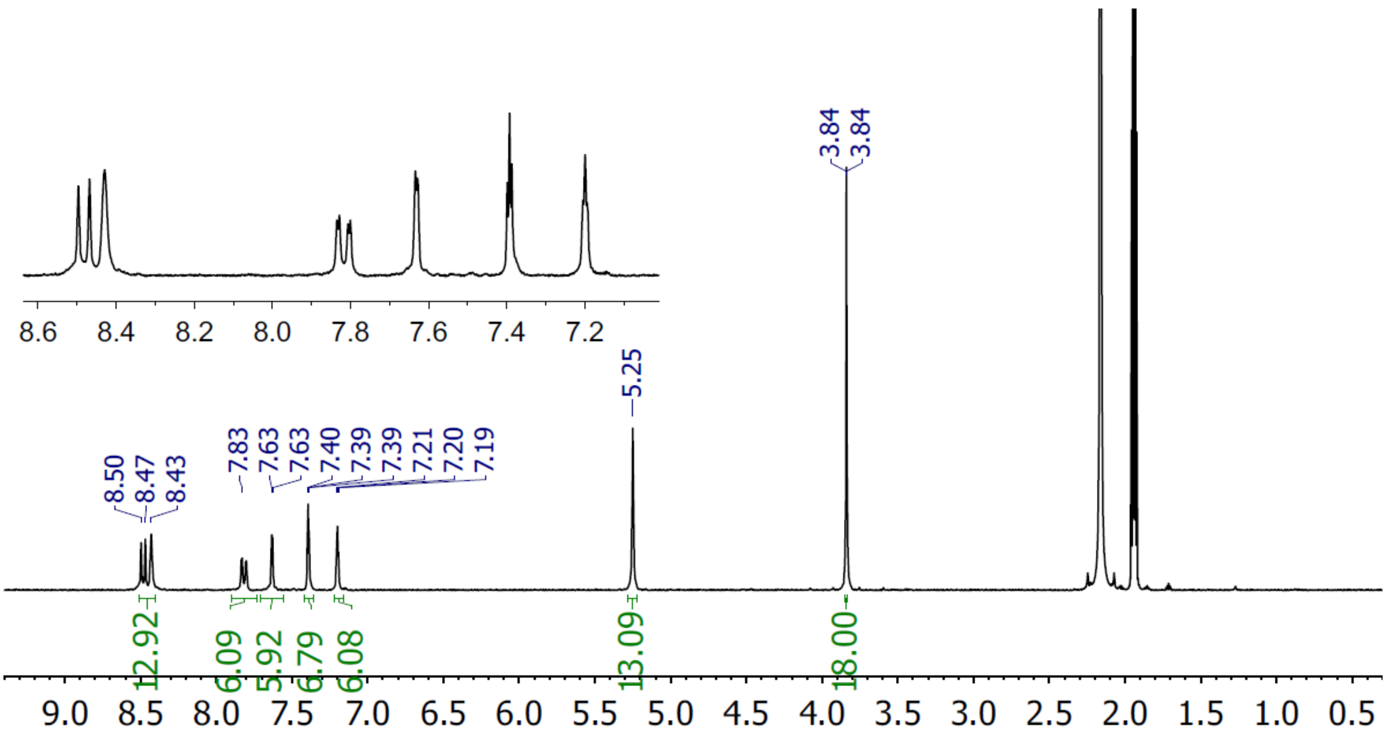

Figure S31. ^{13}C NMR spectrum (126 MHz, 293 K, D_2O)

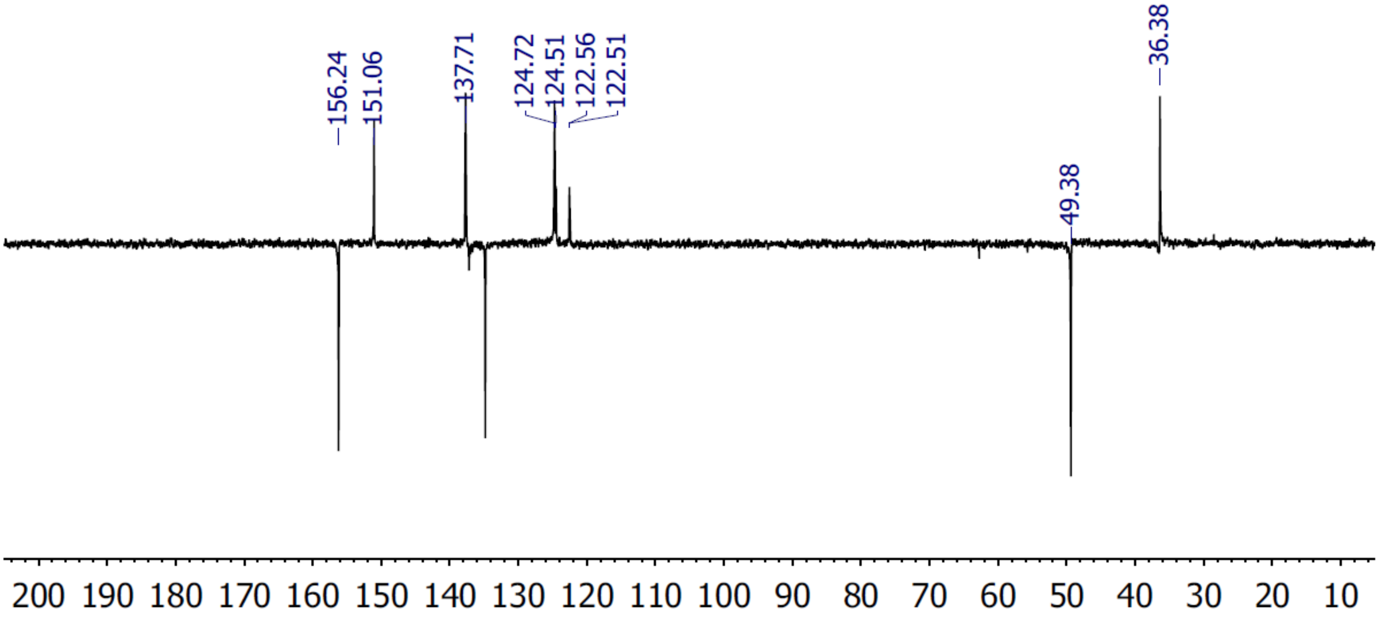

Figure S32. ESI-MS spectrum

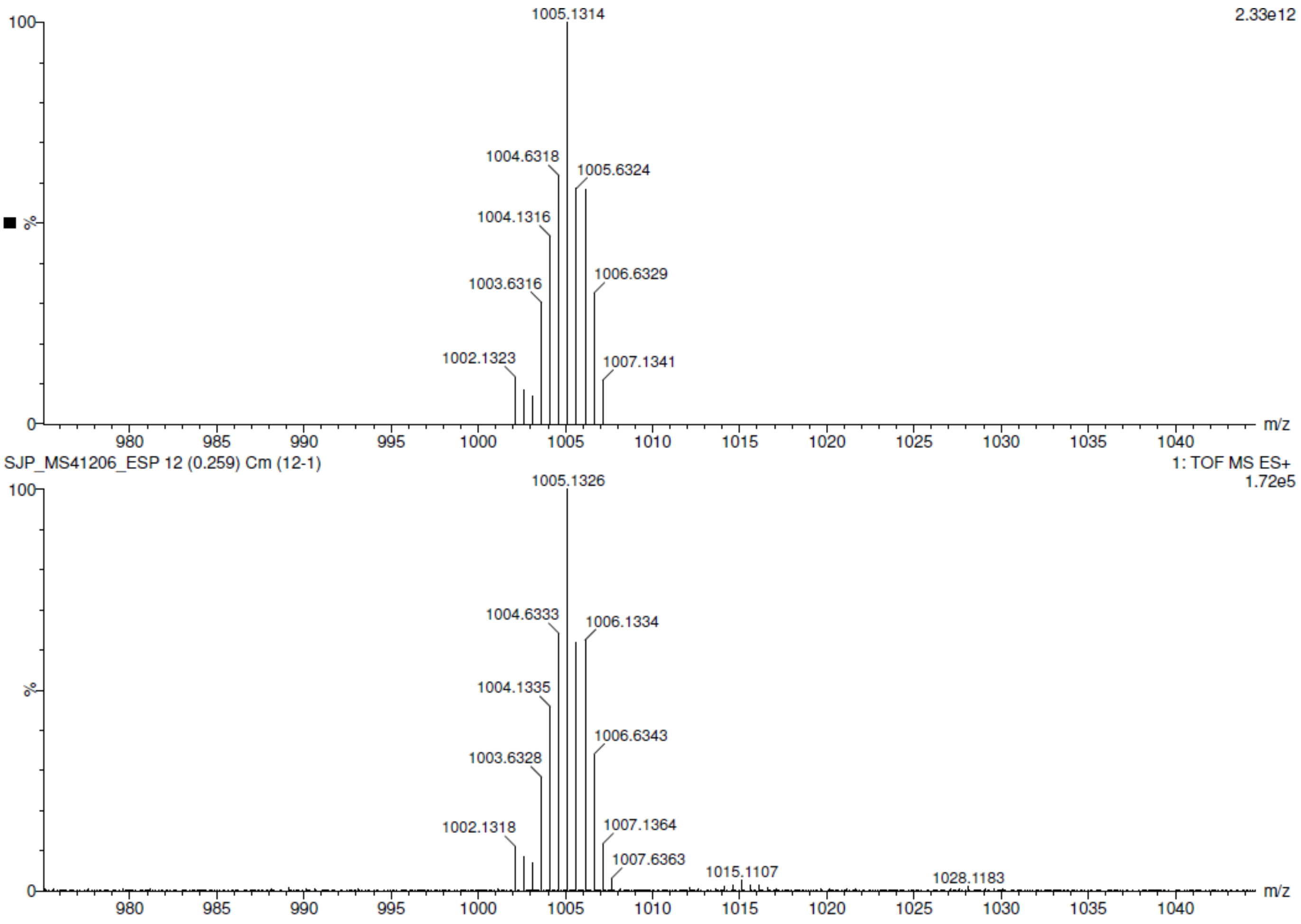

S2. Single-crystal X-ray crystallography

S2.1 Structure of [Ru(bipy)$_2$(L2)][PF$_6$]$_4$

Table S1. Crystal data and structure refinement for **[Ru(bipy)$_2$(L2)][PF$_6$]$_4$.**

**Compound Reference**	**[Ru(bipy)$_2$(L2)$_1$][PF$_6$]$_4$**
Empirical formula	$C_{40}H_{38}F_{24}N_{10}P_4Ru$
Formula weight	1302.70
Temperature/K	200.00(10)
Crystal system	triclinic
Space group	P-1
a/Å	15.1436(5)
b/Å	18.3241(4)
c/Å	19.1567(7)
α/°	99.727(2)
β/°	103.965(3)
γ/°	91.015(2)
Volume/Å^3	5075.0(3)
Z	4
ρ_{calc}g/cm^3	1.705
μ/mm^{-1}	4.913
F(000)	2523.0
Crystal size/mm^3	0.58 × 0.25 × 0.09
Radiation	Cu Kα (λ = 1.54184)
2Θ range for data collection/°	7.474 to 146.768
Index ranges	$-18 \leq h \leq 18$, $-16 \leq k \leq 22$, $-23 \leq l \leq 23$
Reflections collected	37296
Independent reflections	19515 [R_{int} = 0.0770, R_{sigma} = 0.1025]
Data/restraints/parameters	19515/36/1427
Goodness-of-fit on F^2	1.022
Final R indexes [I>=2σ (I)]	R_1 = 0.1052, wR_2 = 0.2658
Final R indexes [all data]	R_1 = 0.1613, wR_2 = 0.3098
Largest diff. peak/hole / e Å^{-3}	2.49/-1.39

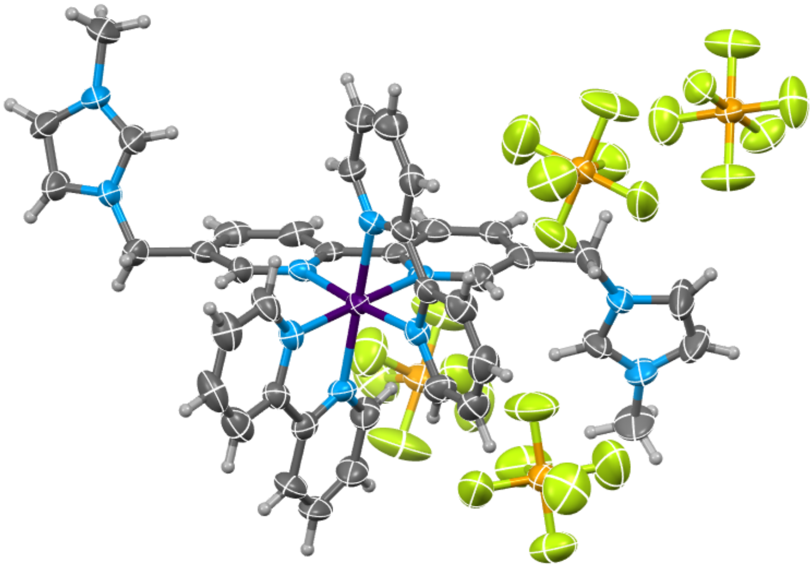

Figure S34. Single crystal X-ray structure of **[Ru(bipy)$_2$(L2)][PF$_6$]$_4$** (ellipsoids plotted at the 50% probability level)

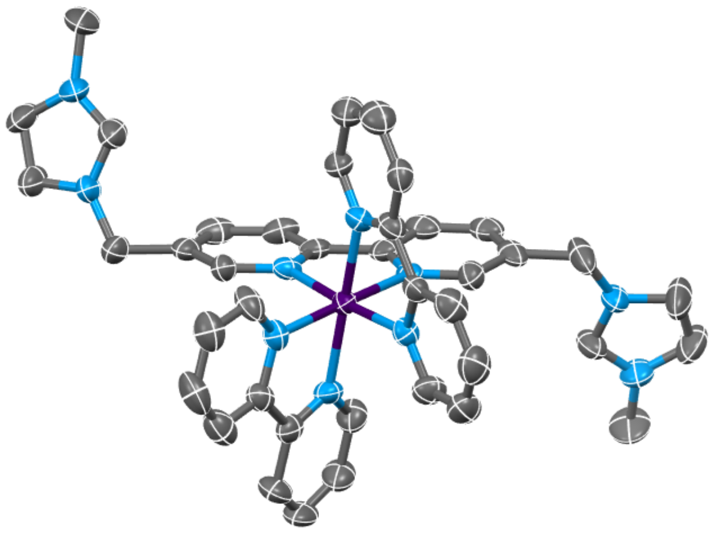

Figure S35. Single crystal X-ray structure of **[Ru(bipy)$_2$(L2)][PF$_6$]$_4$** (ellipsoids plotted at the 50% probability level; H-atoms and counteranions omitted for clarity)

S3 Cyclic Voltammetry

Cyclic voltammetry was performed using a PalmSens4 potentiostat. Experiments were performed using HPLC grade MeCN with an analyte concentration of 1 mM at 293 K, using triply recrystallised [$^{n}Bu_4N$][PF_6] as the supporting electrolyte at 0.1 M concentration. A three-electrode setup was used, consisting of a platinum disc working electrode, a platinum wire counter-electrode and a silver wire pseudo-reference. Solutions were spared for 10 minutes with MeCN saturated stream of nitrogen gas. Voltammograms were referenced to the ferrocene/ferrocenium redox couple measured using the same conditions.

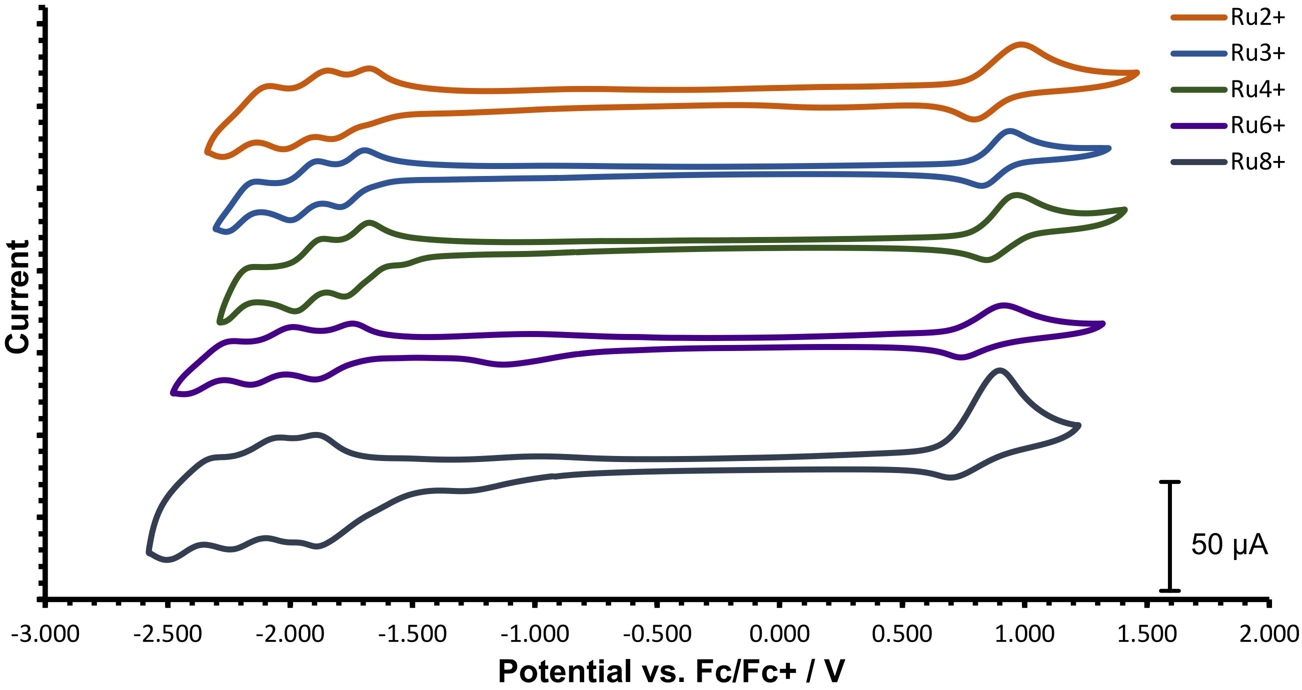

Figure S36. Cyclic voltammograms for the family of ruthenium complexes

S4 Density Functional Theory

HOMO<br>(-12.81 eV)	HOMO-1<br>(-12.95 eV)	HOMO-2<br>(-13.01 eV)	HOMO-3<br>(-13.88 eV)
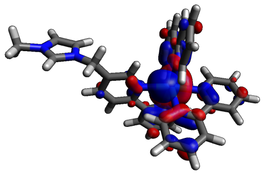	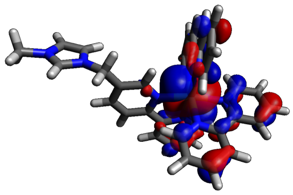	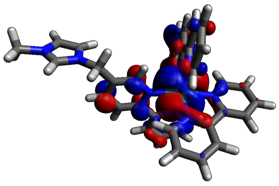	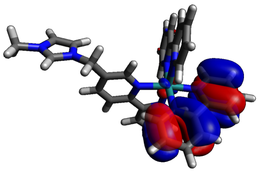
**LUMO<br>(-9.61 eV)**	**LUMO+1<br>(-9.18 eV)**	**LUMO+2<br>(-9.03 eV)**	**LUMO+3<br>(-8.87 eV)**
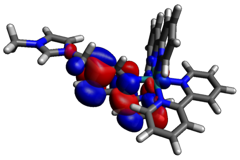	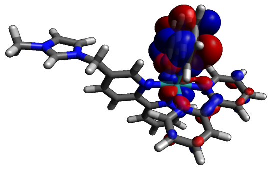	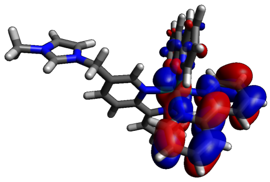	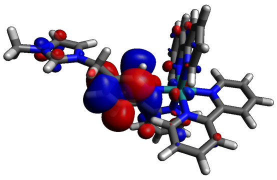

Table S2. Calculated Kohn-Sham molecular orbitals for [Ru(bipy)$_2$(**L1**)]$^{3+}$.

HOMO<br>(-14.56 eV)	HOMO-1<br>(-14.69 eV)	HOMO-2<br>(-14.76 eV)	HOMO-3<br>(-15.52 eV)
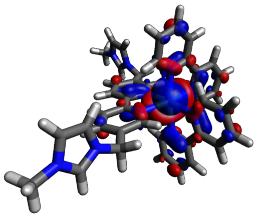	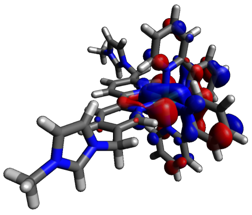	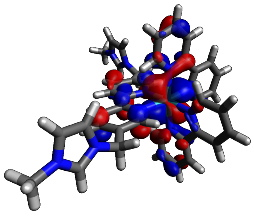	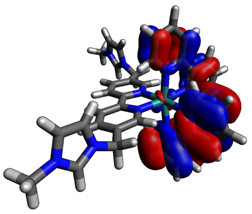
**LUMO<br>(-11.53 eV)**	**LUMO+1<br>(-10.77 eV)**	**LUMO+2<br>(-10.67 eV)**	**LUMO+3<br>(-10.62 eV)**
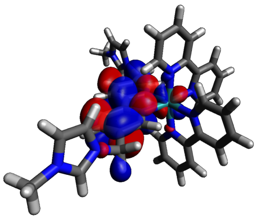	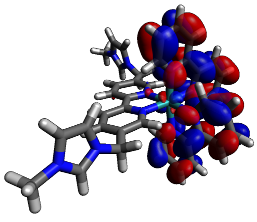	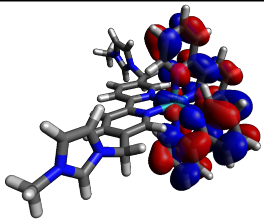	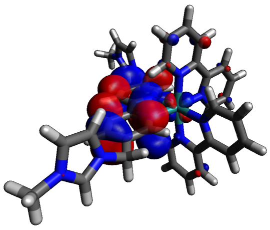

Table S3. Calculated Kohn-Sham molecular orbitals for [Ru(bipy)$_2$(**L2**)]$^{4+}$.

HOMO (-17.87 eV)	HOMO-1 (-18.04 eV)	HOMO-2 (-18.07 eV)	HOMO-3 (-18.74 eV)
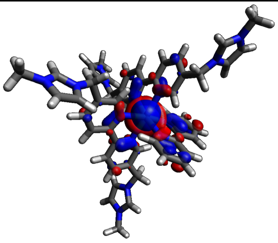	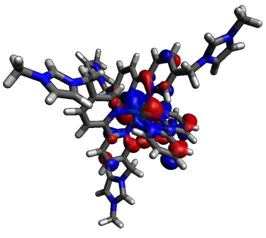	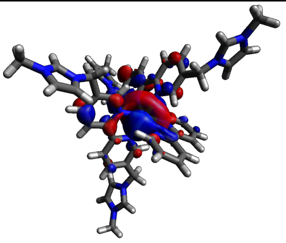	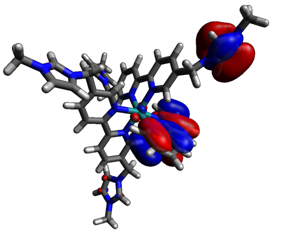
**LUMO (-14.51 eV)**	**LUMO+1 (-14.46 eV)**	**LUMO+2 (-13.93 eV)**	**LUMO+3 (-13.74 eV)**
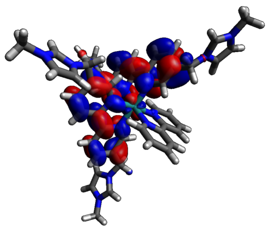	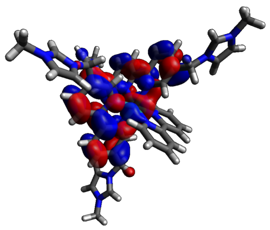	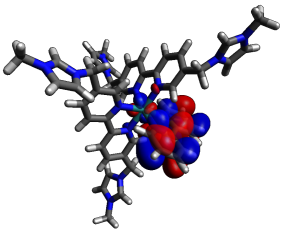	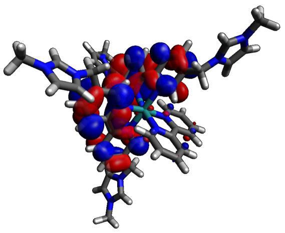

Table S4. Calculated Kohn-Sham molecular orbitals for $[Ru(bipy)(\mathbf{L2})_2]^{6+}$.

HOMO (-21.05 eV)	HOMO-1 (-21.23 eV)	HOMO-2 (-21.23 eV)	HOMO-3 (-21.31 eV)
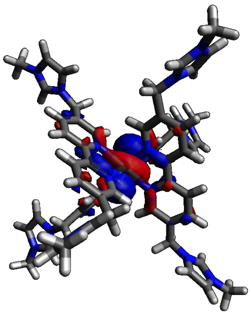	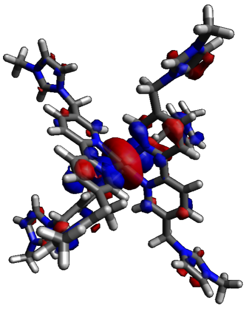	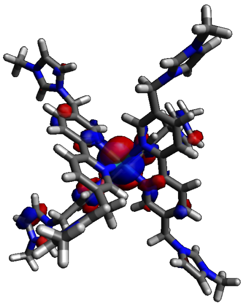	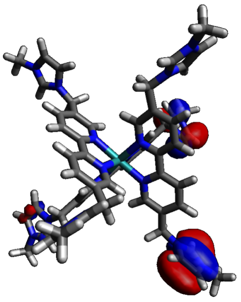
**LUMO (-17.47 eV)**	**LUMO+1 (-17.42 eV)**	**LUMO+2 (-17.41 eV)**	**LUMO+3 (-16.75 eV)**
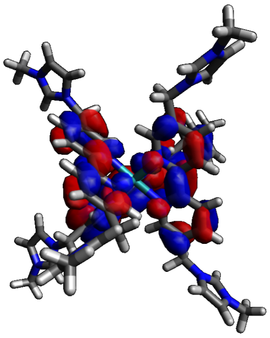	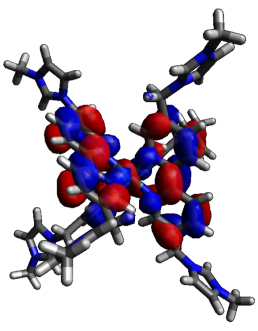	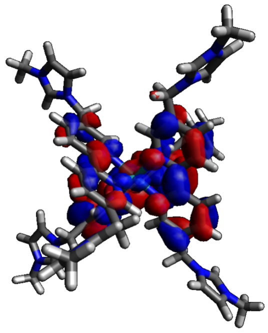	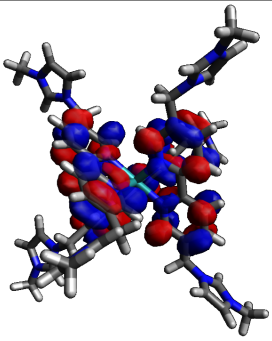

Table S5. Calculated Kohn-Sham molecular orbitals for $[Ru(\mathbf{L2})_3]^{8+}$.

	Moiety Contribution to Orbital (%)				Orbital Contribution to Excited State	
**Orbital**	**Ru (4d)**	**Bipy1**	**Bipy2**	**L1**	**Excited State**	**Contributing Transitions (> 10%)**
**LUMO +4**	0	0	0	99	1 (537 nm f=0.0005)	HOMO → LUMO (97.3%)
**LUMO +3**	3	2	4	92		
**LUMO +2**	5	89	6	0	2 (498 nm f=0.0010)	HOMO -1 → LUMO (83.4%)
**LUMO +1**	4	6	89	1		HOMO -2→ LUMO (13.9%)
**LUMO**	4	1	1	94		
**HOMO**	83	6	5	5	3 (455 nm f=0.0329)	HOMO → LUMO +1 (54.3%)
						HOMO -2 → LUMO (30.0%)
**HOMO -1**	76	11	7	6	4 (452 nm f=0.0562)	HOMO -2 → LUMO (38.8%)
**HOMO -2**	76	4	10	10		
**HOMO -3**	1	97	2	0	5 (431 nm f=0.0020)	HOMO → LUMO +2 (90.6%)
**HOMO -4**	1	1	97	1	6 (424 nm f=0.0058)	HOMO -1 → LUMO +1 (78.6%)

Table S6. A description of the calculated MO contributions, excited state descriptions and their associated transitions for $[Ru(bipy)_2(\mathbf{L1})]^{3+}$ where Bipy1 and Bipy2 are the two distinct bipyridine ligands.

	Moiety Contribution to Orbital (%)				Orbital Contribution to Excited State	
**Orbital**	**Ru (4d)**	**Bipy1**	**Bipy2**	**L2**	**Excited State**	**Contributing Transitions (> 10%)**
**LUMO +4**	3	0	0	97	1 (581 nm f=0.0006)	HOMO → LUMO (97.6%)
**LUMO +3**	3	2	3	91		
**LUMO +2**	6	63	31	0	2 (538 nm f=0.0001)	HOMO -1 → LUMO (97.4%)
**LUMO +1**	2	33	64	1		
**LUMO**	5	0	0	94		
**HOMO**	83	6	6	5	3 (478 nm f=0.0811)	HOMO -2 → LUMO (93.6%)
**HOMO -1**	76	11	10	3	4 (425 nm f=0.0006)	HOMO → LUMO +1 (87.4%)
**HOMO -2**	76	6	6	12		
**HOMO -3**	1	80	19	0	5 (420 nm f=0.0005)	HOMO → LUMO +2 (88.4%)
**HOMO -4**	2	18	80	0	6 (397 nm f=0.0177)	HOMO -1 → LUMO +1 (59.7%)
						HOMO -2 → LUMO +2 (22.4%)
						HOMO -2 → LUMO +1 (22.4%)

Table S7. A description of the calculated MO contributions, excited state descriptions and their associated transitions for $[Ru(bipy)_2(\mathbf{L2})]^{4+}$ where Bipy1 and Bipy2 are the two distinct bipyridine ligands.

	Moiety Contribution to Orbital (%)				Orbital Contribution to Excited State	
**Orbital**	**Ru (4d)**	**Bipy**	**$L2_1$**	**$L2_2$**	**Excited State**	**Contributing Transitions (> 10%)**
**LUMO +4**	4	1	50	45	1 (497 nm f=0.0001)	HOMO → LUMO +1 (87.9%)
**LUMO +3**	2	3	45	49		
**LUMO +2**	4	94	1	1	2 (493 nm f=0.0005)	
**LUMO +1**	5	0	61	34		HOMO → LUMO (88.9%)
**LUMO**	3	1	35	62		
**HOMO**	83	7	5	5	3 (460 nm f=0.0001)	HOMO -1 → LUMO +1 (57.6%)
						HOMO -2 → LUMO (26.1%)
						HOMO -1 → LUMO (13.9%)
**HOMO -1**	78	12	6	5	4 (455 nm f=0.0006)	HOMO -1 → LUMO (66.3%)
**HOMO -2**	78	5	8	9		HOMO -2 → LUMO +1 (20.3%)
**HOMO -3**	1	57	1	41	5 (437 nm f=0.1192)	HOMO -2 → LUMO (68.1%)
						HOMO -1 → LUMO +1 (27.7%)
**HOMO -4**	1	39	1	59	6 (420 nm f=0.0372)	HOMO -2 → LUMO +1 (71.7%)
						HOMO -1 → LUMO (16.4%)

Table S8. A description of the calculated MO contributions, excited state descriptions and their associated transitions for $[Ru(bipy)(\mathbf{L2})_2]^{6+}$ ($L2_1$ and $L2_2$ are the two distinct modified ligands; bipy = bipyridine).

	Moiety Contribution to Orbital (%)				Orbital Contribution to Excited State	
**Orbital**	**Ru (4d)**	**$L2_1$**	**$L2_2$**	**$L2_3$**	**Excited State**	**Contributing Transitions (> 10%)**
**LUMO +4**	3	33	9	55	1 (458 nm f=0.0000)	HOMO → LUMO +1 (91.7%)
**LUMO +3**	2	34	32	32		
**LUMO +2**	5	48	33	14	2 (455 nm f=0.0000)	HOMO → LUMO +2 (88.7%)
**LUMO +1**	5	1	39	55		
**LUMO**	1	47	25	28	3 (448 nm f=0.0002)	HOMO → LUMO (93.4%)
**HOMO**	83	5	6	6	4 (425 nm f=0.0002)	HOMO -2 → LUMO +1 (42.9%)
						HOMO -1 → LUMO +2 (40.1%)
**HOMO -1**	70	7	12	11	5 (419 nm f=0.0005)	HOMO -1 → LUMO (59.4%)
**HOMO -2**	72	16	6	7		HOMO -2 → LUMO +1 (18.7%)
**HOMO -3**	0	41	58	2	6 (418 nm f=0.0008)	HOMO -2 → LUMO (43.5%)
						HOMO -2 → LUMO +2 (25.0%)
						HOMO -1 → LUMO +1 (18.5%)
**HOMO -4**	0	23	23	54	7 (407 nm f=0.0979)	HOMO -2 → LUMO (46.6%)
						HOMO -1 → LUMO +1 (24.4%)
						HOMO -2 → LUMO +2 (14.6%)

Table S9. A description of the calculated MO contributions, excited state descriptions and their associated transitions for $[Ru(\mathbf{L2})_3]^{8+}$ ($L2_1$, $L2_2$, and $L3_3$ are the three distinct modified ligands).

Table S10 Computed values for the $S_0 \rightarrow T_1$ energy gap from vertical TD-DFT calculations.

**Complex**	**$S_0 \rightarrow T_1$ (nm)**
$[Ru(bipy)_2(\mathbf{L1})]^{3+}$	573
$[Ru(bipy)_2(\mathbf{L2})]^{4+}$	621
$[Ru(bipy)(\mathbf{L2})_2]^{6+}$	531
$[Ru(\mathbf{L2})_3]^{8+}$	501

S5 Ion pairing effects

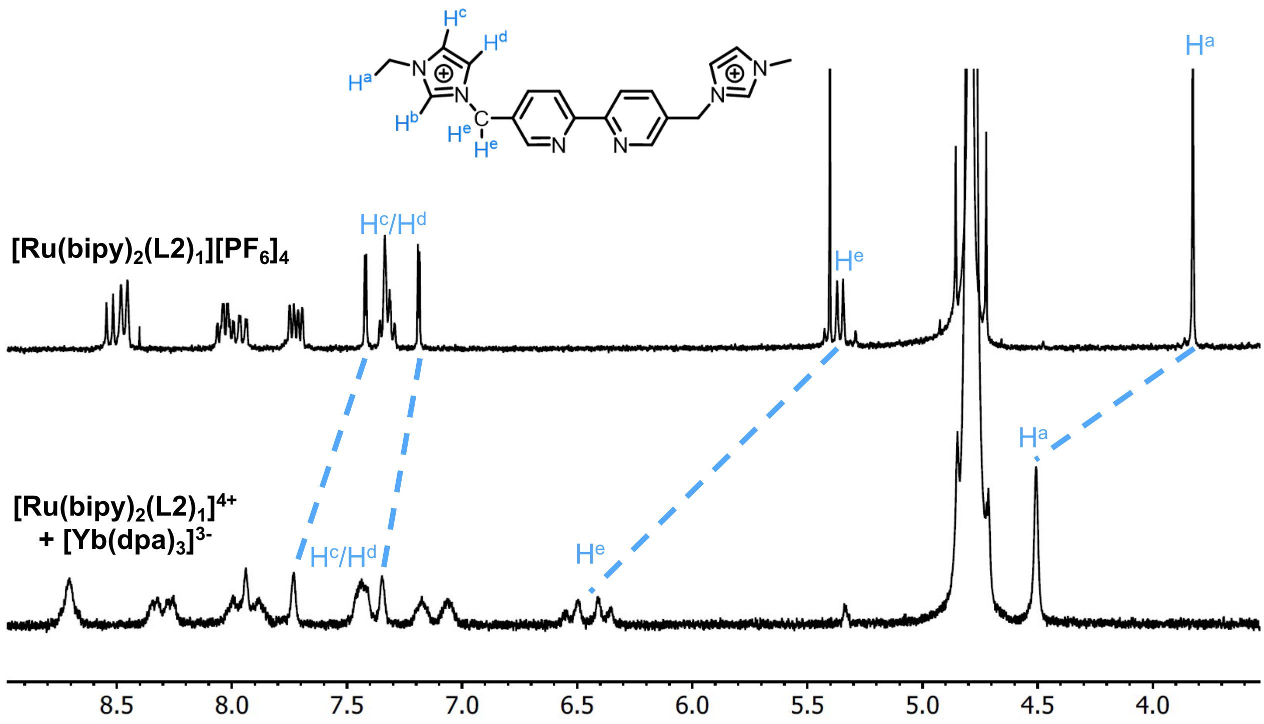

Figure S37. ^{1}H NMR (300 MHz, 293 K, 0.1M KNO_3 D_2O) spectra of $[Ru(bipy)_2(\mathbf{L2})][PF_6]_4$ (top) and a 1:1 mixture of $[Ru(bipy)_2(\mathbf{L2})][PF_6]_4$ and $Na_3[Yb(dpa)_3]$

References

[1] Charbonnière, L. J.; Weibel, N.; Ziessel, R. F. 5‘-Substituted-6-Carboxylic-2,2‘-Bipyridine Acid: A Pivotal Architecton for Building Preorganized Ligands. *J. Org. Chem.* **2002**, *67* (11), 3933–3936.

[2] Bai, S.-T.; Bheeter, C. B.; Reek, J. N. H. Hydrogen Bond Directed Ortho-Selective C−H Borylation of Secondary Aromatic Amides. *Angew. Chem. Int. Ed.* **2019**, *58* (37), 13039–13043.

[3] Chung, C. W. Y.; Toy, P. H. Multipolymer Reaction System for Selective Aerobic Alcohol Oxidation: Simultaneous Use of Multiple Different Polymer-Supported Ligands. *J. Comb. Chem.* **2007**, *9* (1), 115–120.

[4] Kim, E. H.; Hong, S.; Lee, J. M.; Lee, D. N.; Jun, Y. M.; Lee, W.-Y.; Kim, B. H. Synthesis and Properties of Electrochemiluminescent Dinuclear Ru(II) Complexes Assembled with Ester-Bridged Bis(Bipyridine) Ligands. *Inorganica Chim. Acta* **2009**, *362* (5), 1577–1584.

[5] Telfer, S. G.; Bernardinelli, G.; Williams, A. F. Iron and Cobalt Complexes of 5,5′-Di(Methylene-N-Aminoacidyl)-2,2′-Bipyridyl Ligands: Ligand Design for Diastereoselectivity and Anion Binding. *Dalton Trans* **2003**, No. 3, 435–440.

[6] Rama, G.; Ardá, A.; Maréchal, J.-D.; Gamba, I.; Ishida, H.; Jiménez-Barbero, J.; Vázquez, M. E.; Vázquez López, M. Stereoselective Formation of Chiral Metallopeptides. *Chem. – Eur. J.* **2012**, *18* (23), 7030–7035.

[7] P.K, A.; Paira, P. Luminescent Anticancer Ru(II)-Arenebipyridine and Phenanthroline Complexes: Synthesis, Characterization, DFT Studies, Biological Interactions and Cellular Imaging Application. *J. Inorg. Biochem.* **2020**, *208*, 111099.
